# Efficient Approaches to Six-Membered Polyazacyclic Compounds—Part 3: C—H Functionalization of Heterocycles

**DOI:** 10.3390/molecules31060959

**Published:** 2026-03-12

**Authors:** Yuliya Yu. Titova, Andrey V. Ivanov

**Affiliations:** A.E. Favorsky Irkutsk Institute of Chemistry, Siberian Branch of the Russian Academy of Sciences, Favorsky Str. 1, 664033 Irkutsk, Russia; ivanov@irioch.irk.ru

**Keywords:** C−H functionalization, six-membered heterocycles, polyazacyclic compounds

## Abstract

The review summarizes the literature data on the C−H functionalization of six-membered polyaza heterocycles, specifically diazine and triazine skeletons, which are important in medicine and pharmacology, as examples. The analysis covers the works published mainly over the last 20 years. The review focuses on strategies involving the use of transition metal-based catalysts or organic oxidants, where the nature of the *N*-heterocycles and the substrate molecules can be exploited to control regioselectivity. Each of these strategies has certain advantages as well as serious disadvantages and limitations. In addition to the experimental procedures, mechanistic schemes are discussed to provide a deeper understanding of the reactions described. The material presented allows us to draw the unambiguous conclusion that C−H bond functionalization processes are of crucial importance in the synthesis of molecules that exhibit a wide range of biological activity.

## 1. Introduction

**Figure 1:**
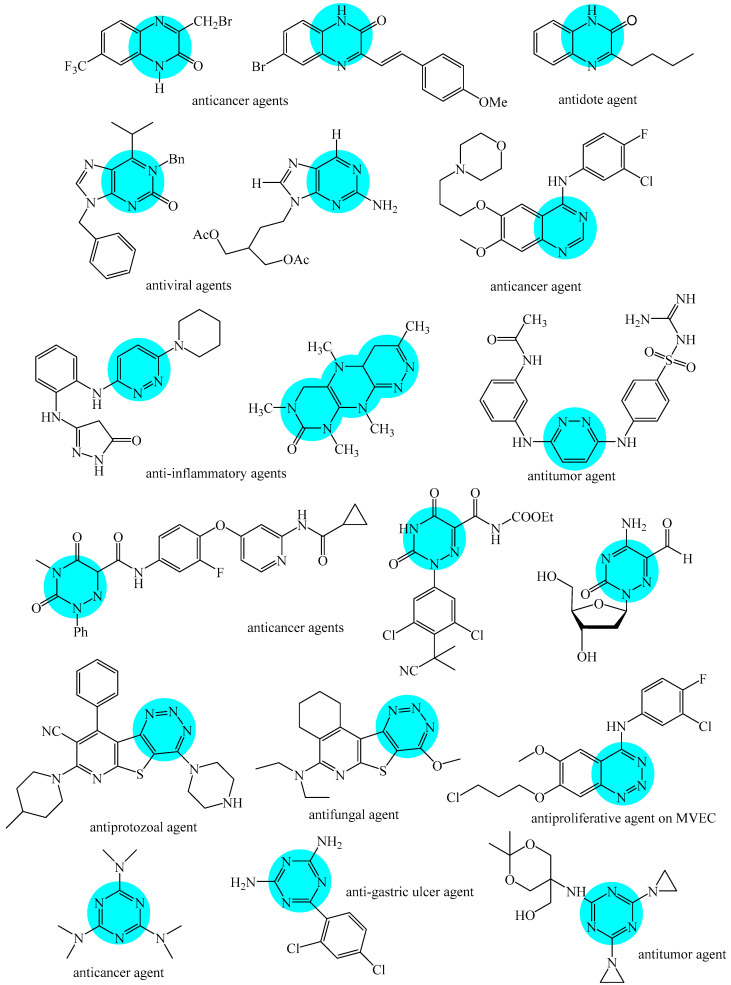
Examples of structures containing polynitrogen six-membered frameworks (it are highlighted in blue) and their therapeutic target (where MVEC = microvascular endothelial cells), according to [84,85,86,87,88,89,90,91,92,93,94,95,96,97].

Nitrogen-containing heterocycles are the most well-known and therapeutically valuable structural fragments in medicinal chemistry and pharmaceuticals. They account for more than 75% of drugs approved by the FDA [1]. Nitrogen-based heterocycles, for example, are structural motifs found in medicines such as ellipticin, theophylline, quinine, papaverine, morphine, novocaine, codeine, and emetine, which have had a significant impact on pharmaceutical science [2,3,4,5,6,7,8,9,10,11,12,13,14]. Polyaza structures occupy a leading position among the many nitrogen heterocyclic compounds in organic chemistry due to their structural diversity and biological activity [15,16,17,18,19,20,21,22]. These are a subclass of six-membered heterocycles containing two or more nitrogen atoms in their ring structure (Figure 1).

Many papers describe various aspects of the synthesis of heterocyclic compounds [23,24,25,26,27,28,29,30,31,32]. For instance, diverse methods for the preparation of six-membered polyaza aromatic heterocycles [33] and their nonaromatic analogs [34], as well as intriguing approaches to the saturation of heteroaromatic compounds [35] have been published previously. The next logical step is to conduct a comparative analysis of the existing scientific literature on the functionalization of six-membered polyaza frameworks. Such functionalization could provide chemists engaged in drug design and the creation of structures suitable for the targeted delivery of pharmaceuticals, artificial nucleotides, bioactive agents and agrochemicals, as well as for the creation of ligands, sensors, pigments, dyes and functional materials with virtually unlimited opportunities [16,17,25,36,37,38,39,40,41,42,43,44,45,46,47,48,49]. However, the number of known strategies for activation of the C–H bonds in *N*-heterocycles is limited due to the thermodynamic stability, kinetic inertness and ability to deactivate catalysts containing nitrogen-rich aromatic heterocycles. Most studies to date have focused on the functionalization of the C–H bond adjacent to nitrogen by the addition of carbon-centered radicals under oxidative conditions, a process known as the Minisci reaction. Although the classic Minisci alkylation reaction involves alkylcarboxylic acids and halides, this methodology has experienced a notable resurgence in popularity in recent years, providing increasingly efficient methods for the synthesis of substituted *N*-heteroarenes. Other possible transformations have also been described, including borylation [50], acylation [51], alkoxylation [52], etc.

It should be noted that, due to the presence of various types of ligands [53,54,55,56,57,58,59,60,61,62], the implementation of non-covalent interactions [63,64,65,66,67], the influence of co-catalytic components [68,69,70,71], and the formation of highly reactive structures in situ [72,73,74,75], multifunctional metal-based catalytic systems developed in recent years are capable of acting as “molecular scalpels”, ensuring exceptional precision of functionalization reactions. These systems could also potentially serve as the basis for formulating the fundamentals of the theory of directed C–H functionalization of polyaza six-membered heterocycles.

Conversely, photochemical functionalization studies [55,76,77,78,79,80,81,82,83] have made it possible to create powerful, versatile and effective methods for the activation of C–H bonds in polyaza six-membered heterocycles that are conditionally “green”.

This review summarizes the potential for the C−H functionalization of six-membered polyaza heterocycles containing two or three nitrogen atoms without altering or destroying the original heterocycle. The review covers the literature published over the past 20 years. Unfortunately, we were unable to find any examples of work describing the activation of C—H bonds in heterocycles containing four nitrogen atoms. This is probably because the known synthetic methods [33,34] do not permit the formation of such structures. Given the variety of C—H functionalization options for heterocycles, the review is divided into several chapters, each devoted to a specific type of transformation. These include:

(1) The formation of a new C−C bond between carbon atoms in the heterocyclic molecule and a fragment of the added substrate (e.g., alkylation, arylation, acetylation, annulation, carboxylation reactions, etc.);
(2) The formation of a new C−N bond between a carbon atom in the heterocyclic molecule and a nitrogen atom in the substrate molecule (e.g., amination, amidation, or oxidative amidation reactions);
(3) The formation of a new C−O bond between a carbon atom in the heterocyclic molecule and an oxygen atom in the substrate molecule (alkoxylation reactions), etc.

In each chapter, the information is presented in accordance with the sequence of nitrogen atoms in the six-membered ring. In other words, the chapter begins with a description of the reactions with 1,2-diazines. This is followed by a sequential examination of the transformations of 1,3- and 1,4-diazines, as well as 1,2,3-, 1,2,4-, and 1,3,5-triazines. If there are no examples in the literature of any of the types of heterocyclic compounds, then, while our review cannot be considered comprehensive, the next model examples in the above series will be covered. In addition, for each specific type of *N*-heterocycle, the progression from simple to complex is logical; the discussion begins with the metal-free methods and ends with metal-catalyzed reactions. We believe that presentation of the material in this way highlights the need to replace classical methods, which mostly rely on the transition and/or noble metal complexes, with metal-free approaches. The latter will not only simplify the isolation of the target products, making them more “green”, they will also free up certain metals for use in areas where they cannot yet be replaced.

## 2. Methods for the Formation of C−C Bonds

### 2.1. Alkylation Reactions

#### 2.1.1. Metal-Free Alkylation

##### Pyridazines

**Figure 2:**
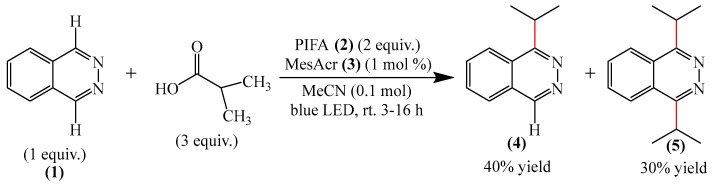
C−H alkylation of heteroaromatics via hypervalent iodine-promoted decarboxylation, according to [98]. (From here onwards, targeted new bonds are highlighted in red).

One example was reported [98] of metal-free photo-oxidative–reductive C—H alkylation of a heteroaromatic compound containing a pyridazine fragment (**1**), which was irradiated with 32 W blue LED light (λ_max_ = 455 nm) (Figure 2). The solvent used was MeCN, the hypervalent iodine-containing oxidant was bis(trifluoroacetoxy)iodo benzene (PIFA) (**2**), and the photocatalyst was 9-mesityl-10methyl acridinium perchlorate (MesAcr) (**3**). 1-Isopropylphthalazine (**4**) and 1,4-diisopropylphthalazine (**5**) were registered as products with yields of 40% and 30%, respectively.

**Figure 3:**
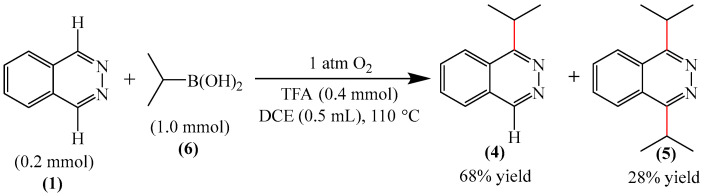
Molecular oxygen-mediated Minisci-type radical alkylation of phthalazine, according to [99].

Liu and Zhang described [99] a simple, environmentally friendly method for alkylation of a pyridazine fragment (**1**) by auto-oxidation of isopropylboronic acid (**6**) (Figure 3). It was found that the formation of the target 1-isopropylphthalazine (**4**) was accompanied by the generation of a bis-alkylation product (**5**) (28%). Unlike most Minisci alkylation reactions described in this section, which require a stoichiometric amount of oxidizing agents and/or transition metal salts/complexes, this method is metal-free and eco-friendly. The use of molecular oxygen (1 atm), an environmentally friendly oxidant, can be considered an advantage of this method.

##### Pyrimidine

**Figure 4:**
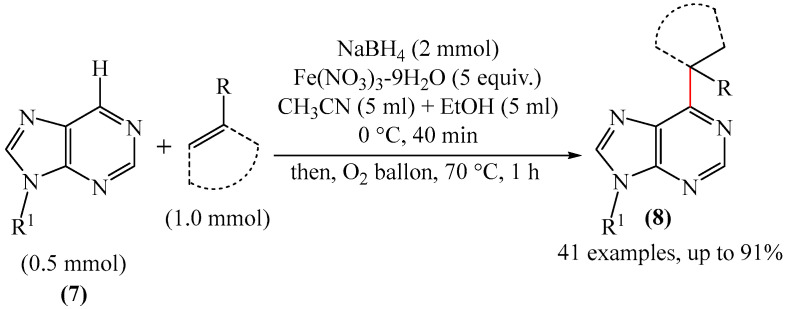
C(sp^2^)−C(sp^3^) bond construction between purines and alkenes to synthesize C6-alkylpurines and purine nucleosides using O_2_ as the oxidant, according to [68]. R = H, Me, Et, and others; R^1^ = Bn, Bu, Pr, C_6_H_5_, C_6_H_5_-CH_2_, and others.

An example of the methodology for alkylation of pyrimidine-containing fragments **7** with the formation of structures **8** was described [68] (Figure 4). The reaction was carried out in the presence of complex catalytic systems based on NaBH_4_ and compounds such as nitrates, sulfates, and chlorides of metals such as silver, potassium, nickel, copper, and iron, including crystallohydrates. The most effective composition was found to be Fe(NO_3_)_3_·9H_2_O/NaBH_4_. In addition, O_2_ was employed as a green oxidant.

The alkylation mechanism presented in [68] raises many questions. According to the data provided, NaBH_4_ and Fe(NO_3_)_3_·9H_2_O do not affect each other in the reaction system. However, this is inconsistent with the data previously obtained by Glave [100,101]. As the authors do not provide convincing evidence of the implementation of the mechanism they postulate—for example, based on spectroscopic methods—it can be assumed that other mechanistic routes may be implemented in addition to those described in [68].

**Figure 5:**
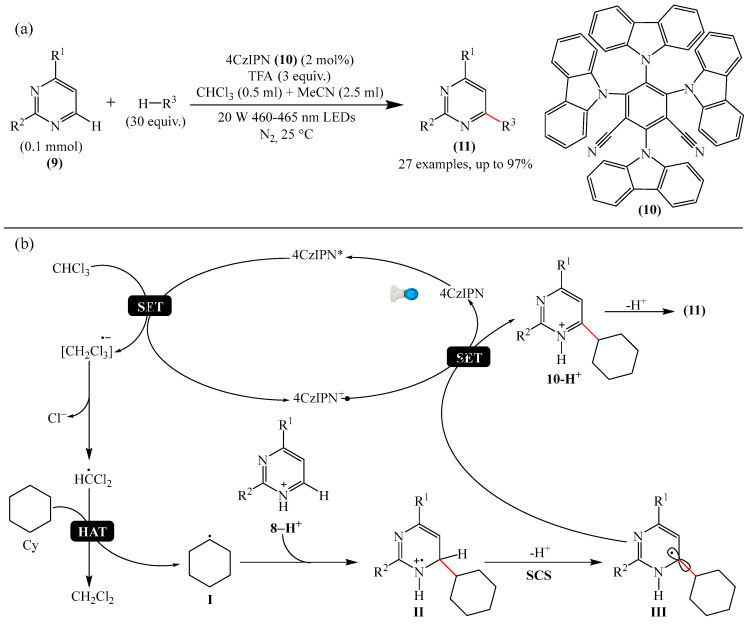
(**a**) Visible light-induced C(sp^3^)-H activation for Minisci alkylation of pyrimidines, and (**b**) plausible mechanism, according to [102]. R^1^ = R^2^ = H, Me, Cy, C_6_H_5_, Cl, and others; R^3^ = C_6_H_5_, C_6_H_5_-CH_2_-, HO-CH_2_-, and others.

Zhang et al. [102] documented a highly efficient Minisci alkylation of pyrimidines (**9**) using visible light and simple starting materials such as (cyclo)alkanes, ethers, alcohols, esters, and amides, via hydrogen atom transfer (HAT)-mediated activation of C(sp^3^)-H bonds (Figure 5a). Monitoring of the optimal reaction conditions demonstrated that the best visible-light photocatalyst was the substituted benzene-1,3-dicarbonitrile (4CzIPN) (**10**), and the best photoreductable molecule was chloroform.

The mechanism proposed by the authors is shown in Figure 5b. Initially, 4CzIPN is transformed to the excited state 4CzIPN*, in which the one-electron reduction of CHCl_3_ generates the radical CHCl_2_, Cl^—^ and 4CzIPN^+^. The CHCl_2_ radical then abstracts a hydrogen atom from cyclohexane (Cy) to form a cyclohexyl radical (I) and CH_2_Cl_2_. Next, the nucleophilic cyclohexyl radical (I) is added to the protonated **8**-H^+^ to produce intermediate II. Deprotonation followed by a spin center shift (SCS) converts II to III. This ultimately leads to the formation of the substituted product **11** via SET oxidation with simultaneous regeneration of the photocatalyst in the ground state, 4CzIPN, thus completing the photo-oxidation–reduction catalytic cycle. Evidence for this mechanistic scheme has been obtained by NMR spectroscopy and chromatographic mass spectrometry.

##### Pyrazines

**Figure 6:**
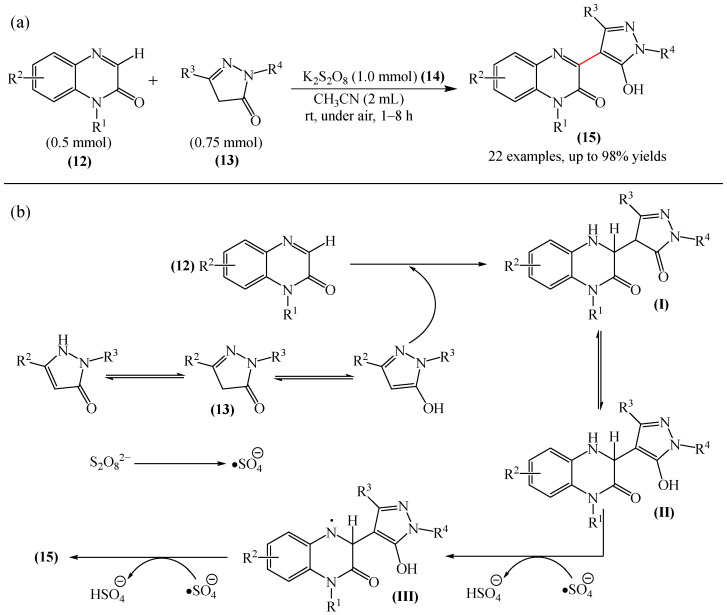
(**a**) Reaction of quinoxalinones with pyrazolones, (**b**) possible mechanism, according to [103]. R^1^ = H, Me, NO_2_, Cl, Br; R^2^ = H, Et, OEt, *n*-C_5_H_11_, *i*-Pr, and others; R^3^ = Me, *n*-Pr, *i*-Pr, C_6_H_5_; R^4^ = C_6_H_5_, 4-ClC_6_H_4_, 2-ClC_6_H_4_, 4-MeOC_6_H_4_, and others.

A highly efficient, transition metal-free oxidative chemo- and regioselective coupling of the C–C bonds in the quinoxalinones (**12**) and the pyrazolones (**13**) was implemented [103]. The reaction was carried out in the presence of K_2_S_2_O_8_ (**14**) under mild conditions to afford various hydroxypyrazolylquinoxalinones (**15**) in moderate to excellent yields in a short reaction time (Figure 6a).

A tentative reaction mechanism is shown in Figure 6b. First, the pyrazolone (**13**) attacks the quinoxalinone substrate (**12**) at the most electrophilic *C3* position to produce intermediate compound I (which is a tautomer of II). Cleavage of the persulfate anion (S_2_O8_2_^−^) leads to the formation of radical intermediate III and, ultimately, to the corresponding product in a completely chemo- and regioselective manner. In addition, it has been found [103] that the oxidation of intermediate I or II to the product could occur in an O_2_ atmosphere or in air (in the absence of a persulfate oxidant), albeit with significantly lower efficiency (yield of about 20–25%) and a much longer reaction time. Unfortunately, as with many subsequent examples, the authors proposed a tentative mechanism based on previously described data and an analysis of the products formed during target transformations.

**Figure 7:**
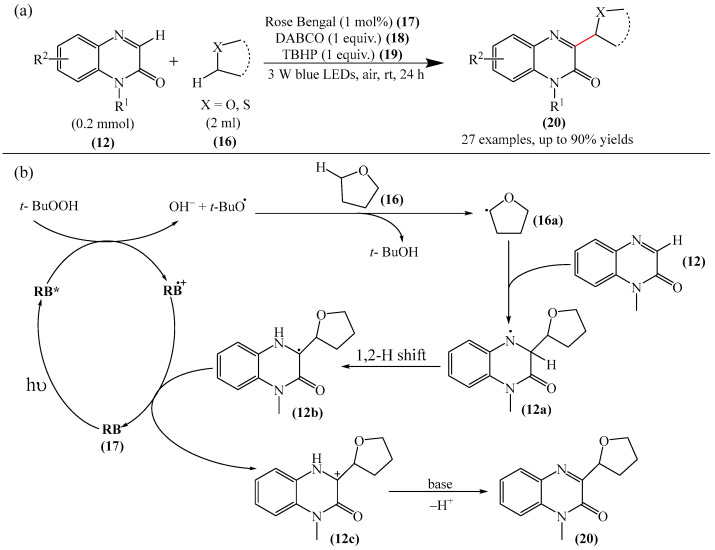
(**a**) Visible light-induced C−H/C−H cross-dehydrogenative coupling of quinoxalin-2(*H*)-ones with simple ethers, and (**b**) postulated reaction pathway, according to [104]. R^1^ = Me, Et, *n*-Pr, *n*-Bu, Ph, Ph-CH_2_-, and others; R^2^ = H, Me, F, Cl, Br, CN.

Wei et al. [104] developed a facile, efficient, metal-free, visible light-stimulated method for the synthesis of the 3-hydroxyalkylated quinoxaline-2(1*H*)-ones via C—H/C—H cross-dehydrogenative coupling of CH/CH-H quinoxaline-2(*H*)-ones (**12**) with simple ethers (**16**) at room temperature (Figure 7a). The reaction was carried out in the presence of Rose Bengal (RB) (**17**) as a catalyst, DABCO (**18**) as a base, and TBHP (**19**) as an oxidant. Using 3 W blue LEDs was found to be optimal. A reaction mechanism was proposed (Figure 7b).

It is suggested [104] that the initial excited state of Bengal rose (BR*) is obtained using 3 W blue LEDs. Subsequently, as a result of single-electron transfer (SET) between BR* and TBHP (**19**), a hydroxide anion and a tert-butoxy radical are formed. The cleavage of hydrogen from α-C—H tetrahydrofuran (**16**) by tert-butoxy radical then produces the alkoxyalkyl radical intermediate (**16a**). The alkoxyalkyl radical intermediate (**16a**) is then added to (**12**) to give the nitrogen radical intermediate (**12a**). The latter undergoes 1,2-hydrogen shift to deliver carbon radical (**12b**), which is oxidized by BR^•+^ to the carbon cation intermediate (**12c**) via a SET process. Finally, the elimination of β-H from intermediate (**12c**) by a base affords the 3-hydroxyalkylated product (**20**).

**Figure 8:**
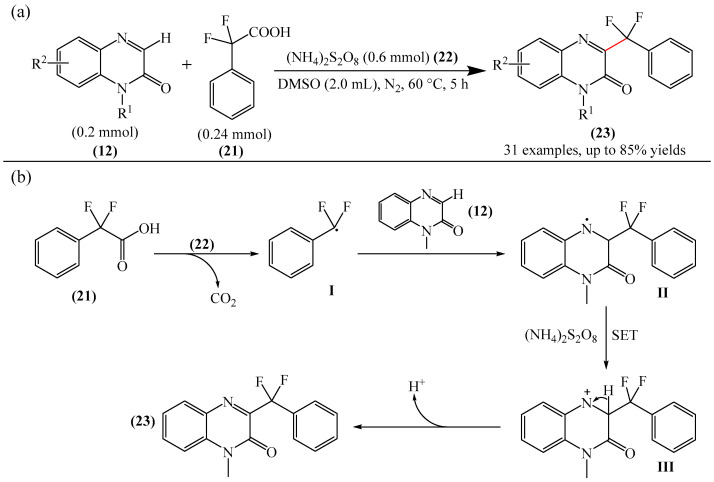
(**a**) Transition metal-free decarboxylative C3 difluoroarylmethylation of quinoxalin-2(1*H*)-ones with α,α-difluoroarylacetic acids, and (**b**) proposed reaction mechanism, according to [105]. R^1^ = H, Me, Et, *n*-Pr, *n*-Bu, C_6_H_4_-CH_2_-, and others; R^2^ = H, Me, F, Cl, Br, NO_2_.

Zhang et al. [105] implemented the decarboxyl radical transition metal-free coupling of the α,α-difluoroarylacetic acids (**21**) with the quinoxaline-2(1*H*)-ones (**12**) (Figure 8a). It is noteworthy that this reaction tolerates a wide range of substrates and proceeds under mild conditions. The only significant drawback is that the most effective strategy requires the use of DMSO as a solvent, since it is extremely difficult, and in some cases impossible, to isolate the key products from DMSO.

It was also assumed [105] that a possible reaction mechanism (Figure 8b) involves radical transformation of difluoroaryl methylation. Specifically, in the presence of (NH_4_)_2_S_2_O_8_ (**22**), α,α-difluorophenylacetic acid (**21**) undergoes decarboxylation to form radical intermediate I, releasing carbon dioxide in the process. Then, intermediate I selectively attacks the C3 position of 1-methylquinoxalin-2(1*H*)-one (**12**) to give nitrogen radical II. Subsequently, single-electron transfer (SET) from II generates intermediate nitrogen cation III in the presence of (NH_4_)_2_S_2_O_8_ (**22**). Finally, deprotonation of III affords the expected product of the oxidative coupling of (**23**). Unfortunately, the authors did not provide any experimental evidence for the existence of the described radical structures, for example, using EPR.

The oxidizing agent (NH_4_)_2_S_2_O_8_ (**22**) was also employed in decarboxylating visible light-induced alkylation of quinoxaline-2(1*H*)-ones (**12**) with carboxylic acids, induced by visible light [106].

**Figure 9:**
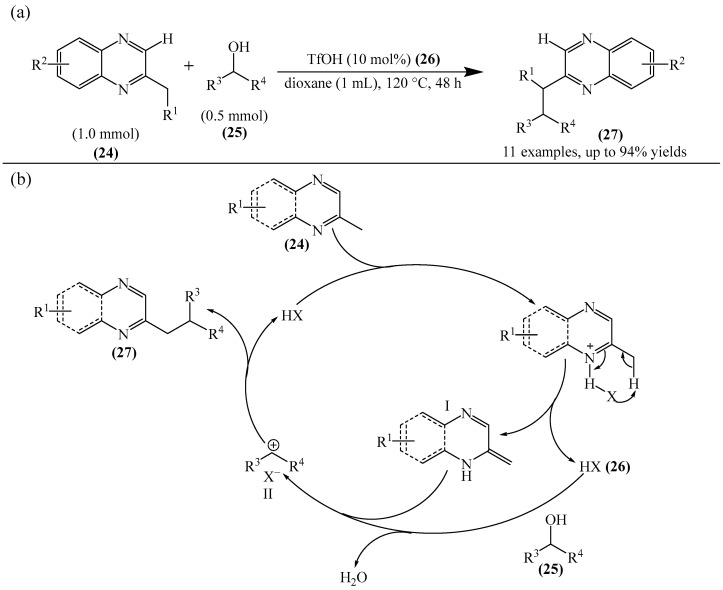
(**a**) Alkylation of *N*-heteroaromatics with alcohols, and (**b**) proposed mechanism for S_N_1-type alkylation of 2-methyl-*N*-heteroaromatics, according to [107]. R^1^ = H or SO_2_ C_6_H_5_; R^2^ = H or NO_2_; R^3^ = C_6_H_5_, 2-FC_6_H_4_, 2-MeC_6_H_4_, 3-MeOC_6_H_4_, 4-MeOC_6_H_4_, and others; R^4^ = ferrocene-functionalized molecules, 4-MeOC_6_H_4_, and others.

The organocatalytic alkylation of 2-methylpyrazines (**24**) with alcohols (**25**) was achieved via S_N_1-type C(sp^3^)−H functionalization [107]. Although this example does not consider the activation of the C–H bond of the heterocycle, but rather the alkylation of the side chain of six-membered polyaza heterocycles, the heterocycle is still directly involved in the proposed mechanism [107]. Accordingly, this example is relevant to the objectives of this review. The trifluoromethanesulfonic acid (TfOH) (**26**) was used as a catalyst (Figure 9a). The proposed mechanism is shown in Figure 9b. TfOH (XH) or (**26**) synergistically catalyzes two catalytic cycles. The first of these involves protonation and activation of the 2-methyl-*N*-heteroaromatic compounds (**27**) to form nucleophilic enamine I. The second cycle comprises the protonation and dehydration of alcohols (**25**) to produce the carbocation intermediate II. The reaction of enamine I=**A** with intermediate II=**B** affords the alkylation product (**27**), with the catalyst being released simultaneously.

**Figure 10:**
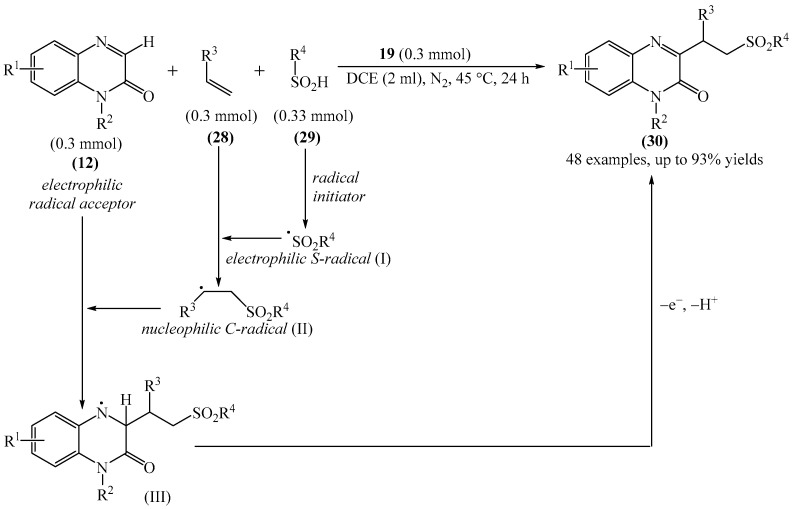
Synthesis of C3 benzyl/alkyl-substituted quinoxalin-2(1*H*)-ones, according to [108]. R^1^ = H, Me, Cl, F, NO_2_; R^2^ = H, Me, Bn, and others; R^3^ = Me, 4-X-C_6_H_4_ (X = Me, OMe, F, Cl, Br, CF_3_, and others), Bn, Cy, and others; R^4^ = H, OMe, C_6_H_5_, Br, I, and others.

Another interesting example of the alkylation of quinoxalin-2(1*H*)-ones (**12**) was described in [108] (see Figure 10). More specifically, this is a metal-free domino multicomponent reaction of direct CH-benzylation and alkylation of quinoxalin-2(1*H*)-ones (**12**) using alkenes (**28**) and alkylsulfinic acid (**29**). Notably, this method not only functionalizes the medically important quinoxalin-2(1*H*)-one skeleton, but also provides a variety of medically important sulfones (**30**) in good to excellent yields under mild conditions.

**Figure 11:**
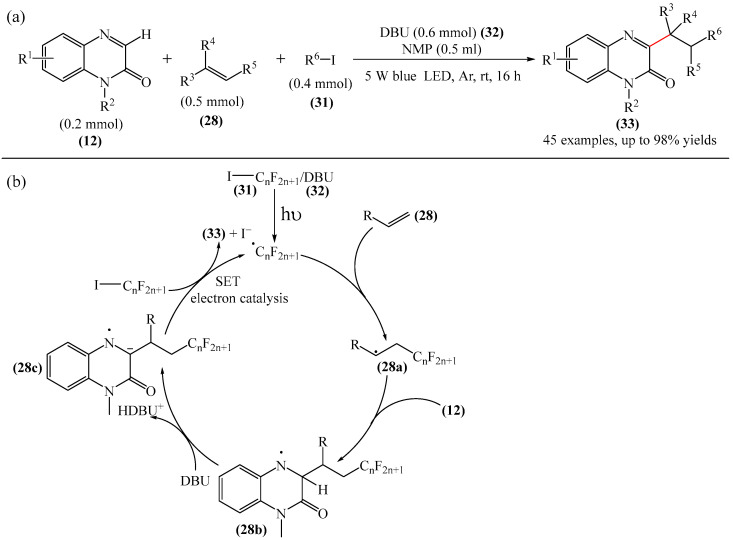
(**a**) A visible light-initiated α-perfluoroalkyl-β-heteroarylation of various alkenes with perfluoroalkyl iodides and quinoxalin-2(1*H*)-ones, and (**b**) proposed mechanism, according to [109]. R^1^ = H, Me, F, Cl; R^2^ = H, Me, C_6_H_5_-CH_2_-, EtCO_2_CH_2_-; R^3^ = R^4^ = R^5^ = C_6_H_5_, C_6_H_5_-CH_2_-, Pr, *i*-Pr, Bu, and others; R^6^ = C_n_F_2n+1_ (n = 1–3, 6, 8).

Studer et al. [109] carried out the efficient mild cascade reaction leading to the formation of a series of model perfluoroalkyl-containing derivatives of quinoxaline-2(1*H*)-one (**12**) in moderate to excellent yields (Figure 11a). The reaction proceeds via acidic amine radicals (**28a–28b**), which can be easily deprotonated to give the corresponding anion radicals (**28c**) that are capable of sustaining the radical chain as one-electron transfer-reducing reagents (Figure 11b). Consequently, the entire cascade can be classified as an electron-catalyzed process.

**Figure 12:**
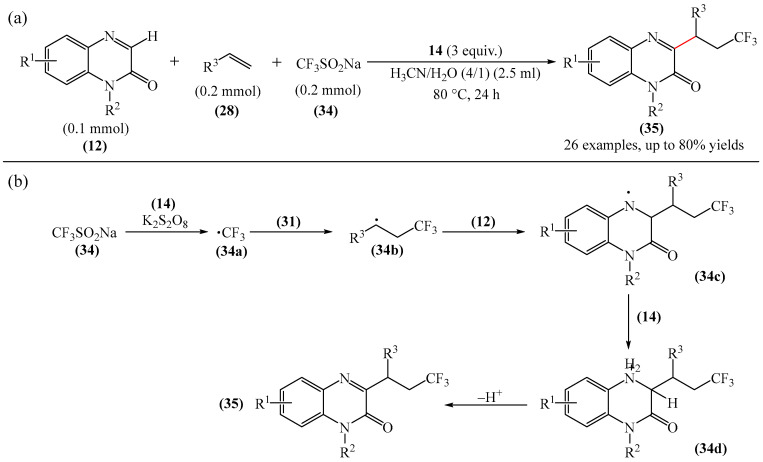
(**a**) Trifluoroalkylation of quinoxalin-2(1*H*)-ones with unactivated alkenes and CF_3_SO_2_Na, and (**b**) proposed mechanism, according to [110]. R^1^ = H, Me, F, Cl; R^2^ = Me, Et, Pr, C_6_H_5_, C_6_H_5_-CH_2_-; R^3^ = C_6_H_5_, 4-MeC_6_H_4_, 2-MeC_6_H_4_, 3,4,5-(Me)_3_C_6_H_2_, and others.

An alternative to the method disclosed in [109] is the approach elaborated in [110]. This involves the trifluoroalkylation of quinoxaline-2(1*H*)-ones (**12**) with unactivated alkenes (**28**) and Langlois reagent (CF_3_SO_2_Na) (**34**), which is an attractive approach for the synthesis of a variety of biologically important 3-trifluoroalkylated quinoxaline-2(1*H*)-ones (**35**) (Figure 12a). In addition to selecting the optimal reaction conditions and substrate set, the reaction mechanism was proposed [110] (Figure 12b). Initially, the CF_3_ radical (**34a**) is generated from CF_3_SO_2_Na using K_2_S_2_O_8_ (**14**). Subsequently, the CF_3_ radical (**34a**) was selectively added to alkene (**28**) to produce alkyl radical (**34b**). Then, the addition of alkyl radical (**34b**) to quinoxaline-2(1*H*)-one (**12**) produces nitrogen radical (**34c**), which is further oxidized with K_2_S_2_O_8_ (**14**) to afford a nitrogen-containing cation intermediate (**37d**). Finally, deprotonation of intermediate (**34d**) delivers the desired product (**35**).

**Figure 13:**
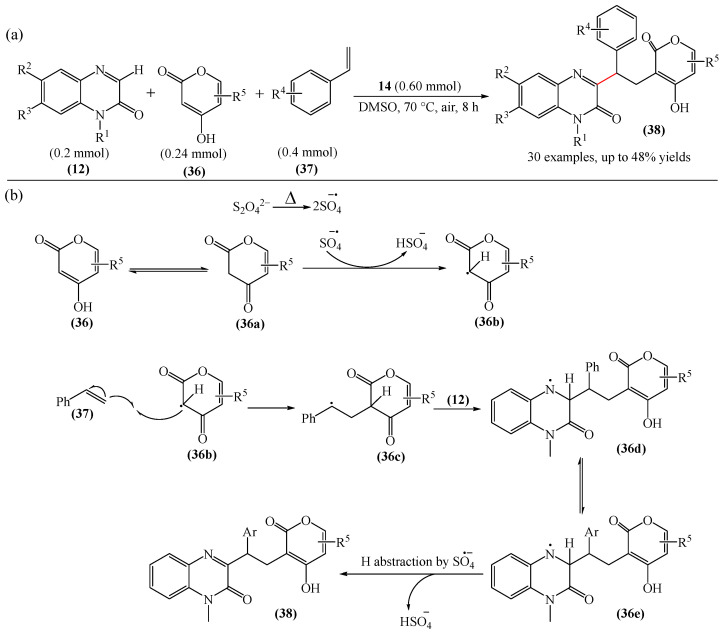
(**a**) Alkylation of quinoxalin-2(1*H*)-ones with vinylarenes and 4-hydroxycoumarins/4-hydroxy-6-methyl-2-pyrone, and (**b**) proposed mechanism, according to [111]. R^1^ = Me, Et, Pr, C_6_H_5_, C_6_H_5_-CH_2_-; R^2^ = R^3^ = H, Me, F, Cl, NO_2_; R^3^ = H, Me, OMe, Cl, Br.

An interesting example of a three-component radical cascade C3-alkylation reaction of quinoxalin-2(1*H*)-ones (**12**) with oxygen-containing heterocyclic compound (**36**) using vinylarenes (**37**) as intermediate radical acceptors was documented in [111] (Figure 13a). The use of K_2_S_2_O_8_ (**14**) in dimethyl sulfoxide (DMSO) was found to be the most efficient oxidant–solvent combination. In addition, a tentative reaction mechanism was proposed (Figure 13b). Initially, 4-hydroxycoumarin (**36**) tautomerizes to (**36a**). Then, the SO_4_^•−^ anion radical formed from K_2_S_2_O_8_ abstracts a H atom from the methylene carbon of (**36a**) to form an intermediate radical (**36b**). This then attacks styrene (**37**) in the terminal position to produce alkyl radical (**36c**). The latter is added to the C3 position of quinoxaline-2(1*H*)-one (**12**) giving an intermediate nitrogen radical (**36d**), which tautomerizes again to (**36e**). Finally, a H atom is cleaved from (**36e**) by the SO_4_^•−^ anion radical present in the reaction medium to afford the target product (**38**).

A transition metal-free alkylation of quinoxaline-2(1*H*)-ones (**12**) in a continuous flow was reported [112]. The reaction was carried out at room temperature, under the action of visible light, using Katritzky salts as alkylating agents, Eosin Y as a photo-oxidative-reductive catalyst and DIPEA (*N,N*-diisopropylethylamine) as a base. The method has several advantages over other approaches, including ease of operation, fast reaction time, mild conditions, and scalability. This makes the method a promising tool for the synthesis of nature-like molecules and various biologically active molecules. However, the authors presented [112] an example of scalability involving 1.12 g of the target product, a quantity that cannot be considered significant, even in the context of laboratory syntheses.

**Figure 14:**
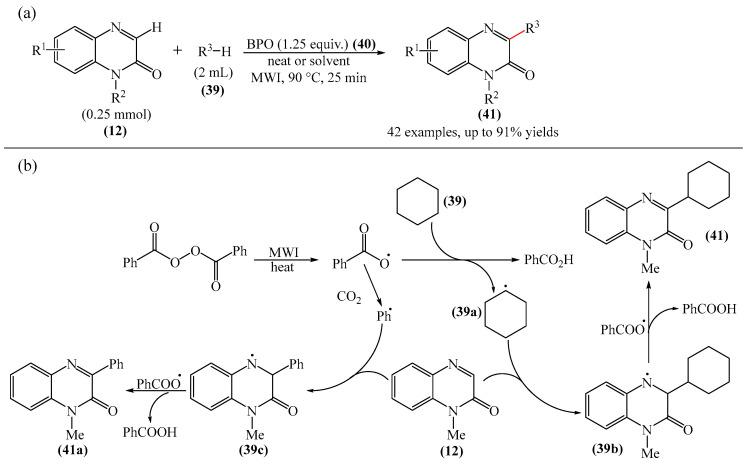
(**a**) Microwave-accelerated alkylation of quinoxalin-2(1*H*)-ones, and (**b**) proposed mechanism, according to [113]. R^1^ = H, Me, Et, C_6_H_5_-CH_2_, CH_2_CO_2_Et, and others; R^2^ = H, Me, OMe, F, Cl, Br, and others; R^3^ = C_x_H_x-1_, where x ≥ 5, and others.

Sun and Zhou et al. [113] presented a fascinating case study involving the use of simple alkanes (**39**), including cyclic alkanes, as precursors of alkyl radicals under microwave irradiation for the alkylation (or cross-dehydrogenative coupling) reaction of quinoxaline-2(1*H*)-ones (**12**) (Figure 14a). The reaction was carried out in organic solvents of different natures, as well as in water, or the reaction was carried out in the absence of solvent(s). Optimal yields were achieved using BPO (**40**) as an oxidizing agent. Based on the experimental results, the authors proposed a mechanism for this reaction (Figure 14b). Initially, BPO (**40**) decomposes into a benzoxy radical under the influence of microwave radiation. Next, a H atom (HAT) of cyclohexane (**39**) is transferred to the benzoxy radical to form the cyclohexyl radical (**39a**). Subsequently, the selective addition of the cyclohexyl radical (**39a**) to the C−3 position of 1-methylquinoxalin-2(1*H*)-one (**12**) produces the nitrogen radical (**39b**). Finally, a hydrogen atom is abstracted from the intermediate compound (**39b**) by another benzoxy radical to give the final product (**41**) and benzoic acid. Based on experimental data, it is established that product (**41a**) is formed in small quantities.

Methods were developed for the alkylation of quinoxaline-2(1*H*)-ones (**12**) in the presence of various photocatalysts, including TPPT (triphenyl phosphorothionate) [114], 4CzIPN (**10**) [82,115,116,117,118], DTBP (di-tertbutyl peroxide) (**42**) [119], PIFA (**2**) [120], Rose Bengal (**17**) [121], 4DPAIPN (1,3-dicyano-2,4,5,6-tetrakis(diphenylamino)-benzene) (**43**) [122], Na_2_S (**44**) [123], anthraquinone-2-sulfonate (**45**) [124], Xanthate A/TT-CF_3_^+^OTf^−^ [76], Mes-Acr-MeClO_4_ (Fukuzumi) (**46**) [125,126], K_2_S_x_-DBU (**47**) [127]. K_2_CO_3_ (**48**) [128], Li_2_CO_3_ (**49**) [78], H_2_O_2_ (**50**) [129], TFA/395 nm purple LEDs [130], and HCl-HNO_3_/10 W LEDs (395 nm) [131] were used as oxidants, while DBU (1,8-diazabicyclo [5.4.0]undeC−7-ene) (**32**) [132], various amines [133] and BBr_3_/HFIP [134] were employed as bases.

Electrolytic methods for the alkylation of the quinoxalinones with electron-deficient alkenes were also documented [135].

Additionally, work [136] was the first to describe photochemical direct C3-cyanoalkylation of quinoxaline-2(1*H*)-ones (**12**) using the cyclobutanone oxime esters as cyanoalkyl precursors, eliminating the need for a photocatalyst.

A very interesting synthetic methodology was developed [137]. In this reaction system, quinoxaline-2(1*H*)-ones (**12**) acted as both a reagent and a photocatalyst. The synthesis was carried out at room temperature for 13 h in the presence of non-activated alkenes (**28**) and IBA-N_3_ (iodonium-based Azide-N_3_), under irradiation with blue LEDs and in a nitrogen atmosphere using DMSO as a solvent. Overall, 22 compounds were obtained in 30-95% yields. Another example of a synthesis in which quinoxaline-2(1*H*)-ones (**12**) act as photocatalysts was described in [138]: the visible light-induced C−3 alkylation of quinoxaline-2(1*H*)-ones (**12**) using the *N,N,N′,N′*-tetraalkyl ethylenediamine, without the need for an external photocatalyst.

In addition, the photochemical catalyst-free alkylation of quinoxaline-2(1*H*)-ones (**12**) using difluoroacetic anhydride and pyridine *N*-oxide [80] or *N,N,N′,N′, N″*-pentamethyldiethylenetriamine (PMDETA) in [139] was reported.

##### 1,2,4-Triazines

**Figure 15:**
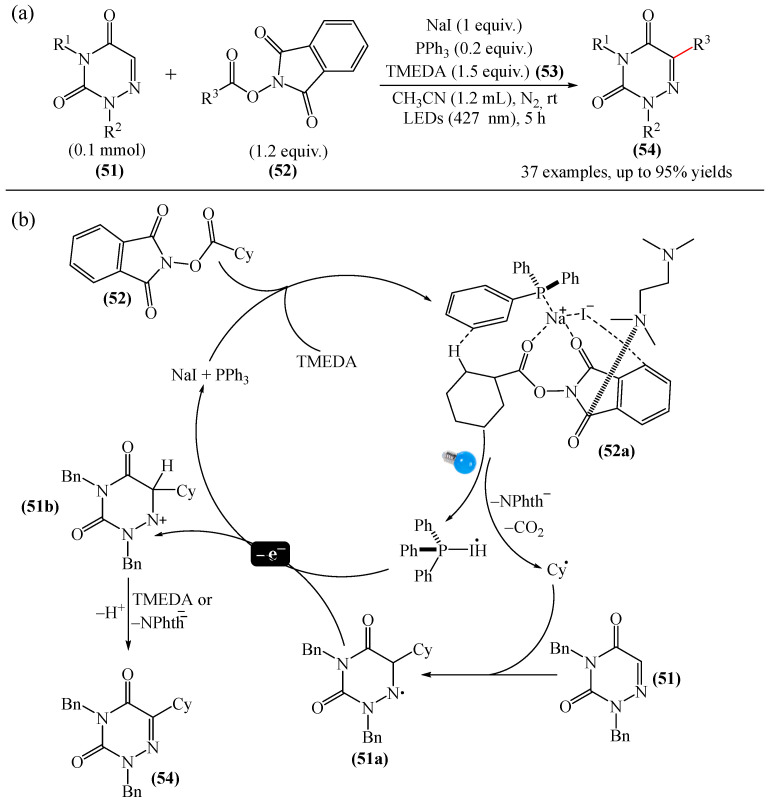
(**a**) C−H alkylation of azauracils with *N*-(acyloxy)phthalimides, (**b**) proposed reaction mechanism, according to [140]. R = *n*-Pr, *n*-Bu, *i*-Bu, and others; R^1^ = Me, Rt, C_6_H_5_, allyl, and others; R^2^ = Bn, allyl, 4-ClC_6_H_4_-CH_2_, and others.

Murarka et al. [140] implemented direct C—H alkylation of azauracils (**51**) using *N*-(acyloxy)phthalimides (NHPI) (**52**) as readily available alkylating agents upon irradiation with visible light (blue LEDs (427 nm)) and in the presence of NaI/PPh_3_ and *N,N,N′,N′*-tetramethylethylenediamine (TMEDA) (**53**) (Figure 15a). It is important to note that this methodology does not require transition metals. It has been shown that under optimal conditions, various azauracils (**51**) undergo alkylation with primary, secondary, and tertiary NHPI esters (**52**) under mild conditions within 5 h, giving the desired products (**54**) in good to excellent yields (up to 95%).

Detailed mechanistic studies have revealed the formation of a photoactivated electron donor–acceptor (EDA) complex between NaI/PPh_3_, TMEDA (**53**), and NHPI alkyl ester (**52**). These studies also established the crucial role of TMEDA in enhancing the activity of the photoredox system (Figure 15b). The reaction demonstrated broad substrate scope, scalability, and significant tolerance to functional groups. Various azauracils were shown to undergo alkylation with primary, secondary, and tertiary NHPI esters under mild conditions to produce the products in good to excellent yields.

**Figure 16:**
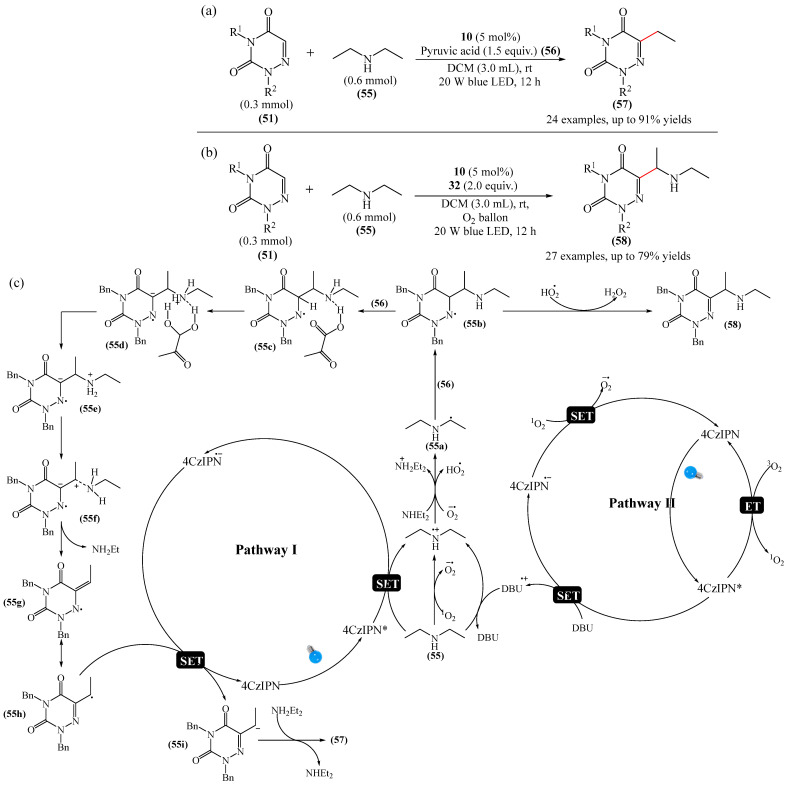
(**a**) Alkylation reaction of 1,2,4-triazine-3,5(2*H*,4*H*)-diones. R^1^ = H, Me, Bn, 4-FC_6_H_4_, 4-MeC_6_H_4_, and others; R^2^ = Me, Bn, C_6_H_5_-CH_2_, C_6_H_5_, CH_2_CH=CH_2_, and others; (**b**) oxidative cross-dehydrogenative coupling reaction of various 1,2,4-triazine-3,5(2*H*,4*H*)-diones with diethylamine under optimal conditions. R^1^ = C_6_H_5_-CH_2_, 4-ClC_6_H_4_, CH_2_CH=CH_2_, and others; R^2^ = C_6_H_5_-CH_2_, 4-ClC_6_H_4_, CH_2_CH=CH_2_, and others; (**c**) proposed reaction mechanism, according to [141]. (Here and further in the text “*” means an excited state).

Zhao et al. disclosed [141] organophotoredox-catalyzed direct C–H alkylation/α-aminoalkylation of 1,2,4-triazine-3,5(2*H*,4*H*)diones (**51**) using inexpensive and readily available secondary amines (**55**) as alkyl sources (Figure 16). The optimal conditions for direct C–H alkylation were the use of pyruvic acid (**56**) as the promoter, 4CzIPN (**10**) as the photocatalyst, and blue light irradiation (20 W) at room temperature (Figure 16a). The oxidative cross-dehydrogenative coupling reaction required 4CzIPN (**10**) as the photocatalyst, DBU (**32**) as a promoter, an O_2_ atmosphere, and blue light irradiation (20 W) at room temperature (Figure 16b). DCM proved to be the best solvent for both products **57** and **58**.

Density functional theory (DFT) calculations demonstrate that the transformation involves a series of elementary steps, including a photo-oxidation–reduction process, pyruvic acid-assisted proton shift, C–N bond cleavage, and tautomerization (Figure 16c).

**Figure 17:**
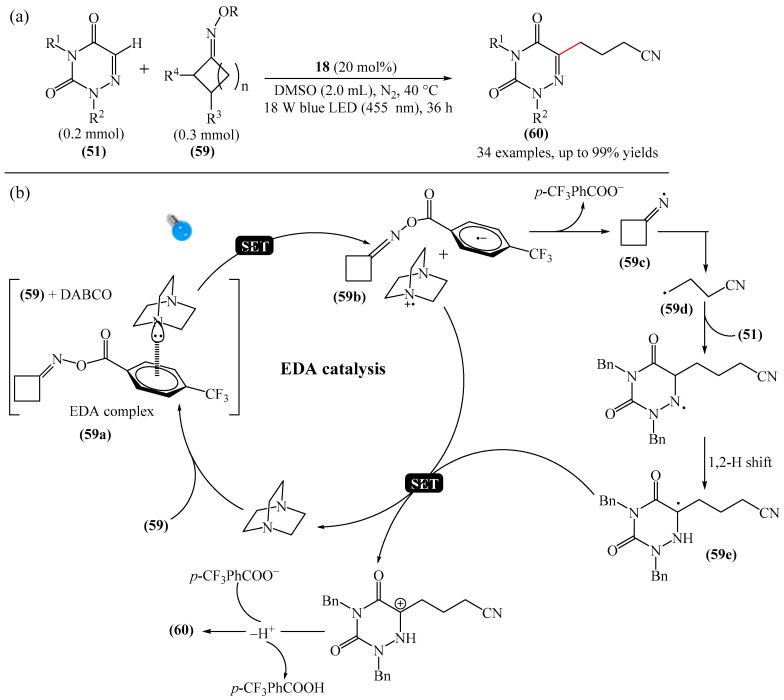
(**a**) Visible light-induced C−H cyanoalkylation of azauracils with cycloketone oxime esters, and (**b**) proposed mechanism, according to [142]. R = *p*-CF_3_C_6_H_4_CO; R^1^ = R^2^ = Me, Et, Bn, allyl, C_6_H_5_, and others; R^3^ = R^4^ = C_6_H_5_, Boc, CO_2_Et, and others.

Another example of photoinduced C–H cyanoalkylation of azuracil (**51**) cycloketone oxime esters (**59**) by photoexcitation electron donor–acceptor complexes (Figure 17a), using inexpensive and readily available 1,4-diazabicyclo [2.2.2]octane (DABCO) (**18**) as a catalytic electron donor was described [142]. A significant disadvantage of this method, particularly compared with the previous one, is the excessively long reaction time and using DMSO as a solvent.

A tentative mechanism of the reaction was proposed (Figure 17b). Initially, DABCO (**18**) interacts with the cyclobutanone oxime ester (**59**) to produce an electron donor–acceptor complex (**59a**). Upon irradiation, a single-electron transfer occurs, forming DABCO^+•^ and an anion radical (**59b**). Subsequently, the anion radical (**59b**) undergoes N-O bond cleavage to deliver imine radical (**59c**) and an acid ion. The imine radical (**59c**) promotes ring opening to generate an intermediate cyanoalkyl radical (**59d**). The latter is radically added to the C–N bond, affording a radical intermediate (**59e**). Finally, compound (**59e**) is oxidized by DABCO^+•^ to give a carbocation, which is subsequently deprotonated in the presence of acid ions to form the desired product (**60**).

**Figure 18:**
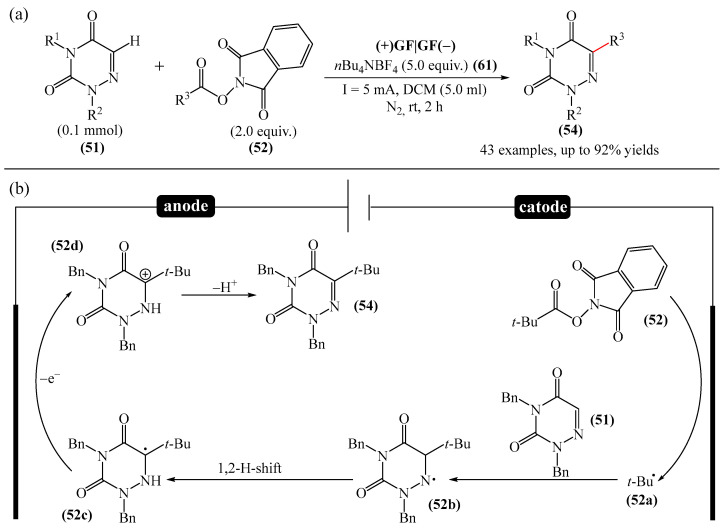
(**a**) Electrochemical C−H alkylation of azauracils using *N*-(acyloxy)phthalimides, (**b**) proposed mechanism, according to [143]. R^1^ = H, Me, Et, Bn, allyl, Br, and others; R^2^ = Bn, and others; R^3^ = alkyl, *i*-alkyl, *t*-alkyl, and others.

The electrochemical C—H alkylation of azuraciles (**51**) using *N*-(acyloxy)phthalimides (NHPI esters) (**52**) as readily available alkyl radical precursors was developed [143]. The protocol eliminates the need for expensive catalysts containing transition metals, ligands, or any additives (Figure 18a). However, the authors of the article were somewhat disingenuous, since, judging by the description of the experimental procedures, *n*-Bu_4_NBF_4_ (**61**) acts precisely as a specially introduced additive. This methodology can be implemented in both batch and flow modes, making it suitable for various industrial applications.

Mechanistically, the reaction was rationalized as follows [143] (Figure 18b). Initially, the NHPI ester (**52**) undergoes cathodic reduction to form the alkyl radical (**52a**) with the simultaneous removal of CO_2_ and the phthalimide anion. Subsequently, the alkyl radical (**52a**) is added to the iminic carbon of azuracil to produce *N*-centered radical (**52b**). The latter, upon 1,2-H shift, yields carbon-centered radical (**52c**). Finally, anodic oxidation of the carbon-centered radical (**52c**) followed by deprotonation give the target product (**54**).

**Figure 19:**
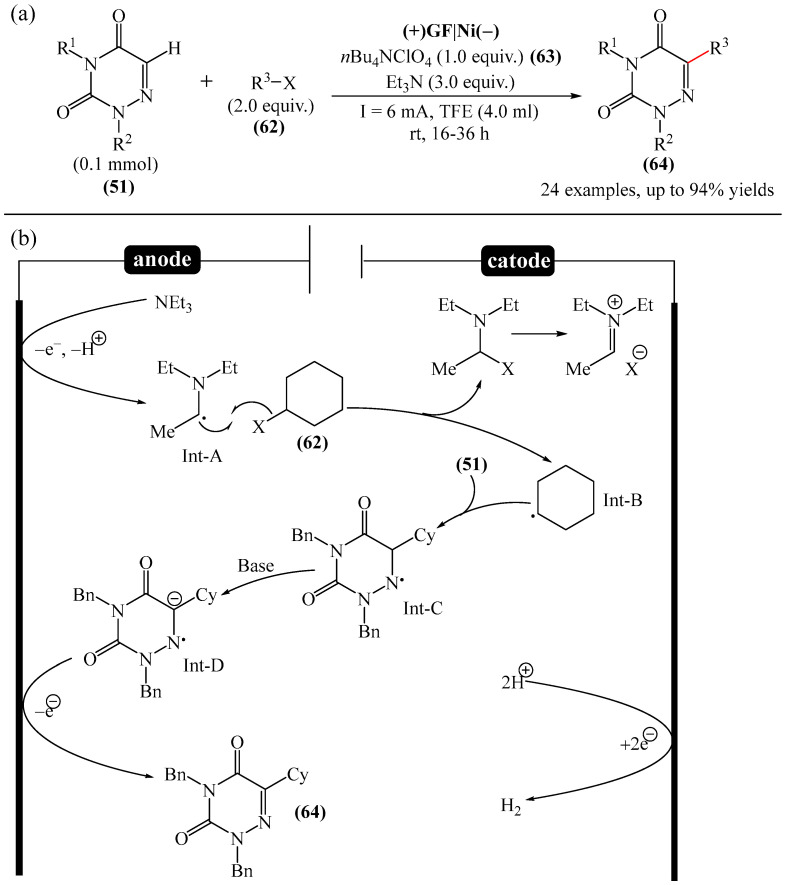
(**a**) Electrochemical C–H functionalization of *N*-heterocycles with 1,2,4-triazine moety/fragment, and (**b**) proposed mechanism, according to [144]. R^1^ = H, *n*-Bu, and others; R^2^ = Bn, C_6_H_5_, and others; R^3^ = C_6_H_5_, *n*-Bu, *i*-Pr, and others; X = Br, I.

The electrocatalyzed alkylation of heterocyclic compounds containing a 1,2,4-triazine fragment **51** with alkyl halide (**62**) using halogen atom transfer (HAT) agents was realized [144]. The optimal conditions for the synthesis included the use of *n*-Bu_4_NClO_4_ (**63**) as the electrolyte, NEt_3_ as the additive, and trifluoroethanol as the solvent, with an electric current of 6 mA (Figure 19a). Mechanistically (Figure 19b), the reaction proceeds via the initial electrochemical oxidation of triethylamine, followed by deprotonation to the intermediate α-aminoradical Int-A. This intermediate then abstracts a halogen atom from the alkyl halide to form the alkyl radical Int-B and α-halogenamine. Finally, the intermediate compound Int-B is added to the imine carbon followed by deprotonation and oxidation to produce **64**.

**Figure 20:**
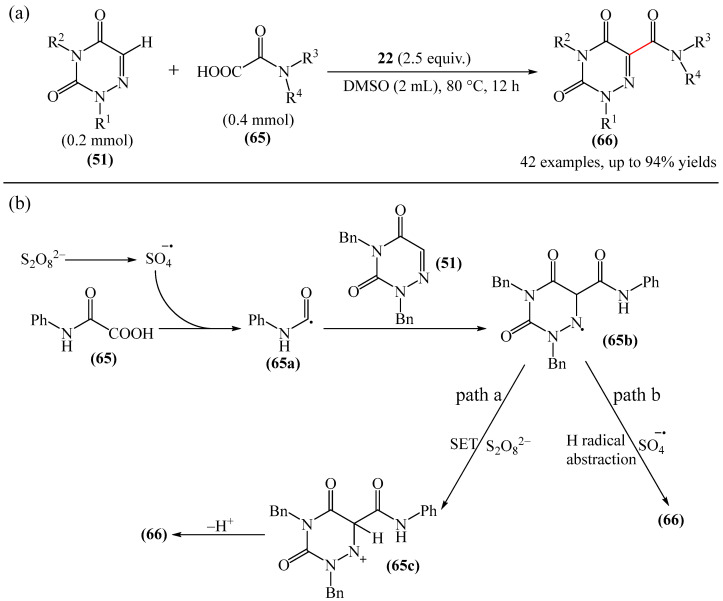
(**a**) Minisci-type carbamoylation of azauracils with oxamic acids, and (**b**) proposed mechanism, according to [145]. R^1^ = Me, Et, Bn, 4-MeC_6_C_4_, 4-BrC_6_C_4_, and others; R^2^ = Bn, 4-BrC_6_C_4_, EtOOC, and others; R^3^ = C_6_H_5_, 4-MeC_6_C_4_, 4-EtC_6_C_4_, 4-*t*-BuC_6_C_4_, and others; R^4^ = H, Me, and others.

Pan and Yu et al. [145] developed a metal-free method for the direct radical C6-carbamoylation of azauracils (**51**) and oxamic acids (**65**) to afford a series of 6-carbamoylazauracils (**66**) (42 examples) in yields of 52–94% (Figure 20a). However, this reaction had the significant disadvantage of requiring (NH_4_)_2_S_4_O_8_ (**22**) as an oxidizing agent and dimethyl sulfoxide (DMSO) as a solvent.

A plausible mechanism of the process was proposed [145] (Figure 20b). Initially, a sulfate anion radical SO_4_^−·^ is formed via the homolytic cleavage of the peroxydisulfate dianion. Then, SO_4_^−·^ radical react with 2-oxo-2(phenylamino)acetic acid (**65**) to generate a carbamoyl radical (**65a**) through hydrogen atom transfer (HAT) and decarboxylation processes. Next, the carbamoyl radical (**65a**) is selectively added to the C6 position of azuracil (**51**), producing nitrogen-centered radical (**65b**). The latter undergoes single-electron transfer to give a nitrogen-centered cation (**65c**), which delivers product **66** after deprotonation (pathway a). Alternatively, product **66** can be obtained through the abstraction of radical H by SO_4_^−·^ (pathway b). In view of the pharmacological potential of 6-carbamoyluracil fragments **66**, this protocol offers a greater number of opportunities to promote their structural diversity for the discovery and development of new drugs.

**Figure 21:**
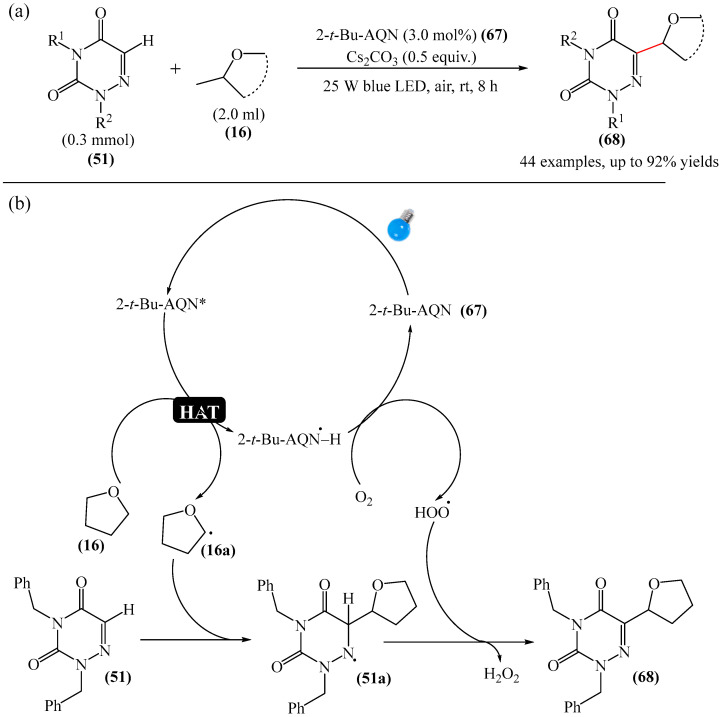
(**a**) Visible light-induced oxyalkylation of 1,2,4-triazine-3,5(2*H*,4*H*)-diones with ethers, and (**b**) plausible mechanism, according to [146]. R^1^ = R^2^ = H, Me, *n*-Pr, CH_2_-CH=CH_2_, and others.

An efficient and straightforward method for the synthesis of 6-hydroxyalkylated 1,2,4-triazine-3,5(2*H*, 4*H*)-diones (**68**) was reported [146]. The method comprised a visible light-initiated dehydrogenative cross-coupling reaction of 1,2,4-triazine-3,5(2*H*,4*H*)-diones (**51**) with ethers (**16**), which tolerated a wide variety of functional groups (Figure 21a). The reaction occurred in the presence of the inexpensive, low-toxicity 2-*tert*-butylanthraquinone (**67**) as a metal-free photocatalyst, and air as a ‘green’ oxidant, at room temperature. Furthermore, this reaction can be initiated using sunlight as an environmentally benign energy source. The only drawback of the method is the use of relatively expensive Cs_2_CO_3_ as a base. A possible reaction mechanism is presented in Figure 21b.

Initially, 2-*t*-Bu-AQN* is generated, when 2-*t*-Bu-AQN (**67**) is irradiated with blue light. Next, a hydrogen atom transfer process between THF (**16**) and 2-*t*-BuAQN* gives an α-hydroxy radical A and a 2-*t*-BuAQN^•^-H structure. Then, 2-*t*-Bu-AQN is formed through the oxidation of 2-*t*-Bu-AQN^•^-H affording a hydroperoxyl radical HO_2_^•^. In this process, the α-hydroxy radical (**16a**) attacks **51** to produce the radical intermediate (**51a**), which then reacts with the HO_2_^•^ radical to deliver H_2_O_2_ and the final product **68**.

The synthetic utility of the method [146] was further demonstrated by gram-scale reactions, as well as by its application in preparing key intermediates of bioactive molecules.

##### 1,3,5-Triazines

**Figure 22:**
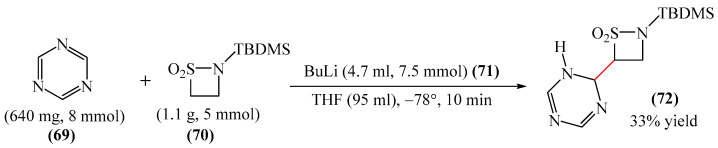
Reaction of triazine with (*t*-butyldimethylsilyl)-thiazetidine-dioxide, according to [147]. TBDMS = (*i*-Bu)Me_2_Si.

**Figure 23:**
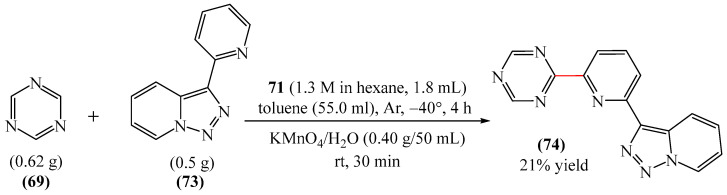
Reaction of triazine with 3-(2-pyridyl)triazolopyridine, according to [148].

A method for the synthesis of [(*t*-butyl)dimethylsilyl](dihydro-triazinyl)-thiazetidine-dioxide (**72**) via the reaction of 1,3,5-triazine (**69**) with (tert-butyldimethylsilyl)thiazetidine-dioxide (**70**) in the presence of *n*-butyllithium (**71**) in THF was explained [147] (Figure 22). Despite the fact that heterocycle **72** has one fewer unsaturated bonds than heterocycle **69**, the reaction presented in Figure 23 deserves attention since there are few model examples of C–H functionalization of 1,3,5-triazine rings.

Abarca et al. [148] obtained 3-[6-(1,3,5-triazin-2-yl)pyridin-2-yl]-[1,2,3]triazolo [1,5-*a*]pyridine (**74**) in a yield of 21% through the addition of 7-thio-3-(2-pyridyl)triazolopyridine (**73**) to 1,3,5-triazine (**69**), followed by oxidation (treatment with an aqueous solution of KMnO_4_ for 30 min at room temperature) (Figure 23).

**Figure 24:**
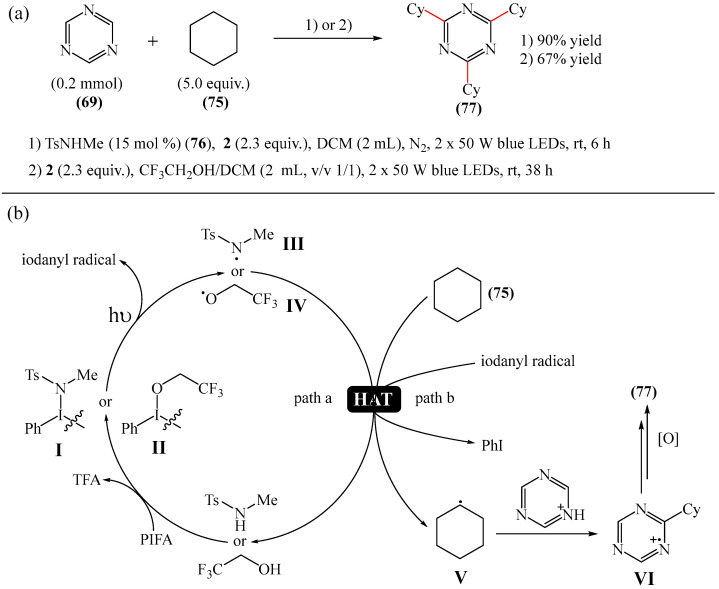
(**a**) Interaction of triazine with dicyclohexylmethane, and (**b**) proposed mechanism, according to [149].

A metal-free, N/O-centered radical-promoted Minisci reaction under mild neutral conditions was carried out [149]. No additional acid is required to preactivate the 1,3,5-triazine (**69**), and N/O-centered radicals are formed directly from the amide (TsNHMe) (**76**) or the alcohol (CF_3_CH_2_OH) in the presence of visible light (Figure 24a).

Mechanistically, the reaction [149] can be rationalized as depicted in Figure 24b. The process is triggered by the interaction between PIFA (**2**) and amide/alcohol to generate intermediate I/II. This then leads to the corresponding amidyl/alkoxy radical (III or IV) after homolysis under the action of visible light. Meanwhile, an iodanyl radical is also formed. The N-/O-centered radical abstracts a H atom from alkane (**75**) to give radical V (path a). Alternatively, the iodanyl radical may participate in another possible HAT process (path b). It should be noted that the TFA formed in situ preactivates the *N*-heteroarene (**69**), thus avoiding the need for additional acids to be added. Subsequently, the nucleophilic addition of the alkyl radical V to the acidified heteroarene produces the intermediate compound VI, which is oxidized to form the final product **77**.

#### 2.1.2. Metal-Catalyzed Alkylation

##### Pyridazines

**Figure 25:**
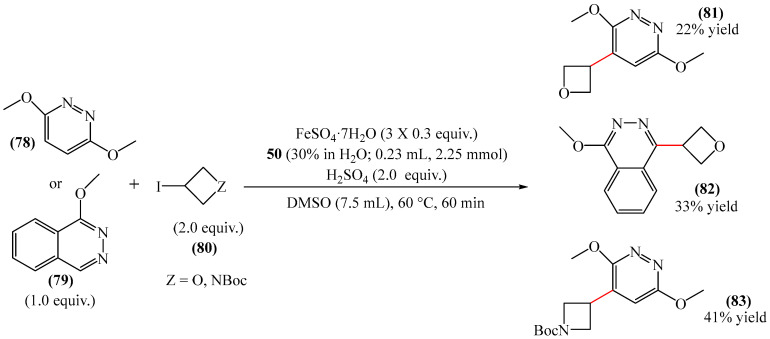
Iron-mediated Minisci-type alkylation of pyridine derivatives with heterocyclic alkyl iodides, according to [70].

A method for the alkylation of pyridazine fragments was disclosed [70] (Figure 25). The process involved the incorporation of oxetane (or azetidine groups) into heteroaromatic bases **78** and **79** using a radical approach (the Minisci reaction). The optimal conditions for this process were the use of the H_2_O_2_–FeSO_4_ system in DMSO in the presence of H_2_SO_4_ as the acidic component. Unfortunately, the authors did not provide any information about the mechanism of this process. Moreover, the utilization of DMSO as a solvent constitutes a substantial drawback of this methodology.

**Figure 26:**
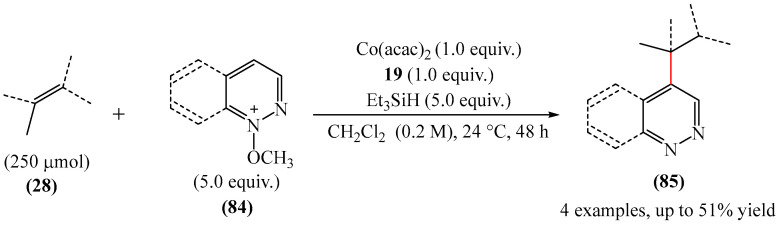
Hydroheteroarylation with *N*-methoxypyridiazinium, according to [150].

A protocol for the alkylation of pyridazinium derivatives (**84**) in the presence of a catalytic system based on Co(acac)_2_ and Et_3_SiH, and *tert*-butylhydroperoxide (TBHP) (**19**) was proposed [150] (Figure 26). The synthesis was carried out in dichloromethane at room temperature for 48 h.

**Figure 27:**
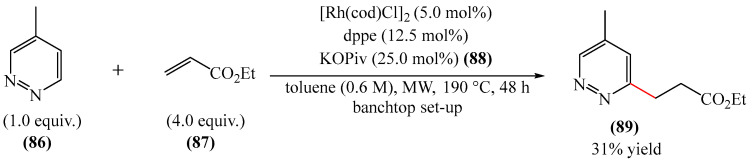
Rhenium-mediated alkylation of 4-methylpyridazine, according to [151].

Ellman et al. [151] described the alkylation of a pyridazine fragment (**86**) in the presence of a catalytic system based on a rhenium complex [Rh(cod)Cl]_2_ and a dppe ligand, as well as potassium pivaltate (KOPiv) (**88**). The alkylation was performed using microwave heating at 190 °C in toluene (Figure 27). Notwithstanding the apparent simplicity of the method, its implementation requires a tabletop microwave oven, a factor which hinders its widespread adoption. Furthermore, the authors did not make any mention of the potential scaling of the method.

**Figure 28:**
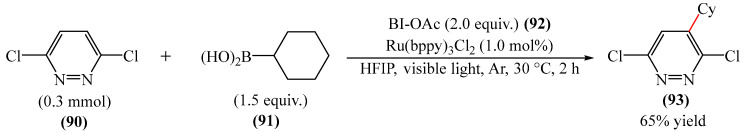
Minisci C–H alkylation of 3,6-dichloropyridazine, according to [152].

Liu and Chen et al. [152] implemented a photoredox-mediated C–H alkylation Minisci reaction of the pyridazine cycle (**90**) under the action of the (1,2-dicyclohexylethyl)boronic acid (**91**) (Figure 28). The reaction was carried out in an argon atmosphere at 30 °C, using Ru(bpy)_3_Cl_2_ as the catalyst, the acetoxybenziodoxole (BI-OAc) (**92**) as then oxidant and hexafluoroisopropanol (HFIP) as the solvent. Visible light was provided by a compact household fluorescent bulb (20 W). Unfortunately, the reaction was performed on a 0.2 mmol scale, with no mention of the possibility of scaling it up.

It was suggested [152] that acetoxybenzoxole (**92**) serves as an easy precursor to the *ortho*-iodobenzyloxy radical intermediate under photo-oxidative–reductive catalytic conditions. The unique property of this intramolecularly stabilized benzoxy radical may be crucial for the efficient conversion of typically less reactive alkylboronic acids into alkyl radicals.

**Figure 29:**
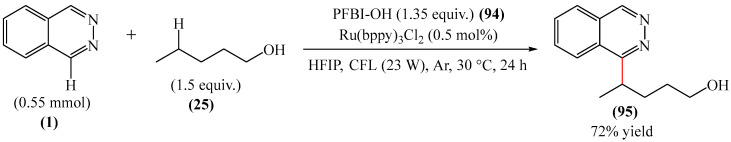
Minisci C–H alkylation of phthalazine, according to [153].

Another example of Minisci’s photoredox catalytic C–H alkylation in the presence of hypervalent iodine-containing oxidant (**94**) was reported by Chen et al. [153] (Figure 29). The reaction was carried out under irradiation with a 23 W compact fluorescent lamp (CFL) at 30 °C for 24 h, using Ru(bpy)_3_Cl_2_ as the catalyst, perfluorinated hydroxybenziodoxole (PFBI-OH) as the oxidant, and hexafluoroisopropanol (HFIP) as the solvent.

According to [153], the reaction sequence includes mild alcoholysis of PFBI-OH (**94**) with alcohol (**25**) in situ, the generation of an intermediate alkoxy radical by SET-reduction, 1,5-HAT, and the formation of a Minisci-type C–C bond. Despite the relatively high yields reported in [152] and [153], both methods have a significant disadvantage in that they require the use of the Ru(bpy)_3_Cl_2_ complex as a catalyst, as well as the rather expensive solvent hexafluoroisopropanol.

**Figure 30:**
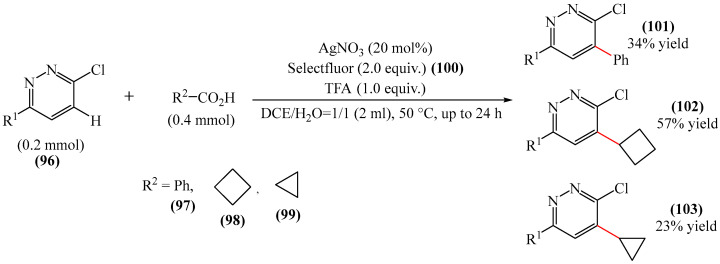
Silver-catalyzed Minisci reactions using Selectfluor as a mild oxidant, according to [154].

Baxter et al. [154] presented a silver-catalyzed Minisci reaction using Selectfluor (**100**) as a mild oxidant (Figure 30). The best yields were achieved using 20 mol% AgNO_3_, 2.0 equiv. (0.4 mmol) of the corresponding carboxylic acid **97–99**, 2.0 equiv. of Selectfluor (**100**), 1.0 equiv. of trifluoroacetic acid, and a mixture of DCE/H_2_O (0.1 M) in a 1:1 ratio as the solvent. The synthesis was carried out at 50 °C for 24 h. A major drawback of this method is the long reaction time and the use of Selectfluor (**100**) as an oxidant. The latter is expensive, which can make scaling up the synthesis more complicated.

**Figure 31:**
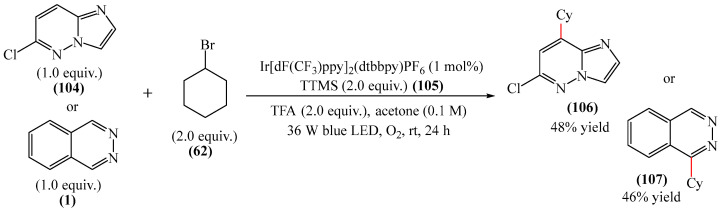
Visible light-mediated Minisci C–H alkylation of pyridazine moiety with unactivated alkyl halides using O_2_ as an oxidant, according to [155].

Wang et al. [155] achieved the direct room temperature visible light-mediated Minisci C–H alkylation of pyridazine moiety (**1** and **104**) with unactivated alkyl halides (**62**) using molecular oxygen as an oxidant. Phthalazine (**107**, 46%) and 6-chloroimidazo [1,2-*b*]nypyrazine (**106**, 48%) were examined as model molecules (Figure 31). The latter can only formally be considered a molecule with a pyridazine fragment since one of the nitrogen atoms is attached to the imidazole ring. Although the reported yields are not high, they are indicative and enable previously impossible transformations to be realized. Phthalazine is an important pharmacophore for creating drugs with a wide range of biological activities, including anticancer, antihypertensive and anti-inflammatory. It is also used in developing polymeric materials. 6-Chlorimidazo [1,2-*b*]pyridazine is commonly used as a building block in pharmaceutical research, agrochemicals, and materials science due to its unique imidazopyridazine core.

##### Pyrimidines

One of the most rapidly developing methods for the alkylation of heteroarenes containing nitrogen atoms in the 1,3-position is visible light-enabled radical C(sp)^2^–H alkylation. Review [156] is entirely devoted to this topic and contains the most interesting examples published before 2019. Therefore, this paper covers the works published later.

**Figure 32:**
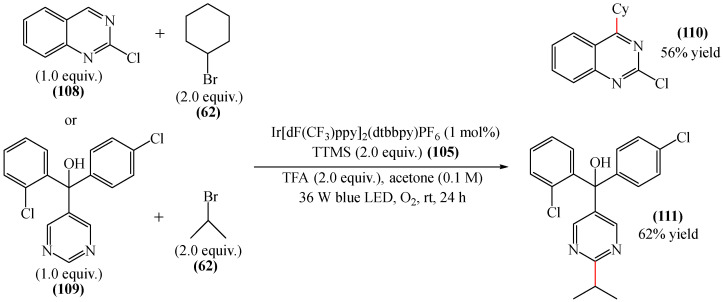
Visible light-mediated Minisci C–H alkylation of pyrimidine moiety with unactivated alkyl halides using O_2_ as an oxidant, according to [155].

Reference [155] also provided examples of the alkylation of molecules containing a pyrimidine fragment, namely 2-chloroquinazoline (**110**, 56%) and fenarimol (**111**, 62%) (Figure 32). The yields of the products were higher than those achieved for molecules with pyridazine fragments. Despite there being only two model examples, fenarimol (**111**) is a systemic fungicide from the pyrimidine group that is used to combat fungal diseases such as powdery mildew, rust and black spot on ornamental plants, fruit trees (such as apple and pear) and vegetables (such as cucumbers and tomatoes). It inhibits ergosterol biosynthesis in fungi. 2-Chloroquinazoline (**110**) is also an important building block in organic synthesis, and is employed in the design of pharmaceuticals, pesticides, and functional materials.

##### Pyrazines

**Figure 33:**
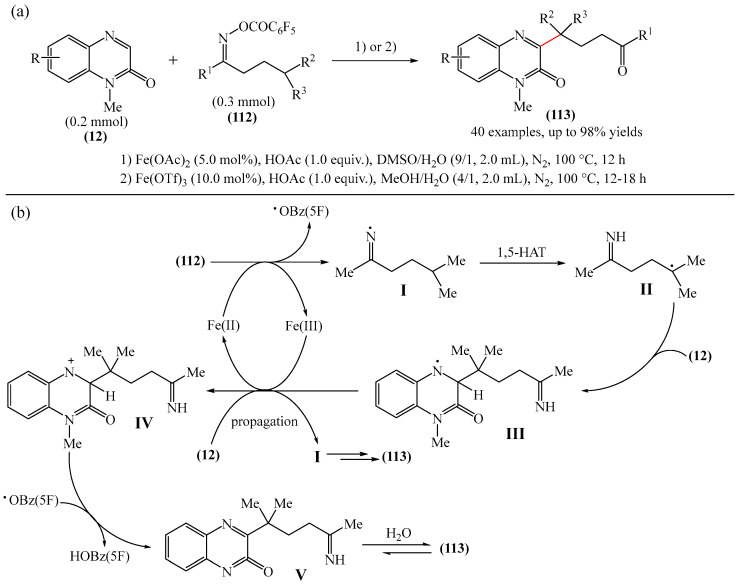
(**a**) Iron-catalyzed intermolecular distal interaction C(sp^3^)−H quinoxalin-2(*H*)-ones with alkyl ketones, and (**b**) proposed mechanism, according to [157]. R = H, Me, OMe, F, Cl, Br, NO_2_, and others; R^1^ = H, Me, and others; R^2^ = R^3^ = H, Me, C_6_H_5_, and others.

Another effective and regioselective method for functionalization of heteroarenes was detailed [157] (Figure 33a). The reaction was carried out at 100 °C for 12 h using 5 mol% Fe(OAc)_2_ as the catalyst and 1.0 equiv. HOAc as the additive in DMSO/H_2_O (9:1, by volume). Other iron(II)- and iron(III)-based catalysts, such as FeCl_2_, Fe(OTf)_2_, and Fe(OTf)_3_, exhibited lower catalytic activity. Conversely, Pd(OAc)_2_, Co(OAc)_2_·4H_2_O, Ni(OAc)_2_·7H_2_O and Cu(OAc)_2_ were less effective or completely ineffective in this reaction compared to iron-based catalysts.

Based on data on the composition of the starting reagents and products, as well as literature data, a tentative mechanistic rationale of the reaction was proposed (Figure 33b). The reaction begins with the one-electron reduction of Fe(II) oxime ester (**112**) to afford the corresponding iminyl radical I, which triggers the 1,5-HAT (hydrogen atom transfer) process involving the formation of carbon-centered radical II. The latter is then captured by substrate **12** to give radical III, which is oxidized by Fe(III) and subsequently deprotonated to deliver precursor of product **V**. Finally, the hydrolysis of compound **V** furnishes the desired product **116**. However, the mechanism of radical chain development, whereby the oxidation of radical intermediate III by **112** produces product **113**, cannot be completely ruled out.

**Figure 34:**
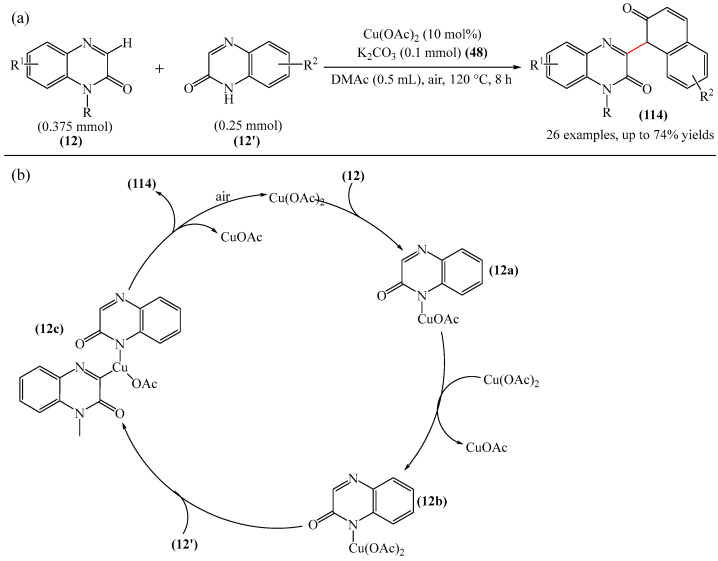
(**a**) Copper-catalyzed C–H/N–H cross-coupling reactions for the synthesis of 3-heteroaryl quinoxalin-2(1*H*)-ones, and (**b**) proposed reaction mechanism, according to [158]. R = Me, *i*Pr, 4-butenyl; R^1^ = H, Me, F, Cl, Br, NO_2_; R^2^ = C_6_H_5_, F- C_6_H_4_, and others.

From a synthetic point of view, a very interesting reaction was documented in [158]. This is the efficient copper-catalyzed direct C–H/N–H cross-coupling of quinoxaline-2(1*H*)-ones (**12**) with various unprotected 2-quinoxalinones and 2-quinolinones (**12′**) (Figure 34a). Furthermore, the target products have been identified as promising candidates for further study as potent anticancer drugs with significant antitumor activity against gastric cancer. Mechanistically, the process is initiated by coordination of 2-quinoxalinone (**12**) with Cu(OAc)_2_ to form the Cu(II) intermediate (**12a**) (Figure 34b). The latter subsequently disproportionates with another Cu(OAc)_2_ molecule producing the Cu(III) intermediate (**12b**) and CuOAc. Then, metalation–deprotonation of the sp^2^ C–H (**12**) bond, promoted by (**12b**), delivers a biheteroaryl copper (III) structure (**12c**). Finally, reductive cleavage of (**12c**) generates the C(sp^2^)–N **114** cross-coupling product together with the Cu(I) center, which is then reoxidized to Cu(II) for the next reaction cycle.

Another example of the alkylation of quinoxalin-2(1*H*)-ones was described in [159], in which CuCN was used as a catalyst and DTBP (di-tert-butyl peroxide) (**42**) as an oxidant. In Reference [160], a CuI and TBHP (**19**) system was employed, which was formed in the presence of 2,2′:6′,2″-terpyridine. It was also reported [161] on the alkylation of quinoxalin-2(1*H*)-ones in the presence of CuCl as a catalyst and *tert*-butyl peroxybenzoate (TBPB) (**115**) as an oxidant.

**Figure 35:**
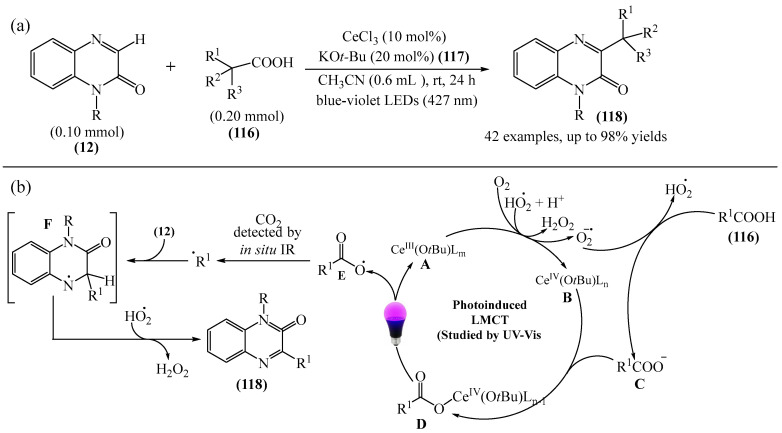
(**a**) Cerium-mediated decarboxylative alkylation of quinoxalin-2(1*H*)-ones, and (**b**) proposed reaction mechanism, according to [159]. R = H, Me, 4-butenyl, and others; R^1^ = H, Me, Et, C_6_H_5_, and others; R^2^ = H, Me, Et, and others; R^3^ = H, C_6_H_5_, and others.

In addition to transition metals, the alkylation of quinoxalin-2(1*H*)-ones (**12**) can be carried out in the presence of cerium salts. For instance, decarboxylating alkylation of quinoxalin-2(1*H*)-ones via photoinduced ligand-to-metal charge transfer (LMCT) using starting carboxylic acids as radical precursors was described in [162] (Figure 35a).

A plausible reaction mechanism was also proposed (Figure 35b). Initially, the Ce^III^ complex (A) undergoes aerobic oxidation to form the Ce^IV^ complex (B) and an oxygen anion radical (O_2_^−•^) (E_1/2_(O_2_/O_2_^−•^) = 0.56 B vs. SCE). Although the aerobic oxidation of Ce^III^ is thermodynamically unfavorable, the authors [162] suggested, based on literature studies, that the oxide-rich Ce^III^ complex and its associated anionic ligands would promote the aerobic oxidation of Ce^III^ by stabilizing the higher oxidation state of Ce^IV^. Furthermore, O_2_^−•^ (or KO*t*Bu (**117**)) abstracts a proton from the carboxylic acids, thereby generating a HO_2_^•^ radical and a carboxylate ion (C), which coordinates with the complex to form Ce^IV^ (D). After being irradiated with light, complex D undergoes a photoinduced LMCT process to produce a carboxyl radical (E) and regenerate the Ce^III^ catalyst. Next, E participates in rapid decarboxylation to form a key alkyl radical, which is added to **12** to afford an intermediate compound (F), which instantly yields the desired alkylated products **118** upon elimination of a hydrogen atom.

The method for alkylation of quinoxalin-2(1*H*)-ones in the presence of cesium salt, Cs_2_CO_3_, under the action of 18 W 395 nm purple LEDs was also reported in [163].

The photocatalytic formation of C−C bonds between quinoxalin-2(1*H*)-ones and pentacoordinated phosphoran as a substrate was promoted by an iridium complex [164]. Meanwhile, the work [165] described a method for the direct C(sp^2^)–H fluoroalkylation of quinoxalin-2(1*H*)-ones with (fluoroalkyl)triphenylphosphonium salts and alkenes in the presence of *faC−*Ir(ppy)_3_. An efficient method for the synthesis of various 3-difluoroalkylquinoxaline-2(1*H*)-ones via a photoredox-catalyzed three-component reaction between quinoxaline-2(1*H*)-ones and vinyl arenes using inexpensive and readily available difluoromethyltriphenylphosphonium bromide salt as a difluoromethylated reagent and *faC−*Ir(ppy)_3_ as a catalyst was developed [166]. Singh et al. [59] implemented a Pd-catalyzed photochemical reductive alkylation of quinoxaline-2(1*H*)-ones and dibenzoxazepines with readily available alkyl bromides.

##### 1,2,4-Triazines

**Figure 36:**
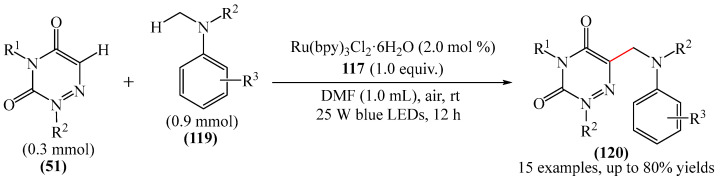
Visible light-induced C(sp^2^)−C(sp^3^) cross-dehydrogenative-coupling reaction of 1,2,4-triazines with *N*-alkyl-*N*-methylanilines, according to [167]. R^1^ = Me, CH_2_-C_6_H_5_, CH_2_-C_6_H_4_-4F, and others; R^2^ = H, Me, Et, *n*-Bu, and others; R^3^ = H, Me, OMe, Et, and others.

Zhao and Zhang [167] carried out the interaction between1,2,4-triazine-3,5(2*H*, 4*H*)-diones (**51**) and *N*-methylanilines (**119**) to form a new C(sp^2^)-C(sp^3^) bond (Figure 36). The reaction proceeded at room temperature in ambient air under the action of visible light. In this case, air acted as a “green” oxidant. This method is highly atom-economic and environmentally benign. It is easy to use, and provides effective access to aminomethyl-substituted heterocycles (**120**) with broad functional group compatibility in yields of up to 80%. The only significant drawback from the perspective of completely “green” chemistry is the use of the transition metal complex Ru(bpy)_3_Cl_2_·6H_2_O as a catalyst.

##### 1,3,5-Triazines

**Figure 37:**
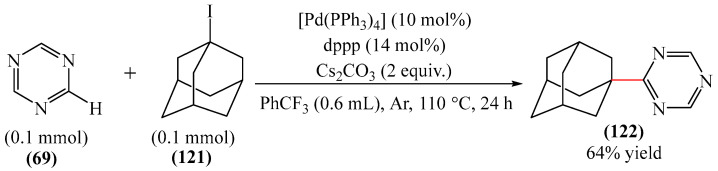
Couplings of 1-adamantyl iodide and 1,3,5-triazine, according to [56].

Zhou et al. [56] described a method for the alkylation of 1,3,5-triazine (**69**) using 1-adamantyl iodide (**121**) as an alkylating agent (Figure 37). The reaction occurred in the presence of the system [Pd(PPh_3_)_4_]/diphosphine ligand dppp as a catalyst, Cs_2_CO_3_ as a base, and PhCF_3_ as a solvent. The authors also employed dry PhCF_3_, which was pre-degassed by bubbling with argon and stored above activated 4 Å molecular sieve beads in an argon-filled glove box.

This chapter turned out to be quite extensive. This is primarily due to the practical needs of pharmaceutical chemistry and the requirements for synthesizing specific molecules, since most of the methodologies described focus on creating pharmaceutically active molecules or structures with biological activity. The most illustrative examples have therefore been summarized in Table 1. The data presented allow us to draw several general conclusions: (1) The nature and yields of the target products depend not only on the nature of the heterocycles, but also on the nature of the substrate and the conditions under which the alkylation processes are carried out; (2) high yields of target products can be achieved not only in the presence of transition metal complexes (lines 13–20), but also under metal-free conditions (lines 1–12); and (3) the examples described in the literature do not yet enable us to create a general theory of the functionalization of polyaza six-membered heterocycles. However, they do enable us to create some model structures based on the methodologies already described.

### 2.2. Arylation Reactions

#### 2.2.1. Metal-Free Arylation

##### Pyridazines

**Figure 38:**
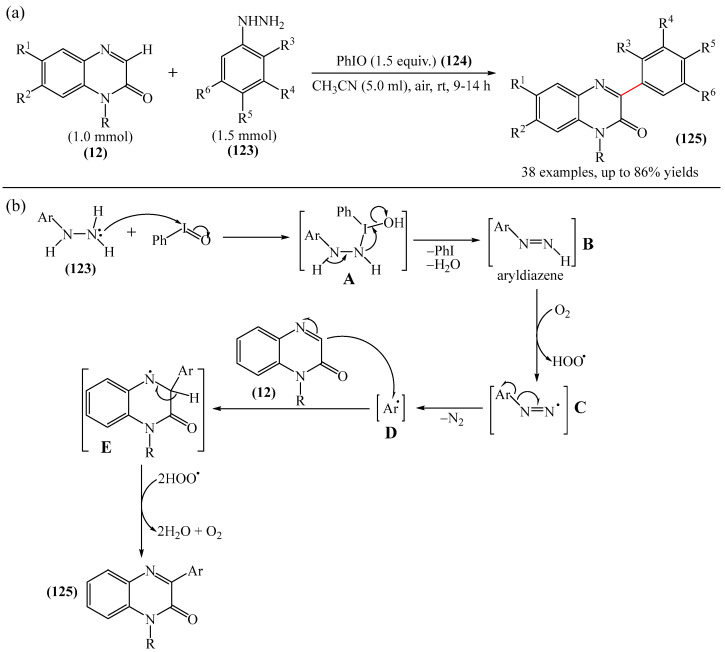
(**a**) PhIO-promoted synthesis of various 3-arylquinoxalin-2-ones, and (**b**) proposed mechanism for the formation of 3-arylquinoxalin-2(*H*)-one, according to [168]. R = H, Me, allyl, geranyl, and others; R^1^ = H, Me, F, Cl, and others; R^2^ = H, Me, F, Cl, and others; R^3^ = H, Me; R^4^ = H, Me; R^5^ = H, Me, OMe, *i*-Pr, Cl, F, CN, and others; R^6^ = H, Me.

It was reported [168] on the PhIO-mediated direct oxidative arylation of quinoxaline-2(*H*)-ones (**12**) with arylhydrazines (**123**) to afford a variety of 3-arylquinoxaline-2(*H*)-ones (**125**) (see Figure 38a). The reaction involves PhIO-mediated cleavage of N–H and C–N bonds of arylhydrazines followed by direct cross-coupling with various quinoxaline-2(*H*)-ones (**12**) (Figure 38b). A significant advantage of this method is its high tolerance to base-sensitive ester groups, moderately strong electron-withdrawing groups such as -F, -CF_3_, and -CO_2_Me, as well as highly sensitive *N*-benzyl- and allyl-substituted quinoxalin-2(*H*)-ones. This direct arylation reaction was used to synthesize biologically interesting compounds such as 3-arylbenzo[*g*]quinoxalinones and 3-arylpyrido [3,4-*b*] pyrazinones.

A tentative reaction mechanism is shown in Figure 38b. First, arylhydrazine (**123**) reacts with PhIO (**124**) to form an intermediate compound **A**. The latter, after the elimination of water and PhI, produces aryl diazine (**B**). Subsequently, intermediate B is rapidly oxidized by aerobic oxidation to give a diazenyl radical **C,** which then transforms into an aryl radical **D** by releasing molecular nitrogen. Intermediate products **B** and **C** were induced from arylhydrazine by oxidation in air. The reaction of aryl radical **D** with quinoxaline-2(*H*)-one (**12**) delivers intermediate compound **E**, which is ultimately oxidized by hydroperoxy radicals generated during the formation of **C**, resulting in the final product **125**. In the final step, the removed hydrogen radical is added to the hydroperoxy radical to furnish water and oxygen. In the course of the reaction, the pH of the medium remains unchanged because no acid or base is formed that could interfere with it.

**Figure 39:**
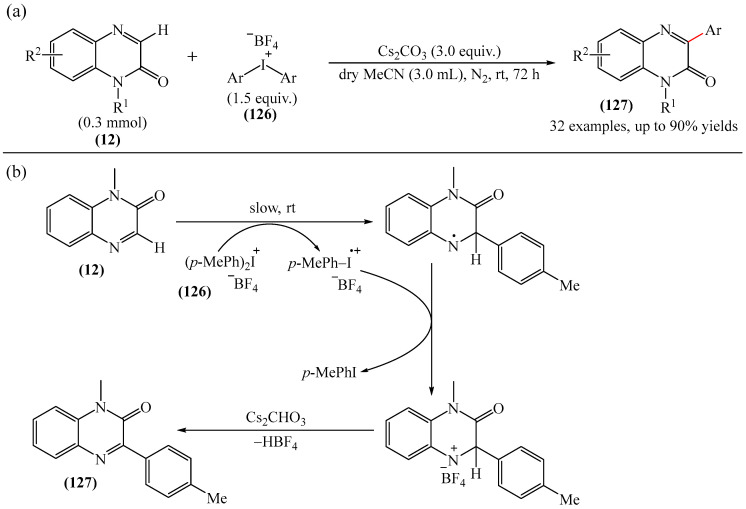
(**a**) Direct C−H arylation of quinoxalin-2(1*H*)-ones with diaryliodonium salts, and (**b**) proposed mechanism for arylation of quinoline-2(1*H*)-ones, according to [169]. R^1^ = Cl, Br, F, NO_2_, Me, OMe, CF_3_, and others; R^2^ = H, F, Cl, NO_2_, Me, OMe, C_6_H_5_, and others; Ar = C_6_H_5_, 4-X-C_6_H_4_ (X = F, Cl, Br, Me, OMe, CF_3_), and others.

3-Arylquinoxalin-2(1*H*)-ones (**127**) were synthesized from diaryliodonium tetrafluoroborates (**126**) under mild conditions [169]. It was demonstrated that the reaction was carried out in the absence of transition metal catalysts. However, to achieve optimal yields, Cs_2_CO_3_ (3.0 equiv), dry solvents, and an inert atmosphere were used (Figure 39a), which makes the described method less accessible. A mechanism of the reaction was also presented (Figure 39b) although no physicochemical evidence was provided to support its feasibility.

Dihydroquinoxalin-2-ones can undergo arylation in the presence of photocatalysts such as 4CzIPN (**10**) [77], *t*-BuOK (**117**) [170], Eosin Y (**128**) [171].

**Figure 40:**
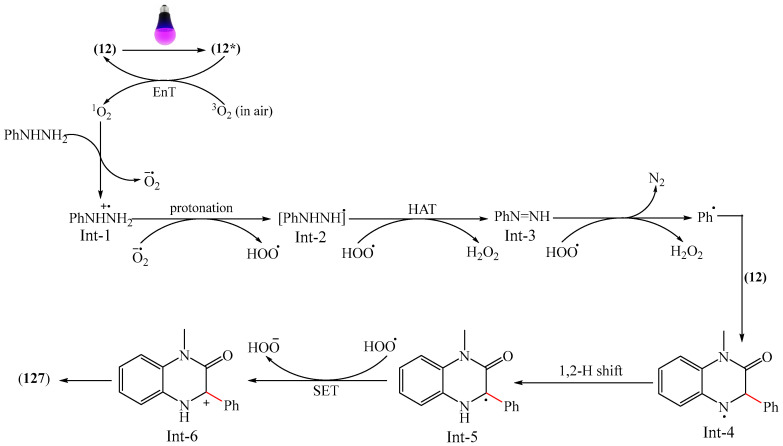
Proposed mechanism self-catalyzed sonophotocatalyzed arylation of quinoxaline-2(1*H*)-ones, according to [172].

The first example of self-catalyzed sonophotocatalyzed arylation of quinoxaline-2(1*H*)-ones using phenylhydrazines was disclosed in [172]. The reaction was carried out under the synergistic combination of 4 W purple-light LED irradiation and 44 kHz/30 W ultrasound in the presence of MeCN solvent in air without any photocatalyst and exogenous additive for 4 h. Thirty compounds were synthesized in 64–77% yields. The proposed mechanism is shown in Figure 40.

When irradiated with a violet LED, the substrates (**12**) act as photosensitizers, absorbing photons to reach an excited state 12a*. This is followed by an energy transfer (ET) process involving triplet oxygen (^3^O_2_) in the ground state (in air) to form highly excited singlet oxygen (^1^O_2_), which reduces the ground state 1a. ^1^O_2_ absorbed one electron from phenylhydrazines to produce an intermediate compound Int-1 due to single-electron transfer, which then reacted with a superoxide radical anion (O_2_^·−^), delivering the radical Int-2. As a result, Int-2 was attacked by a peroxide radical (HO_2_^·^) to afford an intermediate compound Int-3 by releasing a H_2_O_2_ molecule (detected using an H_2_O_2_ test strip). Phenyl was formed by the release of N_2_ and then regioselectively attacked the C–N bond of quinoxaline-2(1*H*)-one (**12**), forming another intermediate product, Int-4. The 1,2-H-shift stage promoted the conversion of Int-4 to Int-5. Int-5 then underwent single-electron oxidation with the more active HO_2_ group to produce the cationic intermediate Int-6, whose subsequent dehydrogenation/rearomatization gave the target products (**127**).

A visible light-induced C(sp^2^)H arylation of quinoxaline-2(1*H*)-ones was developed [79]. The reaction proceeds at room temperature for 10 h using iodonium ylides, K_2_CO_3_ (**51**) as a base in DMSO, and 6 W LED (410 nm), and does not require external photocatalysts. Overall, 28 products were obtained in yields of 57–85%.

##### 1,2,4-Triazines

**Figure 41:**
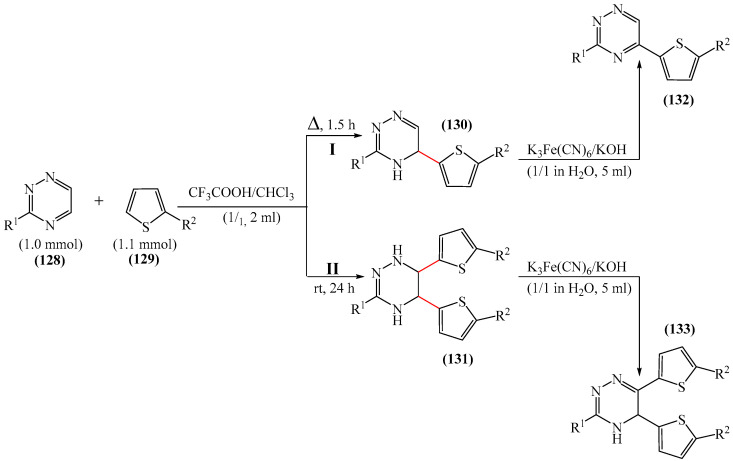
C—H functionalization of 1,2,4-triazines, according to [173]. R^1^ = SMe, NH_2_, R^2^ = H, Br, C_6_H_5_, and others.

A method for the arylation of 1,2,4-triazine derivatives (**128**) using furyl structures (**129**) in the presence of the oxidation mixture K_3_(Fe(CN)_6_)—KOH was documented [173] (Figure 41). It was established that in the first stage of the reaction, an adduct of thiophene to the triazine ring was formed **130** and **131**. In some cases, the σH adducts were sufficiently stable to be isolated in their free form. The stability of the σH adducts depends on the nature of the substituent in the thiophene ring. The greater the donor effect of the substituent (e.g., phenyl), the more stable the addition product and consequently the higher the yield of the adduct. By contrast, it was not possible to isolate either the corresponding adduct or the product of nucleophilic aromatic hydrogen substitution in the case of thienyl-2-carbaldehyde.

The second stage involves the aromatization of adducts. In this case, the oxidizing agent can be applied to either the reaction mixture (without isolating the intermediate compound) or to individual dihydrotriazines, provided they are stable. The structure of the synthesized compounds was confirmed by a combination of spectroscopic methods and X-ray structural studies using product thiols (5-(2,2′-bithiophen-5-yl)-3-methylsulfanyl-1,2,4 triazine according to [173]) as an example.

#### 2.2.2. Metal-Catalyzed Arylation

##### Pyrimidines

**Figure 42:**
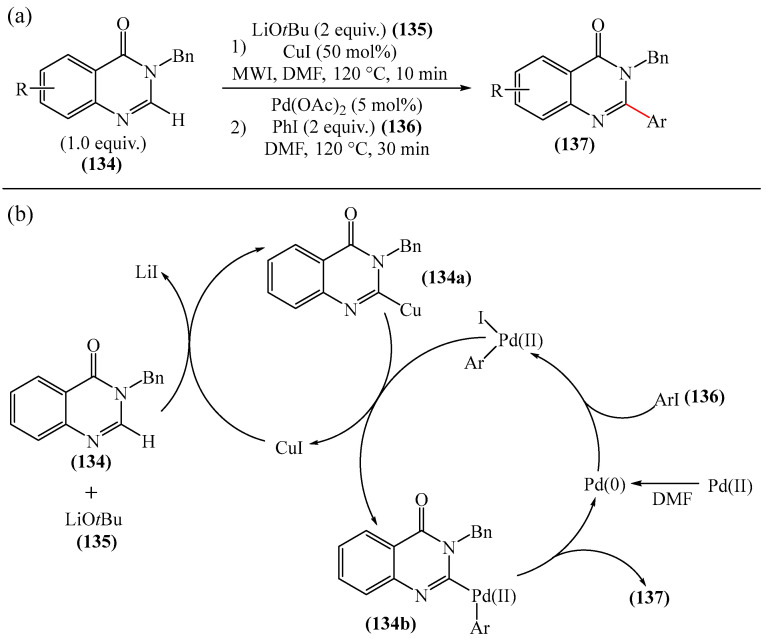
(**a**) Direct arylation of N3-benzylated quinazolin-4-one with aryl iodides, and (**b**) plausible mechanism for the C−2 arylation of quinazolin-4-one, according to [174]. R = 4-MeOC_6_H_4_-, 4- C_6_H_5_OC_6_H_4_-, 4-MeC_6_H_4_-, 4-nBuC_6_H_4_-, 4-BrC_6_H_4_, 4-ClC_6_H_4_-, 4-FC_6_H_4_-, 4-CNC_6_H_4_, 4-CF_3_C_6_H_4_, 4-NOC_6_H_4_, 3-MeC_6_H_4_, 3-MeOC_6_H_4_, 2-MeOC_6_H_4_, 2-MeC_6_H_4_, 1-naphthyl, 2-naphthyl, 9-phenanthryl, 3-pyridyl; Ar = C_6_H_5_, 4-MeOC_6_H_4_, 4-ClC_6_H_4_-, and others.

The direct C—H (hetero)arylation of quinazolin-4-ones (**134**) with aryliodes (**136**) was achieved [174]. The reaction occurred in the presence of a ligand-free Cu/Pd catalyst under microwave irradiation and tolerated a variety of aryliodes (**136**) and substituted (2*H*)-quinazolin-4-ones (**134**). A plausible mechanism of the arylation [174] is shown in Figure 42a.

ESI-MS/MS data demonstrate that, during the activation (**136**) equivalent of CuI interacts with LiO*t*Bu (**135**) and quinazolinone (**134**) to form a cation, [(Cu(**134**)) + H]^+^ (*m*/*z* 299) (Figure 42b), which exhibits the same fragmentation in MS/MS as product **137** itself (*m*/*z* 91). Based on these results, it was suggested that the exchange of the most acidic proton at C−2 with Cu leads to the formation of an organocopper intermediate. The latter can undergo a cross-coupling reaction with iodopalladium compounds to afford the C−2 arylated product **137**.

##### Pyrazines

**Figure 43:**
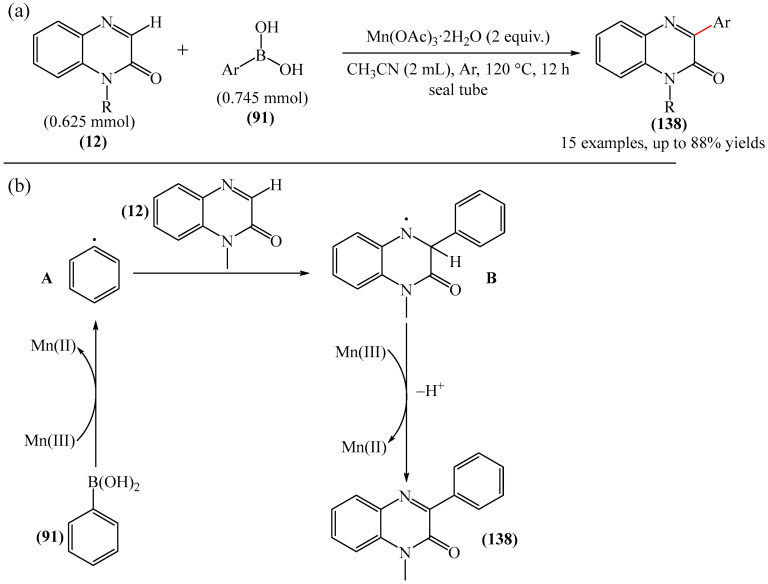
(**a**) Addition of arylboronic acids on cyclic imines, and (**b**) plausible reaction mechanism, according to [73]. R = Me, CH_2_-C_6_H_5_; Ar = C_6_H_5_, 4-MeC_6_H_4_, 4-NO_2_C_6_H_4_, 4-CF_3_C_6_H_4_, and others.

An interesting example of arylation was described in [73], where Mn(OAc)_3_-2H_2_O was employed as an oxidant in the synthesis of 3-arylquinoxalin-2-ones (**138**) (Figure 43a). Mechanistically, the reaction was suggested to proceed via the formation of aryl radical A from arylboronic acid (**91**) (Figure 43b). Subsequent addition of this to -CH=N- produces the nitrogen radical B, and further generation of a radical using Mn(OAc)_3_-2H_2_O affords the desired product **138**. A significant disadvantage of this method is that it requires a long reaction time and an inert atmosphere to achieve good conversion. However, it does not require precious metal-based catalysts, which is undoubtedly an advantage.

Arylation of pyrazines can also be carried out in the presence of palladium [53,60,71,175] or ruthenium compounds [176], Pt electrodes [177], as well as ionic liquids based on 1,3-bis(4-sulfobutyl)-1*H*-imidazol-3-ium [84].

**Figure 44:**
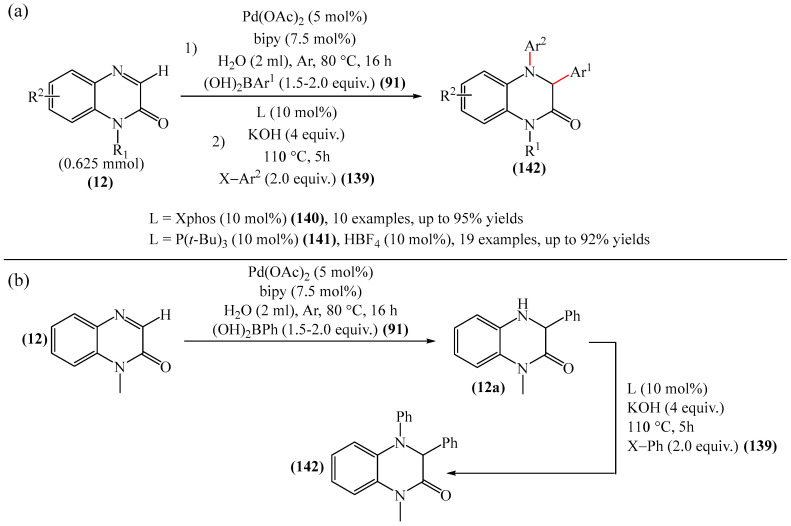
(**a**) Coupling reaction of quinoxalin-2(1*H*)-one a with PhB(OH)_2_, and (**b**) plausible reaction mechanism of Pd(II)-catalyzed C3-arylation and Pd(0)-catalyzed N4-arylation through an assisted tandem process using a reductive trigger, according to [178]. X = F, Cl, Br; R^1^ = H, Me, CH_2_-C_6_H_5_, CH_2_-CO_2_Et, and others; R^2^ = H, F; Ar^1^ = C_6_H_5_, MeC_6_H_4_, MeOC_6_H_4_, FC_6_H_4_, and others; Ar^2^ = C_6_H_5_, MeOC_6_H_4_, NH_2_C_6_H_4_, FC_6_H_4_, and others.

Work [178] describes an efficient and straightforward methodology for metal-catalyzed heteroarylation for palladium(II)- and palladium(0)-catalyzed direct C−3 and *N*-4 arylation (only benzene-based aryl groups) of quinoxalin-2(1*H*)-one (**12**) with boronic acids (**91**) and aryl halides (**139**) in an aqueous reaction medium [178]. The authors explored a broad range of substrates, providing an efficient route to a diverse array of biologically valuable 3-aryl-4-aryl(alkenyl)-dihydroquinoxalin-2(1*H*)-ones (**142**) (Figure 44a). A plausible reaction mechanism was also proposed (Figure 44b).

### 2.3. Acylation Reactions

#### 2.3.1. Metal-Catalyzed Heteroarylation

##### Pyrazines

**Figure 45:**
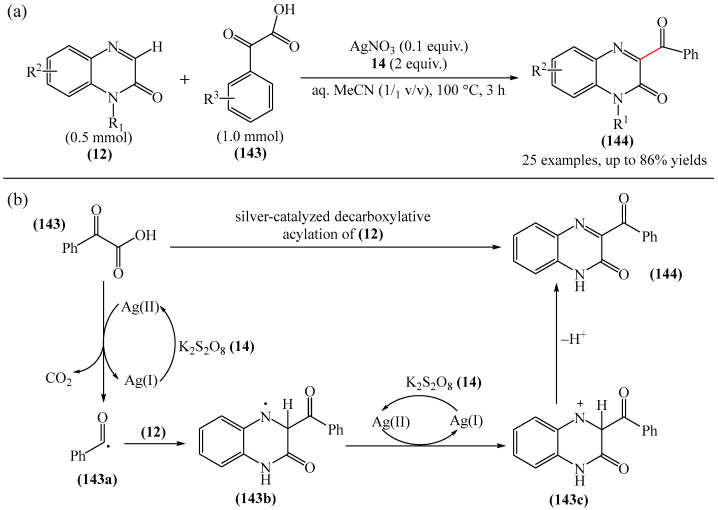
(**a**) Silver-catalyzed decarboxylative acylation of quinoxalin-2(1*H*)-ones with *α*-oxo-carboxylic acids, and (**b**) proposed reaction pathway, according to [51]. R^1^ = H, Me, C_6_H_5_-CH_2_-, -CH_2_CO_2_Et; R^2^ = Me, Cl, Br; R^3^ = C_6_H_5_, 2-MeC_6_H_4_-, 3-MeC_6_H_4_-, 4-MeC_6_H_4_-, 2-X-C_6_H_4_- (X = OMe, Cl, Br, H) and others.

Work [51] describes the synthesis of 3-acylquinoxalin-2(1*H*)-ones (**143**) via silver-catalyzed direct acylation of quinoxalin-2(1*H*)-ones (**12**) (Figure 45a).

A possible reaction mechanism was proposed (Figure 45b). First, AgNO_3_ is oxidized by K_2_S_2_O_8_ (**14**) to form an Ag(II) compound, which abstracts a single electron from (**143a**), generating an acyl radical (**143a**) via decarboxylation. This radical is then captured by (**12**) to produce intermediate (**143b**), resulting in the formation of a new C–C bond. Finally, intermediate (**143b**) undergoes deprotonation to afford the target product **144**. However, the authors did not provide any evidence for this mechanism.

##### 1,3,5-Triazines

**Figure 46:**
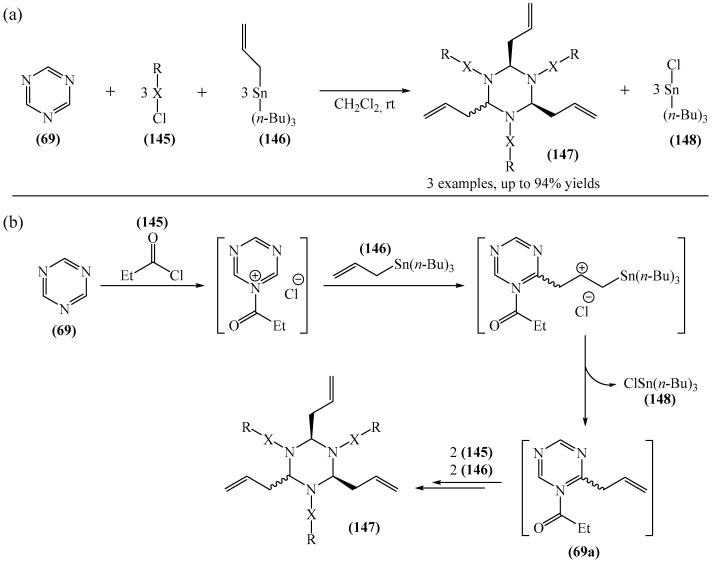
(**a**) Triple allylation of 1,3,5-triazine by acyl chlorides and allyltributyltin, and (**b**) possible reaction mechanism, according to [179], (where X = CO, R = Et).

A one-stage allylation/acylation of 1,3,5-triazine (**69**), allyltributyltin (**146**), and alkanoyl chlorides (**145**) was implemented [179]. The target product **147** is a mixture of syn,syn- and syn,anti-isomers, the structure of which has been confirmed by NMR and X-ray crystallography (Figure 46a).

A tentative reaction mechanism was rationalized [179] (Figure 46b). It is hypothesized that the *p*-electrons of the allyl tin reagent (**146**) act as a nucleophile on the electrophilic carbon atom formed by *N*-acylation of (**69**). The authors also pointed out that if the triple allylation reaction proceeded in successive stages, as postulated for the formation of monoallyl (**69a**), then the second and third allylations could occur with a 50% probability on either side of the ring. The result would therefore be a statistical mixture of isomers with a theoretical syn,syn-(**147**) (where X = CO, R = Et) yield of 25%, which is very close to the observed yield of ~35%. However, it was noted separately that the detailed mechanism of this reaction has not yet been fully studied.

### 2.4. Alkenylation and Alkynylation Reactions

#### 2.4.1. Metal-Free Alkenylation and Alkynylation Reactions 

##### 1,2,4-Triazines

**Figure 47:**
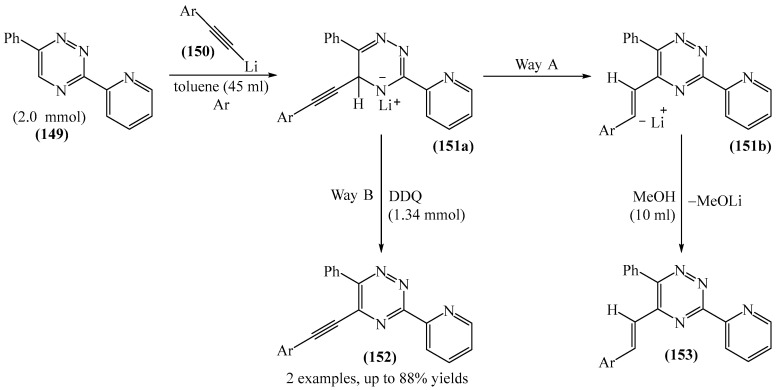
Reaction of 5-*H*-1,2,4-triazines with lithium-acetylenes, according to [180]. DDQ = 2,3-dichloro-5,6-dicyanobenzoquinone.

Chupakhin et al. [180] synthesized 5-arylethynyl-1,2,4-triazines (**152**) via direct C—H functionalization of 5-*H*-1,2,4-triazines (**149**) with lithium arylacetylenes (**150**) (Figure 47). This method may serve as a possible palladium-free alternative to Sonogashira cross-coupling.

Mechanistically, the reaction [180] starts with the formation of the corresponding σH adduct (**151a**), which then undergoes a 1,2-hydride shift to produce the styrene substituent. In the final stage, treatment of the reaction mixture with methanol delivers product **153**. To block pathway (**151a**), the reaction mixture must be treated with an oxidizing agent such as the 2,3-dichloro-5,6-dicyanobenzoquinone (DDQ). In which case, 5-styryl derivative (**152**) is not detected.

### 2.5. Annulation Reactions

#### 2.5.1. Metal-Catalyzed Annulation

##### Pyrazines

**Figure 48:**
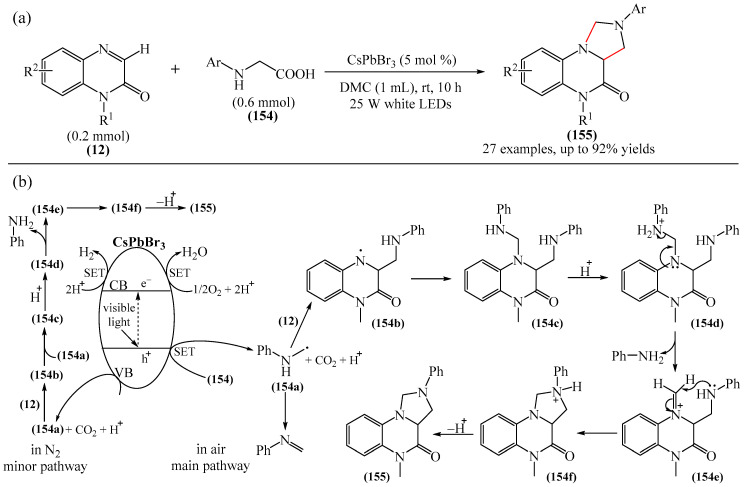
(**a**) Arylaminomethyl radical-initiated cascade annulation reaction of quinoxalin-2(1*H*)-ones, and (**b**) proposed reaction pathway, according to [114]. R^1^ = H, Me, Et, *n*-Pr, *n*-Bu, and others; R^2^ = H, Me, OMe, F, Cl, NO_2_, and others; Ar = C_6_H_5_, 4-ClC_6_H_4_, 4-FC_6_H_4_, 3-ClC_6_H_4_, 4-CF_3_C_6_H_4_, and others.

Chen and Yu et al. [114] developed a new methodology for the synthesis of a variety of polycondensed heterocycles, tetrahydroimidazo [1,5-*a*]quinoxaline-4(5*H*)-ones (**155**) via a one-pot reaction of diverse quinoxaline-2(1*H*)-ones (**12**) with *N*-arylglycines (**154**). The reaction proceeded in a green solvent (DMC) in the presence of CsPbBr_3_ under white light irradiation (Figure 48a).

Mechanistically (Figure 48b), the absorption of CsPbBr_3_ generates holes in the valence band (VB) and electrons in the conduction band (CB). Initially, a phenylaminomethyl radical (**154a**) is formed via single-electron transfer from *N*-phenylglycine (**154**) to a hole, releasing CO_2_ and a proton in the process. Radical (**154a**) is then added to *N*-methylquinoxaline-2(1*H*)one (**12**) at the C−3 position, delivering radical (**154b**), which rapidly adds another radical (**154a**), giving intermediate compound (**154c**). Radical (**154c**) is then protonated to afford intermediate compound (**154d**). Subsequently, an iminium cation (**154e**) is formed after the elimination of an aniline molecule (a good leaving group), from intermediate (**154d**). This is followed by an intramolecular nucleophilic attack that combines the phenylamino group with the iminium cation to form intermediate (**154f**). Finally, deprotonation of intermediate (**154f**) produces the final product (**155**). The protons released as a result of the reaction accept electrons from the conduction band and immediately react with O_2_ in the air to form a water molecule. Under anaerobic conditions, the released protons can alternatively acquire electrons directly from the conduction band to ultimately generate gaseous H_2_. Notably, the phenylaminomethyl radical (**154a**) is a key initiator of annulation in the reaction process; trace amounts of radical (**154a**) can be converted to *N*-phenylmethylamine (**154g**) through the removal of a hydrogen atom by another radical. The ion peak in HRMS at *m*/*z* 106.0648 corresponds well to (**154g**). Additionally, the authors presented the EPR structure of (**154g**), which, together with the HRMS data, confirms that the phenylaminomethyl radical can be obtained by photoredox-catalyzed decarboxylative oxidation of *N*-phenylglycine (**155**).

**Figure 49:**
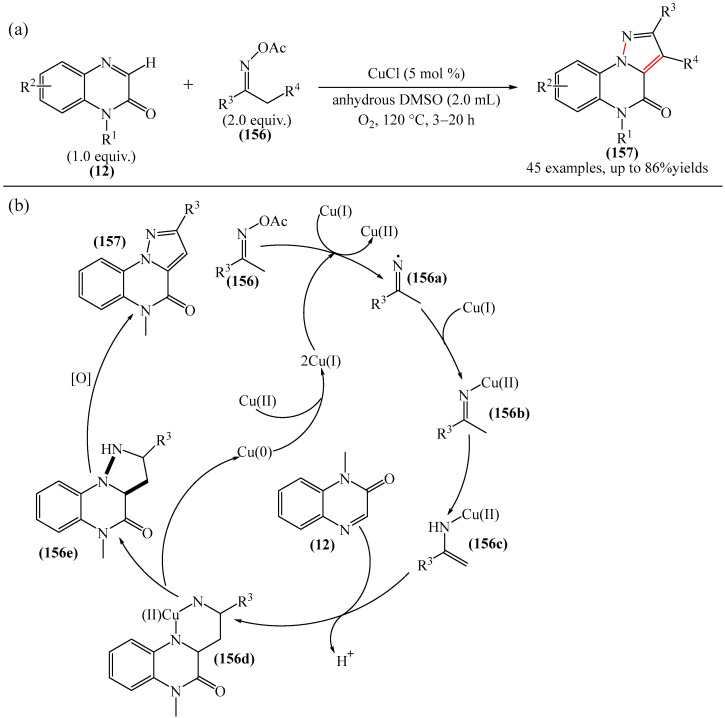
(**a**) Copper-catalyzed oxidative [3 + 2]-annulation of quinoxalin2(1*H*)-one with oxime esters toward functionalized pyrazolo [1,5-*a*]quinoxalin-4(5*H*)-ones, and (**b**) proposed reaction pathway, according to [181]. R = H, Me, Et, *n*-Pr, *n*-Bu, Allyl, and others; R^1^ = H, F, Cl, Br, OMe, CO_2_Me, and others; R^2^ = H, Me, C_6_H_5_, and others; R^3^ = H, Me, C_6_H_5_, and others.

The copper (CuCl)-catalyzed oxidative [3 + 2] annulation of quinoxaline-2(1*H*)-one (**12**) and oxime-O-acetates (**156**) was reported [181] (Figure 49a). Mechanistic studies have shown that the reaction proceeds via a radical pathway, forming a regioselective product (Figure 49b). Specifically, cleavage of the N-O bond of oxime acetate (**156**) in the presence of Cu(I) produces complex (**156b**) through the generation of an imine radical followed by complexation with Cu(I) and tautomerization. Complex (**156c**) then reacts with quinoxalinone (**12**) at the C3 position to form complex (**156d**), which generates dihydropyrazoloquinoxalinone (**156e**) through reductive elimination. Oxidation of structure (**156e**) with air delivers the more stable pyrazoloquinoxalinone (**157**).

**Figure 50:**
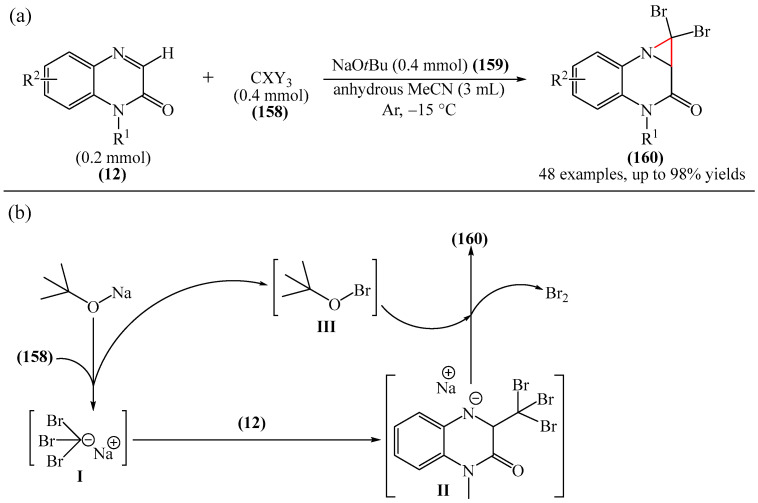
(**a**) Dihalo-aziridino quinoxalinones via C3-alkylation, and (**b**) proposed reaction pathway, according to [182]. R^1^ =Me, Et, Octyl, Allyl, and others; R^2^ = Me, F, Cl, Br, and others; CXY_3_ = CCl_4_, CBr_4_, CI_4_, CHBr_3_, CHCl_3_, CHI_3_, CBrCl_3_.

Bhat et al. [182] developed a base-mediated protocol for C3-alkylation of quinoxalinone (**12**) followed by tandem cyclization to produce new types of strained and condensed dihalo-aziridine-quinoxalinone heterocycles (**160**) through the formation of C−C and C−N bonds (Figure 50a). The reaction was carried out in the presence of a base, optimally NaO*t*Bu (2 equiv) (**159**), as well as in anhydrous MeCN and an argon atmosphere. The reaction mechanism was rationalized as follows [182]. Carbon tetrabromide (**158**) reacts with sodium *tert*-butoxide (**159**) to form the unstable intermediate carbanions [I] and [III]. The reactive carbanion [I] then reacts with *N*-methylquinoxaline-2(1*H*)-one (**12**) to produce the unstable salts [II]. The nitrogen anion of intermediate [II] rapidly intramolecularly attacks the electrophilic β carbon, eliminating bromide to generate the desired product **160**. The bromide anion then reacts with intermediate compound [III] to deliver bromine and sodium *tert*-butoxide. Once the reaction has finished, the reaction mixture becomes slightly acidic (decrease in pH), which confirms the release of bromine. This probably explains why an excess amount of sodium *tert*-butoxide (2 equiv.) is required to maintain the alkalinity of the reaction medium and to accelerate the reaction for this conversion.

## 3. C−O Bond Formation

### 3.1. Metal-Free Reactions of C−O Bond Formation

#### Pyrazines

**Figure 51:**
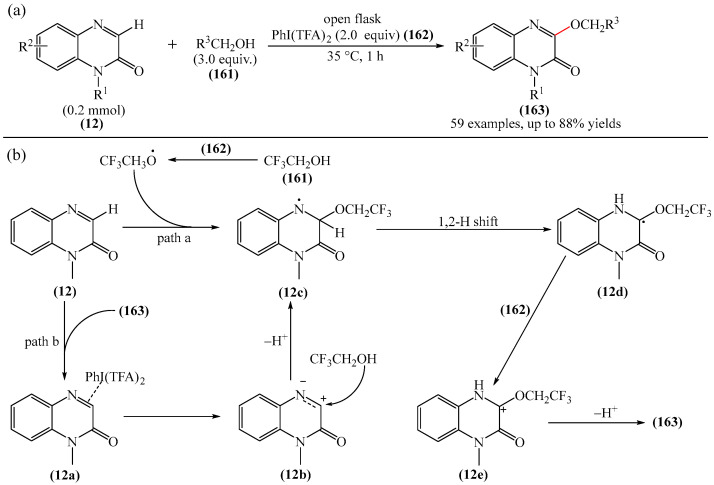
(**a**) Transition metal and solvent-free oxidative C−H fluoroalkoxylation of quinoxalinones with fluoroalkyl alcohols, and (**b**) plausible mechanism, according to [52]. R^1^ = H, Me, Et, *n*-Bu, and others; R^2^ = H, Me, MeO, Cl, Br; R^3^ = CF_3_ = CH_2_F, CHF_2_, C_2_F_5_, and others.

Zhang et al. [52] reported a solvent- and transition metal-free approach to the C—H fluoralkoxylation of quinoxalinones (**12**) with fluoroalkyl alcohols (**161**). This reaction provides a facile and straightforward method for the synthesis of previously unknown fluoroalkoxy derivatives of quinoxalines and also enables the amination of quinoxalinones (**163**) (Figure 51a). Furthermore, this reaction occurred under very mild conditions and did not require an inert atmosphere. A SET mechanism was proposed [52] (Figure 51b). Initially, a trifluoroethoxyl radical is formed from trifluoroethanol through the oxidation of PhI(TFA)_2_ (**162**). This radical then attacks substrate (**12**) to produce an intermediate nitrogen radical (**12c**) (path a). An alternative pathway for the formation of the nitrogen radical intermediate (**12c**) involves the coordination of substrate (**12**) with PhI(TFA)_2_ (**162**) to deliver intermediate compound (**12a**). Then, as a result of the SET process, a reactive cation radical (**12b**) is generated and captured.

**Figure 52:**
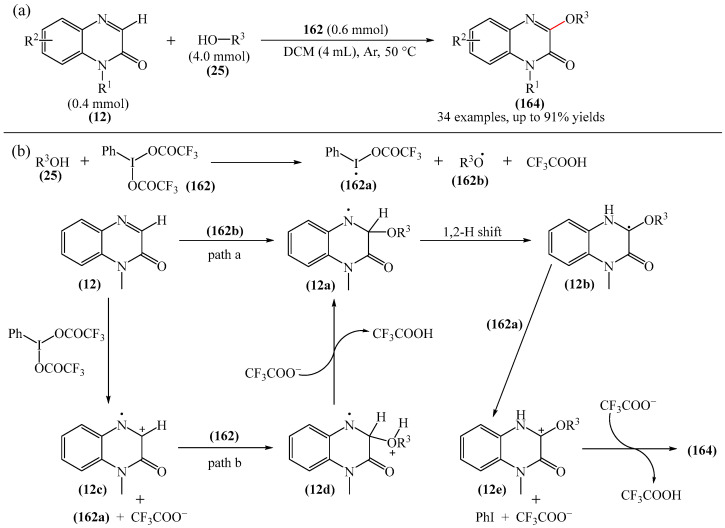
(**a**) Direct C3 alkoxylation of quinoxalin-2(1*H*)-ones with alcohols via cross-dehydrogenative coupling, and (**b**) proposed reaction mechanism, according to [183]. R^1^ = Me, Et, Bn, and others; R^2^ = H, Me, F, Cl, COOMe, and others; R^3^ = Et, *n*-Pr, *i*-Pr, O-*n*-C_4_H_9_, and others.

Another method for the direct C3-alkoxylation of quinoxaline-2(1*H*)-ones (**12**) with alcohols (**25**) via dehydrogenative cross-coupling under non-catalytic conditions was described in [183] (Figure 52a). This method provides powerful and convenient access to 3-alkoxyquinoxaline-2(1*H*)-ones (**164**) in good to excellent yields, using PhI(TFA)_2_ (**162**) as an oxidant. It also allows for the straightforward preparation of potential drug molecules containing 3-alkoxyquinoxaline-2(1*H*)-one skeletons. The proposed mechanism [183] involves the following reaction sequence shown in Figure 52b, which is essentially similar to the scheme shown in Figure 51b. Initially, (**162**) reacts with an alcohol to form an I-radical (**162a**), an alkoxy radical (**162b**), and trifluoroacetic acid. Subsequently, the alkoxy radical (**162b**) is captured by substrate (**12**) to generate the intermediate aminium radical (**12a**) (path a). An alternative pathway to intermediate (**12a**) (path b) is also possible: in the presence of (**162**), substrate (**12**) is oxidized to deliver cation radical (**12c**). This is then captured by nucleophilic alcohols to afford intermediate (**12e**). The key intermediate (**12a**) is obtained by deprotonation of intermediate (**12d**) with trifluoroacetate. Afterwards, the intermediate (**12a**) undergoes 1,2-hydrogen shift to produce the intermediate carbon radical (**12b**), which reacts with I-radical (**162a**) via a one-electron transfer process to form intermediate carbon cation (**12e**). This is accompanied by the release of iodobenzene and trifluoroacetate. Finally, the target product **164** is obtained by deprotonation of intermediate (**12e**) in the presence of trifluoroacetate.

**Figure 53:**
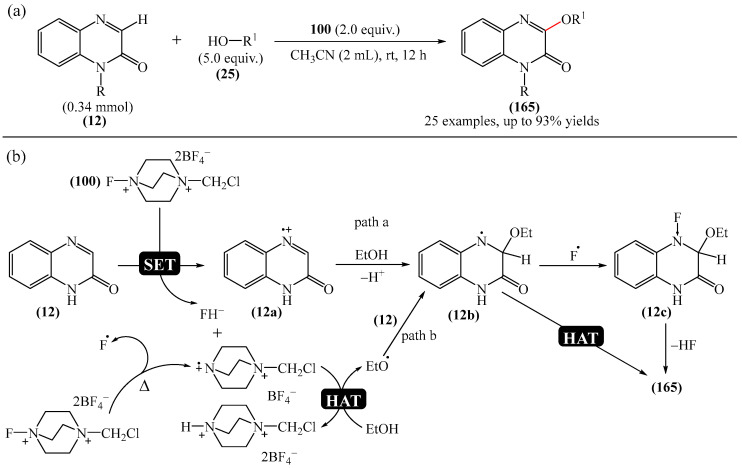
(**a**) Selectfluor-mediated regioselective C−3 alkoxylation of quinoxalin-2(1*H*)-ones, and (**b**) proposed reaction mechanism, according to [184]. R = H, Me, Et, Bu, and others; R^1^ = Et, Pr, *i*-Pr, CH_2_-C_6_H_5,_ and others.

Kumar et al. [184] presented an elegant method for the alkoxylation of quinoxalin-2(1*H*)-ones (**12**) (Figure 53a).

They studied the scope of the reaction with various alcohol substrates (**25**), including primary, secondary, allylic, propargyl and benzyl alcohols, and demonstrated that depending on their structure, these alcohols can react smoothly with **12** to produce the desired products **165** in yields of up to 93%. According to the proposed mechanism (Figure 53b), radical (**12b**), a derivative of quinoxaline-2(1*H*)-one (**12**), can be generated by two different routes: either by generation of intermediate product (**12a**) via single-electron transfer (SET) from Selectfluor (**100**) followed by nucleophilic attack of ethanol on (**12a**) (path a), or by attack of an ethoxy radical formed via hydrogen atom transfer (HAT) to quinoxaline-2(1*H*)-one (**12**) (path b). Finally, product **165** is obtained either by HAT from intermediate compound (**12b**) or by removal of HF from intermediate compound (**12c**), which is formed when a fluorine radical binds to intermediate compound (**12b**).

**Figure 54:**
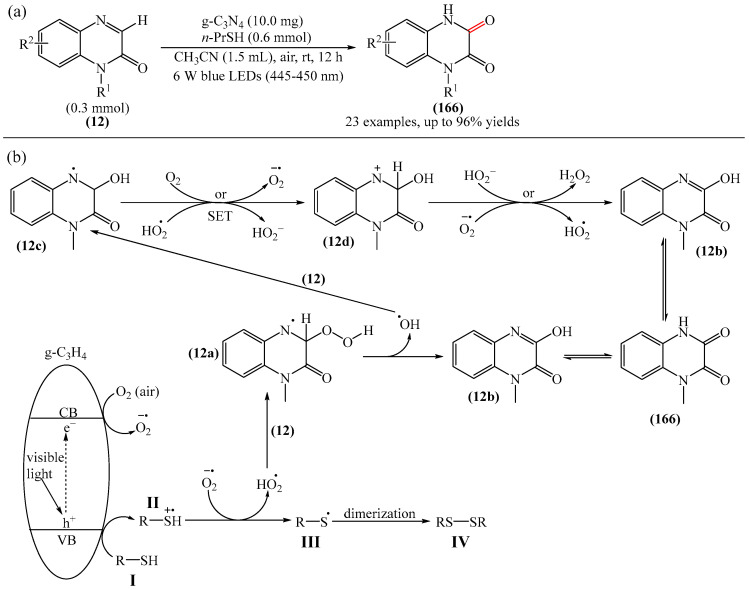
(**a**) Visible light-induced recyclable g-C_3_N_4_ catalyzed C–H hydroxylation of quinoxalin-2(1*H*)-ones, and (**b**) proposed reaction mechanism, according to [185]. R^1^ = H, Me, Et, Bu, Bn, and others; R^2^ = H, F, Cl, Br, CF_3_, and others.

A method for the visible light-promoted hydroxylation of quinoxaline-2(1*H*)ones (**12**), employing recyclable graphitic carbon nitride (g-C_3_N_4_) as a heterogeneous photocatalyst was elaborated [185] (Figure 54a). The authors also investigated other photocatalysts, such as TiO_2_, CdS and CsPbBr_3_, but the yields were significantly lower. The influence of the light source was examined; lower yields were observed with blue, white and sunlight, while no hydroxylation product was obtained using a green light source. The effect of the nature of the homogeneous photocatalyst was evaluated. For example, the potential of compounds such as Eosin Y (**128**), Rose Bengal (**17**), Rhodamine 6G, and Ru(bpy)_3_Cl_2_ as catalysts were investigated. Compared to g-C_3_N_4_, these homogeneous photocatalysts produced lower yields of the target product **166**. The main by-product was found to be 1-methyl-3-(propylsulfanyl)quinoxaline-2(1*H*)-one, likely formed through C—H sulfenylation of *N*-methylquinoxaline-2(1*H*)-one. Furthermore, the hydroxylation product was not detected in the absence of a photocatalyst or in the dark.

A tentative reaction mechanism was rationalized as follows [185] (Figure 54b). In the beginning, g-C_3_N_4_ is excited by visible light, generating an electron in the conduction band (CB) and a hole in the valence band (VB). The hole generated in VB (E_VB_ = +1.2 B vs. SCE) accepts an electron from thiol I (E_1/2_^ox^ = +0.50 B vs. SCE) to generate cation radical II through a single-electron transfer process. At the same time, an electron from CB (E_CB_ = −1.5 B vs. SCE) is transferred to O_2_ (air) to form O_2_^•−^ (E^red^ [O_2_/O_2_^•−^] = −0.8 B vs. SCE), which was confirmed by EPR using 5,5-dimethyl-1-pyrroline (DMPO) as a spin trap. The O_2_^•−^ then abstracts hydrogen from the thienyl radical cation II to produce HO_2_^•^ and thienyl radical III. The resulting HO_2_^•^ can selectively attack the C−3 position of **12** to generate intermediate (**12a**). Under the action of visible light, the intermediate compound (**12a**) can undergo homolytic cleavage to deliver (**12b**) and a hydroxyl radical HO^•^ The latter is also added to the C=N bond of compound **12**, producing intermediate nitrogen radical (**12c**), which is further oxidized by HO_2_^•^ or O_2_ to form intermediate nitrogen cation (**12d**). Deprotonation of compound (**12d**) gives intermediate (**12b**). Finally, intermediate (**12b**) undergoes rapid tautomerization to afford the more stable product **166**. In this process, thiyl radical III can rapidly dimerize to disulfides IV.

**Figure 55:**
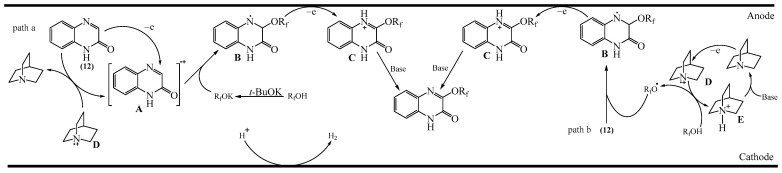
Putative reaction mechanism, according to [186].

Jin, Han, and Zhang described [186] the first electricity-driven and metal-free C(3)H-fluoroalkoxylation of quinoxaline-2(1*H*)-ones (**12**) with both α-monosubstituted and α,α-disubstituted fluoroalkyl alcohols. In total, 27 compounds were synthesized in 29–94% yields. The mechanism proposed by the authors [186] is shown in Figure 55.

In pathway A, substrate **12** was initially oxidized at the anode or by a quinacridine radical cation formed via the oxidation of quinacridine (**55**) at the anode. This resulted in the formation of intermediate **A**, which underwent nucleophilic attack by fluoroalkoxy potassium salt in the presence of hexafluoroisopropanol and *t*-BuOK (**117**) to generate intermediate **B**. After oxidation of intermediate **B** at the anode to protonated intermediate **C**, further neutralization with a base delivered the desired product **164**. In pathway B, quinacridine (**55**) could also be oxidized at the anode to form the intermediate quinacridine radical cation **D**. Subsequently, the cation **D** oxidized hexafluoroisopropanol to produce fluoroalkoxy radicals and the protonated intermediate quinacridine **E** via SET. The resulting fluoroalkoxy radical then attacked substrate **12** to afford intermediate **B**, after which the protonated intermediate **C** was formed at the anode via oxidation, which was neutralized to form the desired product **3** in the presence of a base. It was shown [186] that two pathways exist simultaneously for this reaction, with pathway A being the main reaction pathway.

An effective approach to the preparation of quinoxaline-2,3-diones was developed [187]. The reaction occurred due to acid-self-photocatalyzed regioselective oxidation of quinoxaline-2(1*H*)-ones at the C−3 position in the presence of Brønsted acids, described using air (O_2_) as a green oxidant. The most efficient protocol was realized at room temperature for 12h, employing CF_3_COOH, MeCN, and 3 W blue LEDs.

Wang and Shen et al. [188] documented the first photoinduced oxidative CH-esterification of quinoxalinones with arylaldehydes, which proceeds at room temperature in a nitrogen atmosphere using hydrogen peroxide (**50**) as an oxidant, and LEDs (390 nm, 20 W).

## 4. C−B Bond Formation

### 4.1. Metal-Free Reactions of C−B Bond Formation

#### Pyrimidines

**Figure 56:**
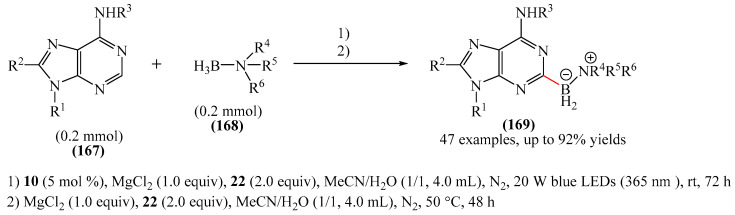
C^2^−H borylation of unprotected adenosine and adenine derivatives via Minisci reaction, according to [50]. R^1^ = adenosine derivatives, Me; R^2^ = H, Me, C_6_H_5_, 4-MeC_6_H_4_, 4-MeOC_6_H_4_, 4-FC_6_H_4_; R^3^ = H, C_6_H_5_-CH_2_-, and others; R^3^ = R^4^ = R^5^ = Me, Et, Cy, and others.

The Minisci-type borylation of molecules containing a pyrimidine fragment (**167**) via photocatalysis or thermal activation was reported [50] (Figure 56). This method tolerates a wide variety of nitrogen-containing heterocycles (**169**), including several important biomolecules and well-known antiviral and antitumor drugs, such as AMP, cAMP, Vidarabine, Cordycepin, Tenofovir, Adefovir, GS-441524, etc. This approach provides direct access to valuable and structurally novel C^2^-borylated purine derivatives (**167**) that would otherwise be difficult to obtain. Diverse secondary and tertiary borane complexes, in addition to trimethylamine (**168**), can be used as boron radical precursors to functionalize the C^2^ position of the purine skeleton with unprecedented functional groups.

## 5. C−N Bond Formation (Amination, Amidation, Oxidative Amidation)

### 5.1. Metal-Free Reactions

#### Pyrazines

**Figure 57:**
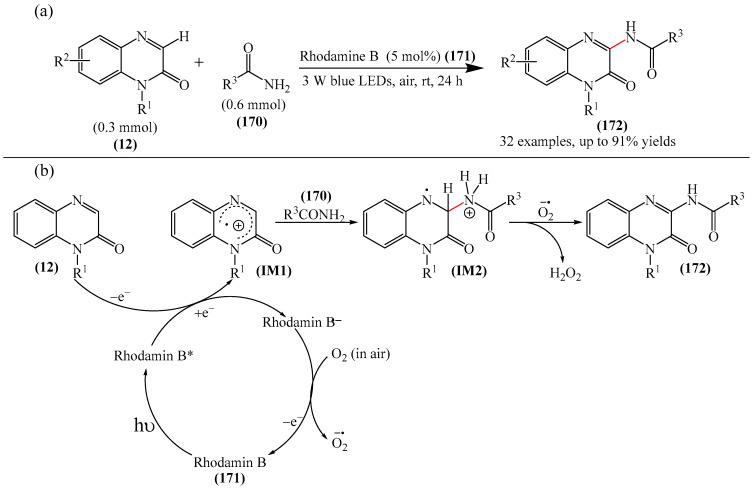
(**a**) Visible light-initiated cross-dehydrogenative coupling of quinoxalin-2(1*H*)-ones and simple amides with air as an oxidant, and (**b**) postulated reaction pathway, according to [189]. R^1^ = Me, Et, Bn, and others; R^2^ = H, F, Cl, Br, CF_3_, and others; R^3^ = C_6_H_5_, 4-MeC_6_H_4_, 4-*t*-BuC_6_H_4_, 4-ClC_6_H_4_, and others.

He et al. [189] reported an efficient method for the synthesis of various *N*-acylated 3-aminoquinoxalin-2(1*H*)-ones (**172**) through visible light-catalyzed amidation of quinoxalin-2 (1*H*)-ones (**12**) and amides (**170**) using Rhodamine B (**171**) as a photocatalyst and air as an oxidant in the absence of metals and strong oxidants (Figure 57a). This method can be considered as one of the possible alternatives to that described in [104].

A plausible reaction mechanism was proposed [189] (Figure 57b). Initially, under the influence of visible light, the photocatalyst Rhodamine B (**171**) was transformed into excited Rhodamine B*, which underwent a single-electron transfer process with quinoxalinone (**12**) to give the corresponding intermediate cation radical IM1 and Rhodamine B anion radical. In the presence of air (molecular oxygen), the Rhodamine B anion radical was oxidized to Rhodamine B in the ground state to generate a superoxide anion radical (O_2_^•−^). Subsequently, an intermolecular nucleophilic attack of amide (**170**) on intermediate IM1 resulted in the formation of intermediate IM2, which was then oxidized by O_2_^•−^ to produce the expected product (**172**) and release hydrogen peroxide (**50**). Support for this mechanism comes from the detection of in situ generated H_2_O_2_ using hydrogen peroxide test strips.

**Figure 58:**
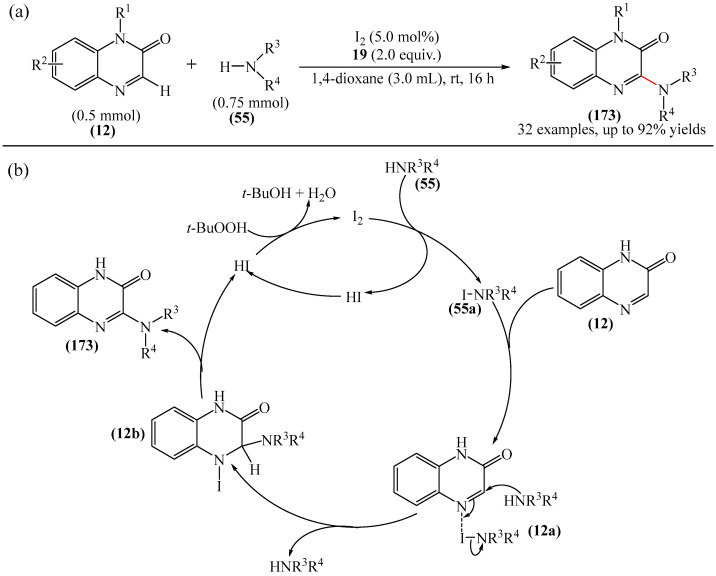
(**a**) Synthesis of 3-aminoquinoxalinones by direct C−H amination, and (**b**) proposed mechanism for iodine-catalyzed amination of quinoxalinones, according to [190]. R^1^ = H, Alkyl, CH_2_-C_6_H_5_, and others; R^2^ = H, F, Cl, and others; R^3^ = R^4^ = secondary aliphatic amines ranging from cyclic to acyclic derivatives.

Metal-free synthesis of 3-aminoquinoxalines (**173**) based on non-functionalized quinoxalines (**12**) and amines (**55**) was described [190] (Figure 58a).

This versatile reaction demonstrates good tolerance to a number of primary and secondary amines, producing 3-aminoquinoxalines (**173**) in moderate to high yields. Formation of the new C−N bond is effectively achieved under metal-free conditions, without any ligands or additives, delivering harmless by-products such as *tert*-butyl alcohol and water. The practical usefulness of this method is demonstrated by the synthesis of a pharmaceutically active aldolase inhibitor and histamine-4 receptor antagonist. Mechanistic studies show (Figure 58b) that the reaction proceeds via an ionic mechanism involving amine iodination as the proposed mode of activation. Given the pharmaceutical importance of these molecules, this modern methodology provides atom economic and efficient access to them.

C−3 amination of quinoxaline-2(1*H*)-ones (**12**) was also implemented [191]. However, unlike the methods disclosed in [190] and [192], this protocol was based on the visible light-induced direct amination of the biologically important C−3 fragment of quinoxaline-2(1*H*)-one in the presence of an EDA complex. The formation of a photoactivated donor–acceptor complex between quinoxaline-2(1*H*)-one (**12**) and amine (**55**) is key to the success of this method, as this complex subsequently undergoes an electron transfer reaction to achieve the desired conversion. Using this process, a diverse set of 3-aminoquinoxaline-2(1*H*)-ones was obtained, in yields of up to 87%.

**Figure 59:**
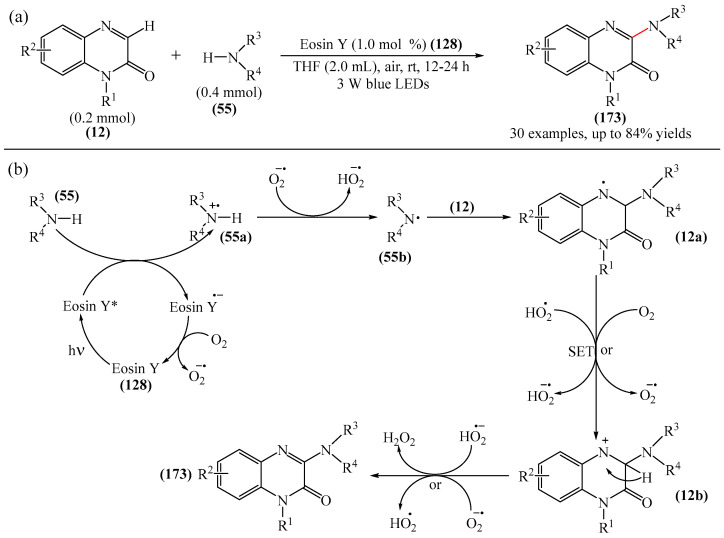
(**a**) Results for visible light-promoted C(sp^2^)-H/N-H cross-dehydrogenative coupling amination of quinoxalinones with aliphatic amines, and (**b**) possible reaction pathway, according to [193]. R^1^ = H, Me, Et, *n*-Pr, *n*-Bu, and others; R^2^ = H, F, Cl, Br; R^3^ = R^4^ = H, Alkyl, CH_2_-C_6_H_5_, and others.

Wei et al. [193] developed a novel and efficient visible light-induced method for C(sp^2^)-H/N-H cross-dehydrogenative coupling (CDC) amination with both primary and secondary aliphatic amines (**55**) at room temperature in air (Figure 59a). This photocatalytic reaction enables the direct formation of 3-aminoquinoxaline2(1*H*)-ones (**173**) via CDC amination without the need for an external oxidant.

Preliminary mechanistic studies show that this reaction proceeds via a radical process (Figure 59b). Specifically, Eosin Y (**128**) is initially irradiated with LED light to form Eosin Y* in an excited state. The latter receives an electron from amine (**55**), generating the anion radical Eosin Y^•−^ and the nitrogen cation radical (**55a**). Subsequently, the oxidation of Eosin Y^•−^ by molecular oxygen (air) produces the ground state Eosin Y and O_2_^•−^. Deprotonation of the nitrogen cation radical (**55a**) by O_2_^•−^ then delivers a hydroperoxide radical (HO_2_^•^) and an intermediate nitrogen radical (**55b**). The addition of nitrogen radical (**55b**) to quinoxaline-2(1*H*)-one (**12**) affords intermediate (**12a**), which is oxidized by O_2_ or HO_2_^•^ to give intermediate nitrogen cation 7 via a SET process. Finally, deprotonation of intermediate (**12b**) regenerates the aromatic ring and yields the desired product **173**. It is noteworthy that this mechanism was proposed [193] on the basis of previously published data, and EPR studies.

**Figure 60:**
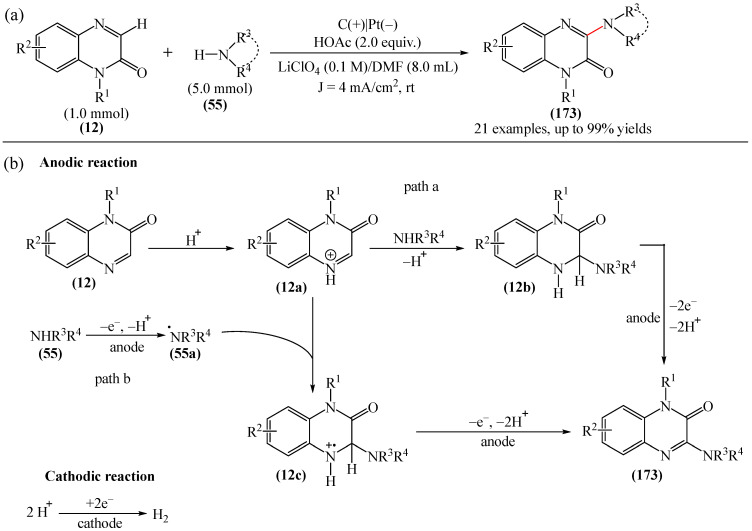
(**a**) Electrochemical dehydrogenative cross-coupling of quinoxaline-2(1*H*)-ones with amines for the synthesis of 3-aminoquinoxalinones, and (**b**) two possible pathways for the electrochemical oxidative amination of quinoxaline-2(1*H*)-ones, according to [194]. R^1^ = H, Me, CH_2_-CO_2_Et, and others; R^2^ = H, F, Cl, Me, OMe, and others; HNR^3^R^4^ = aliphatic amines and azoles.

An alternative to the methods described in References [192,193] was presented in [194]. This is an effective protocol for the synthesis of 3-aminoquinoxalinones (**173**) by electrochemical dehydrogenative C−3-amination of quinoxaline-2(1*H*)-ones (**12**) (Figure 60a). Using aliphatic amines (**55**) and azoles as nitrogen sources, a series of 3-aminoquinoxalines (**173**) was obtained in a yield of up to 99%. While this direct electrolytic method avoids the use of transition metals and external oxidants, it does require an appropriate electrolytic cell.

Two possible mechanisms for the electrochemical oxidative amination of quinoxaline-2(1*H*)-ones (**12**) were postulated [194] (Figure 60b). According to the ionic mechanism, the process begins with the nucleophilic addition of the amine substrate **55** to the protonated quinoxaline-2(1*H*)-one, (**12a**), forming the intermediate (**12b**). After anodic oxidation and deprotonation, this intermediate generates product **173** (path a). In the radical pathway, the anodic oxidation of amines (**55**) produces a nitrogen-centered radical (**55a**), which is then intercepted by (**12a**) to give intermediate (**12c**). Further anodic oxidation and deprotonation delivers the desired products **173** (path b). Simultaneously, the proton is reduced on the cathode surface to afford molecular hydrogen.

The C(sp^2^)-H amination processes of 3-aminoquinoxalin-2(1*H*)-ones in the presence of compounds such as ceric ammonium nitrate [195] are known.

**Figure 61:**
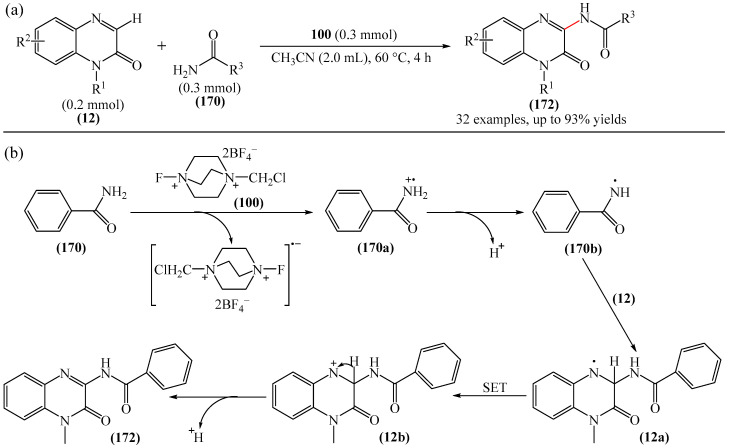
(**a**) Synthesis of 3-amidated quinoxalin-2(1*H*)-one derivatives, and (**b**) proposed reaction mechanism, according to [196]. R^1^ = H, Me, Et, Pr, Bu, and others; R^2^ = H, Cl, Br; R^3^ = C_6_H_5_, 4-X-C_6_H_4_ (X = F, Cl, Br, CF_3_, and others), and others.

Yuan et al. [196] carried out C3-amidation of quinoxaline-2(1*H*)-ones (**12**) using Selectfluor (**100**) as a mild oxidant, without the use of transition metal catalysts (Figure 61a). It was suggested that the transformation proceeds via a radical pathway (Figure 61b).

**Figure 62:**
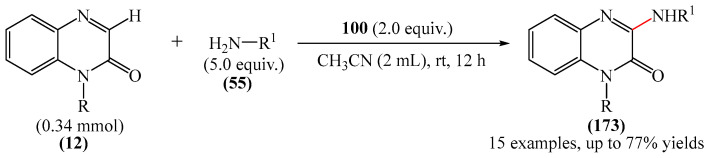
Selectfluor-mediated regioselective C−3 amination of quinoxalin-2(1*H*)-ones, according to [184]. R = H, Me, Rt, Bu, CH_2_-C_6_H_5_, CO-C_6_H_5_, and others; R^1^ = Pr, cyclohexyl, CH_2_-C_6_H_5_.

In addition to alkoxylation reactions, the above-mentioned work [184] also reports on amination that proceeds under mild conditions in the absence of transition metal complexes (Figure 62). A total of 15 compounds were obtained in yields of 60–77%.

**Figure 63:**
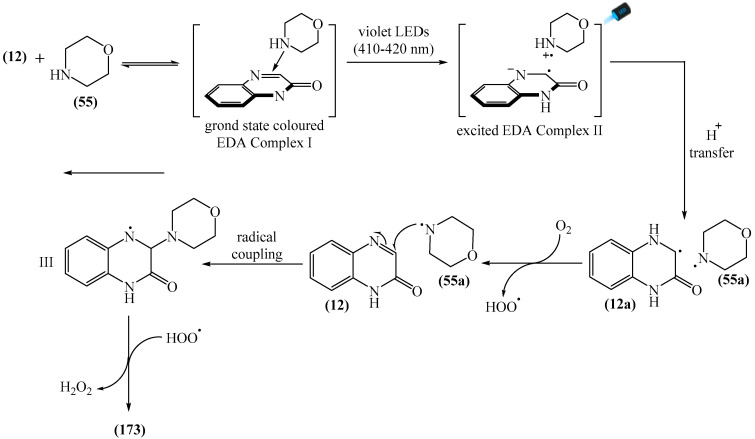
Plausible reaction mechanism, according to [191].

Paper [191] was first to describe the EDA complex-mediated, metal and photocatalyst-free, visible light-initiated direct amination of the biologically important C−3 fragment of quinoxaline-2(1*H*)-one. The formation of a photoactivated donor–acceptor complex between quinoxaline-2(1H)-one and an amine is the key step in this process, and subsequent electron transfer is required to achieve the desired conversion. A total of 30 compounds were synthesized in 30–87% yields. The plausible mechanism is shown in Figure 63.

The reaction begins with the formation of EDA complex I between quinoxaline-2(1*H*)-ones (**12**) and morpholine (**55**). When exposed to visible light, single-electron transfer occurred (excited EDA complex II), and the subsequent transfer of a proton generates quinoxaline-2(1*H*)-one radical (**12a**) and a morpholine radical (**55a**). The radical intermediate **12a** reacts with oxygen, regenerates **12**, and forms a hydroperoxyl radical. Next, the morpholine radical **55a** reacts with **12** to form intermediate III, which produces the desired product **173** and hydrogen peroxide (H_2_O_2_) upon oxidation. The formation of H_2_O_2_ in the reaction mixture was detected by an iodometric experiment.

Ou and He [197] first reported a sonophotocatalytic coupling by purple LED (390 nm, 2 W) and 44 KHz ultrasound irradiation. Using visible light and ultrasonic waves as energy sources, various 3-aminoquinoxalin-2(1*H*)-ones with good tolerance to functional groups were obtained. The reaction required no additives or external photocatalysts. Totally, 37 products were synthesized in 62–86%. It was noted that sonophotocatalysis significantly improves the reaction rate and product yields compared to traditional photocatalysis, while also reducing energy consumption.

A method for the amination of quinoxaline-2(1*H*)-ones with aliphatic amines using DABCO (**18**) in the presence of mpg-C_3_N_4_ (mesoporous graphitic carbon nitride) mpg-C_3_N_4_ was explained [198]. This process was conducted under blue LEDs (450–455 nm) and oxygen at room temperature for 24–48 h. Meanwhile, an alternative method involving a graphitic carbon nitride (g-C_3_N_4_) catalyst was presented in [199].

In addition to the methods described above, the amination of quinoxaline-2(1*H*)-ones can be carried out in the presence of *tert*-butyl nitrite and 18 W blue LED [200].

### 5.2. Metal-Catalyzed Reactions

#### Pyrazines

**Figure 64:**
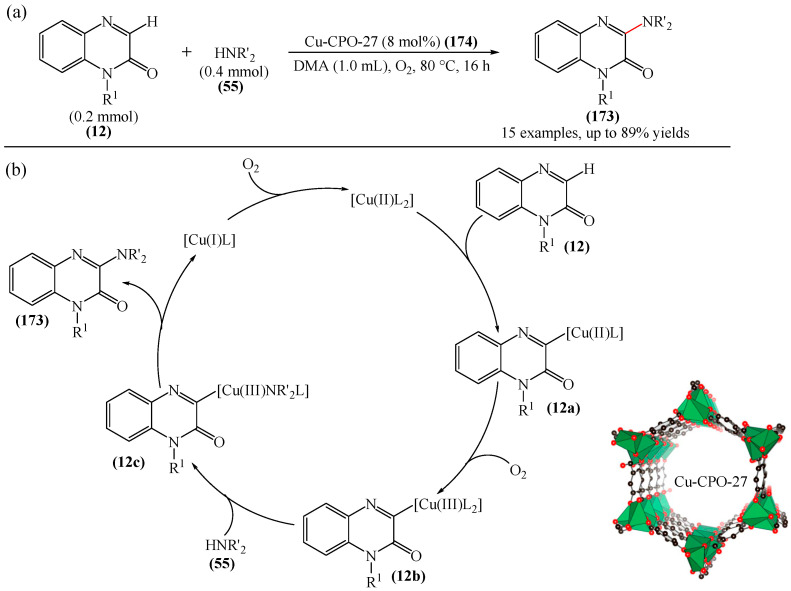
(**a**) The direct C—H amination of quinoxalin-2(1*H*)-one with amines utilizing Cu-CPO-27 catalyst, and (**b**) proposed mechanism for the direct C—H amination reaction, according to [192]. R = H, *n*-Bu; HNR’_2_ = secondary and primary amines (cyclic and aliphatic).

Another interesting example of direct oxidative C—H amination of quinoxalinones (**12**) under copper-organic framework catalysis was documented [192] (Figure 64a).

The catalytically active structure employed was the Cu-CPO-27 (**174**) framework: a honeycomb metal–organic framework (MOF) formed by the coordination of tetraprotonated 2,5-dihydroxyterephthalic acid fragments with Cu(II) ions. However, the products were obtained in the presence of molecular oxygen, which was used as an oxidant. This can be considered a significant drawback of this methodology.

The reaction mechanism was proposed [192] (Figure 64b). It was suggested that, initially, deprotonation of quinoxaline-2(1*H*)-one (**12**) leads to the formation of pyridine copper (II) particles (**12a**), probably due to the acidity of the C—H bond α to nitrogen. Subsequently, oxidation of copper (II) with oxygen produces copper (III) species (**12b**), which underwent nucleophilic substitution with morpholine to deliver amino copper (III) species (**12c**). Reductive elimination followed by single-electron oxidation regenerates the initial catalyst. However, it was noted that further research is needed to clarify the details of the mechanism.

**Figure 65:**
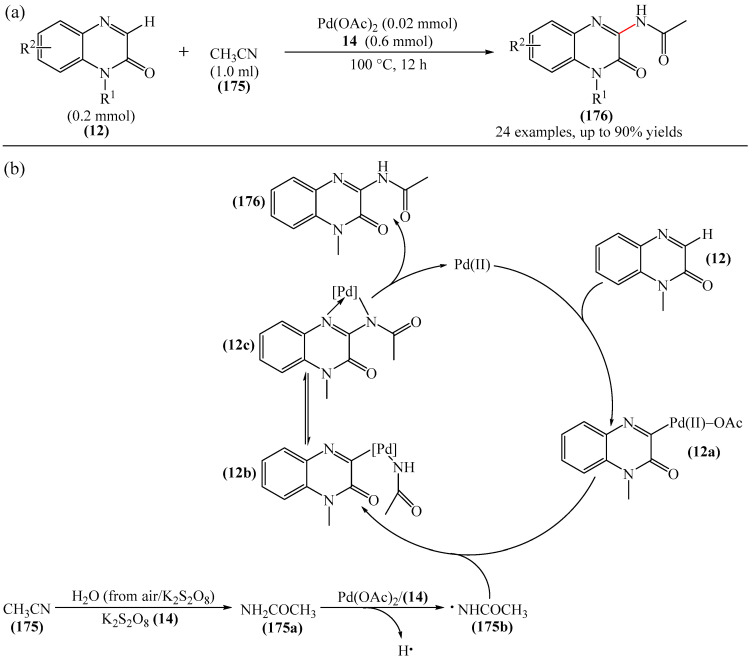
(**a**) Synthesis of 3-amidated quinoxalin-2(1*H*)-ones, and (**b**) proposed reaction mechanism, according to [72]. R^1^ = Me, Et, Pr, Bu, and others; R^2^ = H, F, Cl, Br, NO_2_.

A method for the oxidative amidation of quinoxalin-2(1*H*)-ones (**12**) with acetonitrile (**175**), delivering the target products **176** in good to excellent yields, was developed [72] (Figure 65a).

Optimal yields were achieved in the presence of K_2_S_2_O_8_ as an oxidant and Pd(OAc)_2_ as a catalyst. However, the use of palladium-based catalysts, although widespread, is still a significant disadvantage of this method due to their expense and limited availability. In addition to monitoring possible substrates, a plausible reaction mechanism was (Figure 65b). Acetonitrile (**175**) reacts with H_2_O in the presence of potassium peroxydisulfate (**14**) to form acetamide (**175a**). Then, in the presence of Pd(OAc)_2_ and potassium peroxydisulfate (**14**), acetamide leaves a hydrogen radical to generate intermediate (**175b**). Metallization of 1-methylquinoxalin-2(1*H*)-one (**12**) under the action of Pd(OAc)_2_ produces intermediate (**12a**), which undergoes oxidative addition with radical (**175b**) to give Pd(III) complex (**12b**). The latter is further oxidized to Pd(IV) (**12c**) by a photo-oxidative–reductive catalytic act and/or through the action of an oxidizing agent. It was mentioned [72] that the role of K_2_S_2_O_8_ in the oxidation of Pd(III)/Pd(IV) in these catalytic systems is unclear and requires further study. Intermediate compound (**12c**) may be formed as a result of transmetalation. The desired product **176** can be formed by easy reductive removal of intermediate compound (**12c**) and formation of Pd (II) catalyst for subsequent experiments.

**Figure 66:**
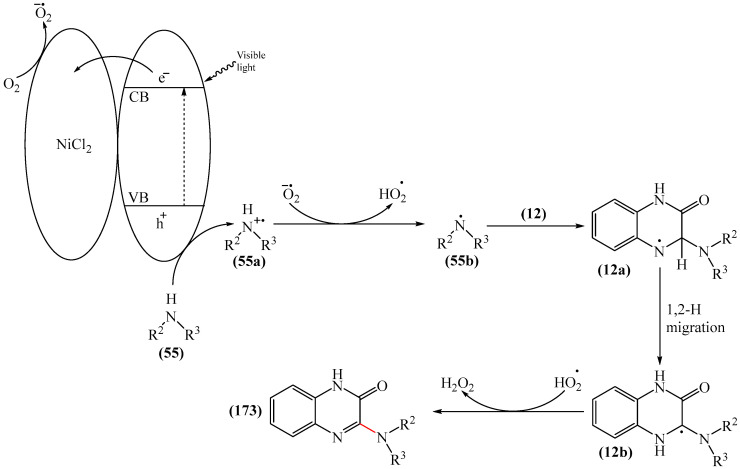
Plausible reaction mechanism, according to [201].

Ke et al. [201] reported on a new photoactive catalytic system based on NiCl_2_@g-C_3_N_4_ that effectively stimulates the amination of quinoline-2(1*H*)-ones with primary and secondary aliphatic amines under blue light irradiation. A total of 26 model products were obtained in yields of 74–90%. It was demonstrated that the nickel ion could act as an electron acceptor, thereby enhancing the carrier separation and transfer and significantly improving the efficiency of the photoreaction for g-C_3_N_4_ (Figure 66). Preliminary mechanistic studies indicate that, upon irradiation with visible light, photo-generated electrons and holes act as oxidants, producing amine radicals in the absence of external chemical oxidants. This provides a simple and effective approach to the direct 3-amination of quinoxaline-2(1*H*)-ones. Furthermore, it was emphasized that NiCl_2_@g-C_3_N_4_ catalyst can be used at least six times without significant loss of activity.

Initially, blue light irradiation caused charge separation in NiCl_2_@g-C_3_N_4_ and produced a photo-generated hole (h^+^) and a photo-generated electron (e^−^). The holes then received electrons from amines (**55**), forming a nitrogen cation radical (**55a**) through single-electron transfer. Simultaneously, electrons in the conduction band were transferred to O_2_ (air) to form O_2_^•−^. Next, deprotonation of the nitrogen cation radical by O_2_^•−^ delivered a hydroperoxide radical (HO_2_^•^) and an intermediate nitrogen radical (**55b**), which was transformed into an intermediate nitrogen radical **12a**. The latter underwent further oxidation involving HO_2_^•^ or O_2_, forming the intermediate radical **12b**. Finally, deprotonation of the intermediate nitrogen cation **12b** afforded product (**173**).

#### 1,3,5-Triazines

**Figure 67:**
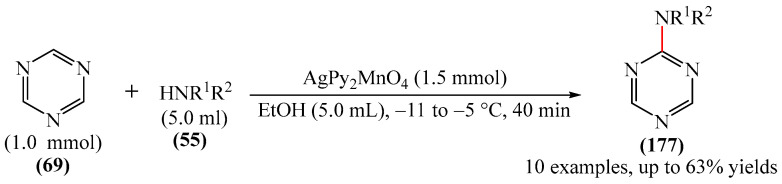
Oxidative amination of 1,3,5-triazine, according to [202]. R^1^ = H, piperidin-1-yl, morpholin-1-yl; R^2^ = H, Me, *i*-Pr, *n*-Bu, *n*-C_5_H_11_.

Maes et al. [202] described the first examples of nucleophilic substitution of hydrogen on 1,3,5-triazine (**69**). Specifically, they demonstrated the potential for oxidative (alkyl)amination of azines (**177**) on 1,3,5-triazine (**69**) using AgPy_2_MnO_4_ as an oxidant (Figure 67). The yield of the target product **177** depended largely on the nature of the alcohol used as a cosolvent; ethanol, for example, was more effective than methanol. The reaction yield was unaffected by using tetrahydrofuran instead of ethanol as a cosolvent.

## 6. C−S Bond Formation

### 6.1. Metal-Free Reactions

#### Pyrazines

**Figure 68:**
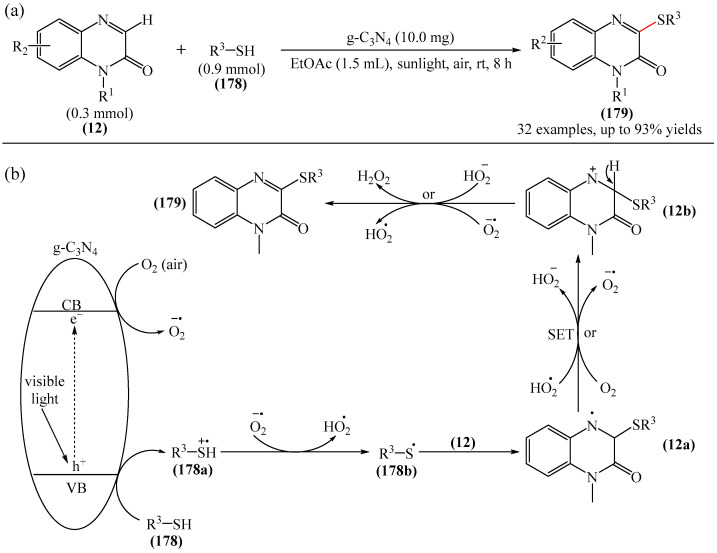
(**a**) g-C_3_N_4_-catalyzed sulfenylation of quinoxalin-2(1*H*)-ones, and (**b**) possible mechanism, according to [203]. R^1^ = H, Me, Et, Bu, C_6_H_5_, and others; R^2^ = H, F, Cl, Br, CF_3_, and others; R^3^ = *i*-Pr, *i*-Bu, CH_2_-C_6_H_5_, CH_2_-C_6_H_4_-Cl, and others.

Xie et al. [203] developed a method for the sulfenylation of quinoxaline-2(1*H*)-ones (**12**) using sunlight and recyclable graphitic carbon nitride (g-C_3_N_4_) as a heterogeneous photocatalyst (Figure 68a). The authors also investigated the possibility of using blue, green, violet and white light sources, but found that only sunlight led to the best yield of the desired products.

A tentative reaction mechanism was proposed (Figure 68b). Initially, g-C_3_N_4_ is excited by visible light and generates holes in the valence band (VB) and electrons in the conduction band (CB). The holes then receive an electron from thiol (**178**) to produce ation radical (**178a**) through a single-electron transfer process. Simultaneously, electrons in the CB are transferred to O_2_ (air) to form O_2_^•−^. O_2_^•−^ then abstracts hydrogen from the thiyl radical cation (**178a**) to deliver HO_2_^•^ particles and sulfanyl radical (**178b**), which is added to C=N in (**12**) to give the intermediate azide radical compound (**12a**). The latter undergoes further oxidation involving HO_2_^•^ or O_2_, producing the intermediate compound (**12b**). Finally, deprotonation of the nitrogen cation intermediate (**12b**) generates product **179**.

**Figure 69:**
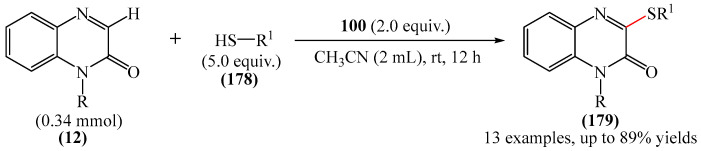
Selectfluor-mediated regioselective C−3 sulfenylation of quinoxalin-2(1*H*)-ones, according to [184]. R = H, Me, Rt, Bu, CH_2_-C_6_H_5_, COC_6_H_5_, and others; R^1^ = Et, Ac.

In addition to alkoxylation and amination reactions, sulfenylation reactions were also described in [184] (Figure 69). The series of products (**179**) obtained by the authors demonstrated the method’s effectiveness. The only significant drawback was the relatively high cost of Selectfluor (**100**).

### 6.2. Metal-Catalyzed Reactions

#### Pyrazines

**Figure 70:**
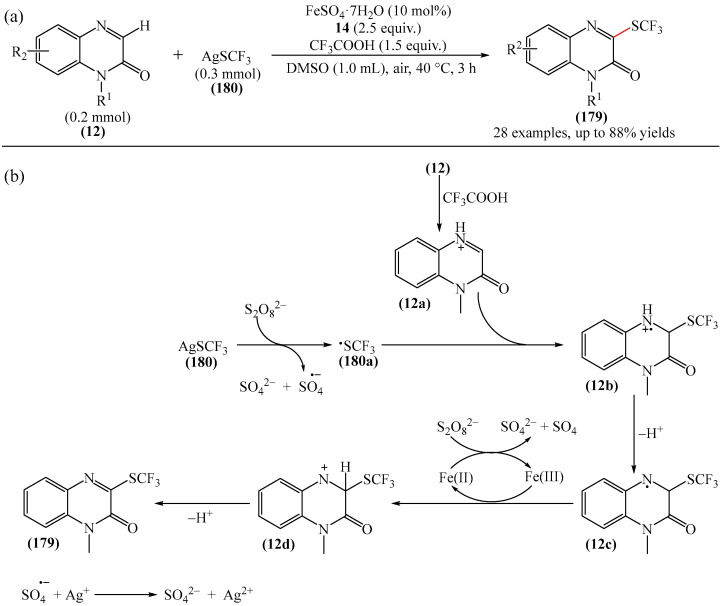
(**a**) Iron-catalyzed direct C3-H trifluoromethylthiolation of quinoxalin-2(1*H*)-ones, and (**b**) possible mechanism, according to [204]. R^1^ = F, Br, Me, CF_3_, and others; R^2^ = Me, Et, Pr, CH_2_-C_6_H_5_, and others.

Zou, Wu, and Wu et al. [204] implemented iron-catalyzed direct C3-H radical trifluoromethylthiolation of quinoxaline-2(1*H*)-ones (**12**). In this protocol, FeSO_4_·7H_2_O was used as a catalyst and AgSCF_3_ (**180**) as a trifluoromethylsulfanyllating reagent to obtain various SCF_3_-containing derivatives of quinoxaline-2(1*H*)-one (**179**) in yields of 26–88% (Figure 70a).

The reaction occurred in the presence of K_2_S_2_O_8_ as an oxidant, DMSO as a solvent, and CH_3_COOH as an additive. A plausible mechanism for the Fe-catalyzed trifluoromethylthiolation of quinoxaline-2(1*H*)-ones (**12**) using AgSCF*_3_* (**180**) was presented [204], as shown in Figure 70b. First, AgSCF_3_ is oxidized by K_2_S_2_O_8_ to form an SCF_3_ radical (**180a**). Peroxydisulfate anions are converted to sulfate dianions (SO_4_^2−^) and sulfate anion radicals (SO_4_^•−^) in the presence of silver(I) salts. Then SO_4_^•−^ can be transformed to SO_4_^2−^ in the presence of Ag(I). Meanwhile, (**12**) is more likely to be protonated with trifluoroacetic acid to produce intermediate (**12a**). Then the SCF_3_ radical (**180a**) attacks the C3 center of intermediate (**12a**) to deliver activated species (**12b**), which is further transformed into nitrogen-centered radical intermediate (**12c**). Subsequently, Fe(II) is oxidized by K_2_S_2_O_8_ to give Fe(III) species. The latter then oxidize the radical intermediate (**12c**) to afford the cationic intermediate (**12d**) and regenerate Fe(II). Finally, the cationic intermediate (**12d**) is deprotonated to produce the desired product **179**.

1-Methyl-3-(methylsulfonyl)quinoxalin-2(1*H*)-one can hypothetically be synthesized on the data [205]. This product can be formed during an Fe-catalyzed interaction between C−3-methylated quinoxalin-2(*1H)*-ones and dimethyl sulfoxide in air, under irradiation with a 23 W CFL at room temperature for 20 min, in the presence of H_2_O_2_ (**53**) as an oxidizing agent.

## 7. Formation of C−Cl Bonds

### 7.1. Metal-Catalyzed Reactions

#### Pyrazines

**Figure 71:**
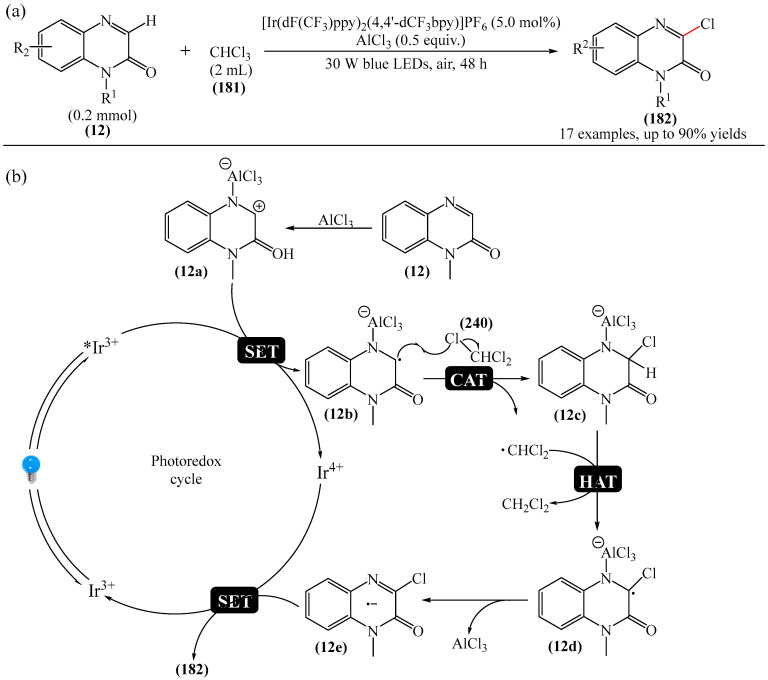
(**a**) Photoredox-catalyzed chlorination of quinoxalin-2(1*H*)-ones, and (**b**) possible mechanism, according to [206]. R^1^ = Me, Et, *n*-Bu, Bn, and others; R^2^ = H, F, Cl, Br, and others.

Chen and Yang et al. [206] developed a method for the photoredox-catalyzed chlorination of quinoxaline-2(1*H*)-ones (**12**) using CHCl_3_ (**181**) as a chlorine source (Figure 71a).

The optimal photocatalyst in this case was Ir(dF(CF_3_)ppy)_2_(4,40-dCF_3_bpy)]PF_6_ with AlCl_3_ as an additive, when irradiated with 30 W blue LEDs for 24 h. Here, AlCl_3_ can serve as an additional source of chlorine, alongside CHCl_3_. The tentative mechanistic rationale of the process was also provided (Figure 71b). When irradiated with a blue LED, [Ir(dF(CF_3_)ppy)_2_(4,40-dCF_3_bpy)]PF_6_^+^ reaches a photoexcited state, which is then quenched by the intermediate adduct (**12a**) of AlCl_3_-quinoxalinone through a SET (single-electron transfer) process to form radical (**12b**) and oxidant [Ir(dF(CF_3_)ppy)_2_(4,40-dCF_3_bpy)]PF_6_^2+^. Then, the H–Cl exchange between intermediate (**12b**) and chloroform (**181**) proceeds smoothly through sequential chlorine atom transfer and hydrogen atom transfer to produce intermediate (**12d**). Finally, the pai radical anion (**12e**) formed by the dissociation of intermediate (**12d**) is oxidized by [Ir(dF(CF_3_)ppy)_2_(4,40-dCF_3_bpy)]PF_6_^2+^ to produce the final product **182**.

## 8. Formation of C−Se Bonds

### 8.1. Metal-Free Reactions

#### Pyrazines

**Figure 72:**
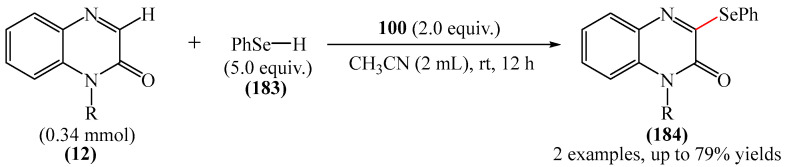
Selectfluor-mediated regioselective C−3 selanylation of quinoxalin-2(1*H*)-ones, according to [184]. R = H, Me.

The selenylation of quinoxaline-2(1*H*)one (1a and 1b) with benzene selenol (10a) in the presence of Selectfluor (**100**) was carried out [184] (Figure 72). 3-(Phenylselanyl)quinoxalin-2(1*H*)-one and 1-methyl-3-(phenylselanyl)quinoxalin-2(1*H*)-one were isolated in yields of 75% and 79%, respectively. The mechanism of this process is similar to the mechanism shown in Figure 53b.

## 9. Conclusions

Although not exhaustive, this review provides an overview of the most promising methods for the functionalization of C−H bonds in polyaza six-membered heterocycles, with diazine and triazine skeletons serving as examples. These methods, which primarily rely on transition metal catalysts or organic oxidants, have attracted significant attention in recent decades. Consequently, a variety of direct, efficient and straightforward processes have been developed that enable the selective introduction of a wide range of functional groups into six-membered polyaza heteroarene molecules. The regioselectivity of these processes can be controlled by the nature of both the *N*-heterocycles and the substrates involved. The review also discusses the mechanistic aspects of individual reactions, providing an in-depth understanding of the processes described. The presented information enables us to conclude that methods for the direct C−H functionalization of six-membered polyaza heteroarenes are of paramount importance in modern organic synthesis.

Despite the advances that have been made, a significant drawback of virtually all the methodologies described cannot be overlooked: the issue of expanding their synthetic potential. Therefore, considerable effort is still needed to develop more efficient, widely applicable processes that can completely overcome the limitations of modern approaches. Furthermore, many of the proposed mechanisms are based solely on literature data or, at best, on the kinetic analysis of the processes under study. In some cases, the explanation of the mechanism does not correspond to the laws of chemical thermodynamics and kinetics. Researchers are often unable to expand the range of transformable molecules or scale them up to at least gram quantities precisely because of the incorrect mechanistic interpretations of the reactions. It can therefore be assumed that, in the near future, there will be a significant increase in the number of scientific papers resulting from collaboration between specialists in organic synthesis, physicochemical research methods and catalysis. This collaboration will enable the synthesis of new functionalized six-membered polyaza heteroarenes and allow each stage of the synthetic process to be investigated in detail. It will also confirm the existence of certain transition states or intermediates during the production of the target product and explain the physical chemistry requirements for scaling up production, even for small quantities.

Additionally, we would like to point out that the next part of our review will address issues of C−X functionalization (where X = halogen, nitrogen, etc.). This is because these techniques are equally important to the modern synthesis of biologically active and pharmacophore molecules as the methods of C—H functionalization of six-membered polyaza heteroarenes discussed in this work.

## Figures and Tables

**Table 1:** Comparative table of different techniques for alkylation reactions.

№№	Original Heterocycle	Reaction Conditions	Products	Notes
The formation of the C−C bonds (metal-free)				
1.	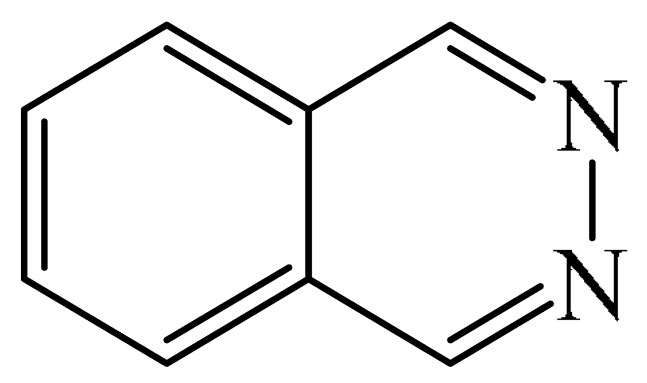 (1.0 equiv.)	isobutyric acid (3.0 equiv.), PIFA (2.0 equiv.), MesAcr (1.0 mol%), MeCN (0.1 mol), 32 W blue LED light (λ_max_ = 455 nm), rt, 3–16 h.	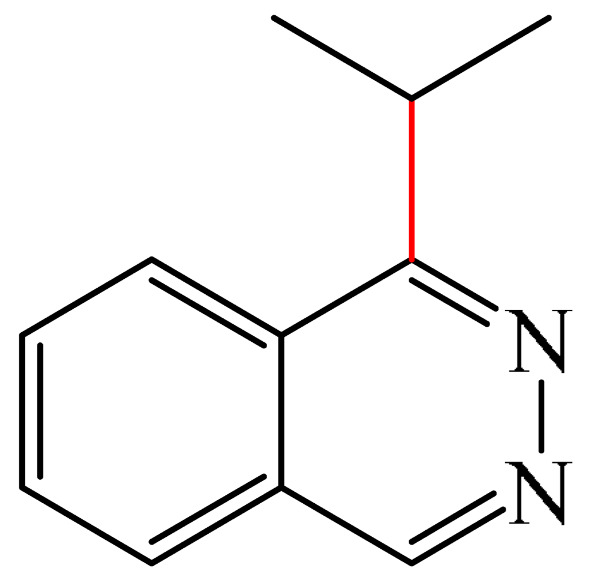 40%, 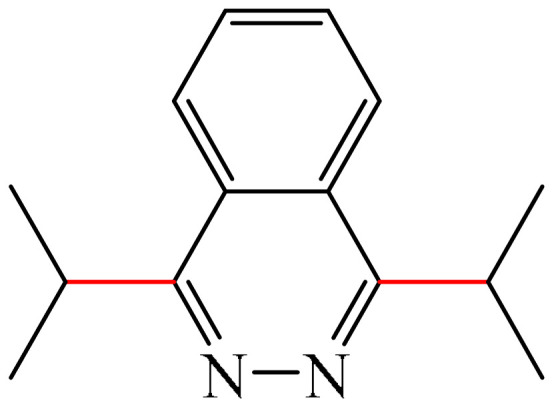 30%	
2.	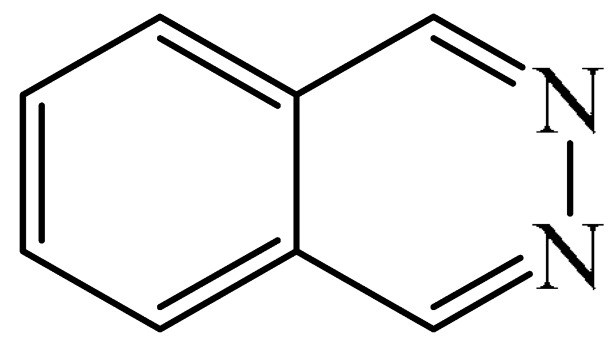 (0.2 mmol)	isopropylboronic acid (1.0 mmol), 1.0 atm O_2_, TFA (0.4 mmol) DCE (0.5 mL), 110 °C.	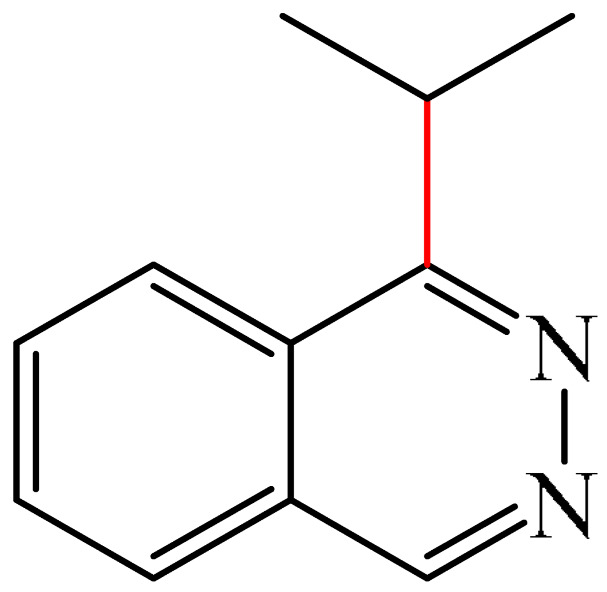 68%, 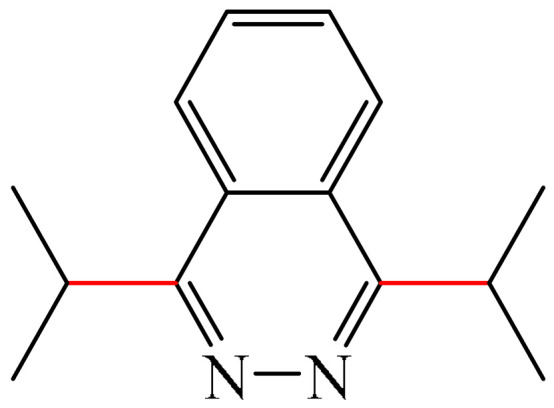 38%	
3.	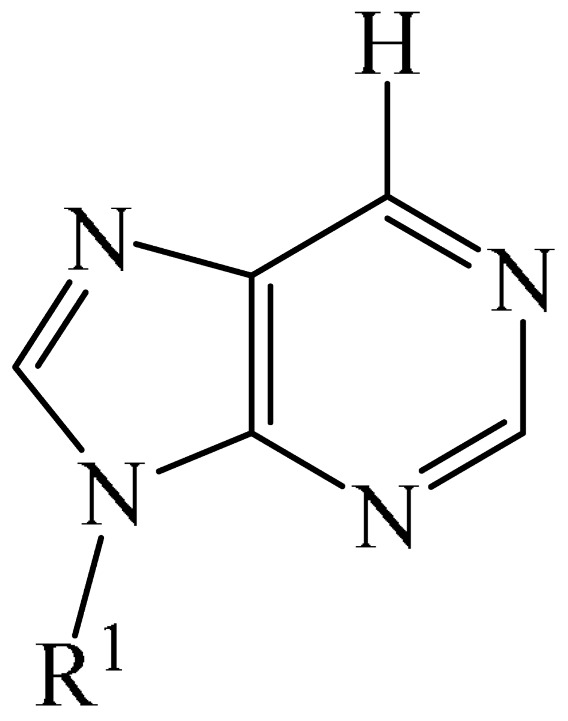 (0.5 mmol)	alkenes (1.0 mmol), NaBH_4_ (2 mmol), Fe(NO_3_)_3_-9H_2_O (5 equiv.), CH_3_CN (5 mL) + EtOH (5 mL), 0 °C, 40 min, then, O_2_ ballon, 70 °C, 1 h.	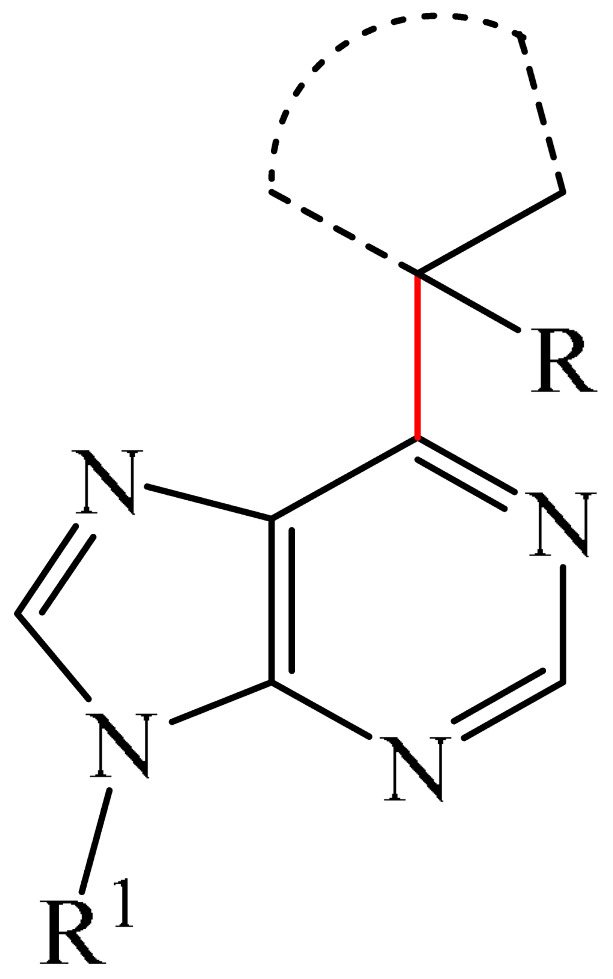 41 examples, up to 91% yields	R = H, Me, Et, and others; R^1^ = Bn, Bu, Pr, C_6_H_5_, C_6_H_5_-CH_2_, and others.
4.	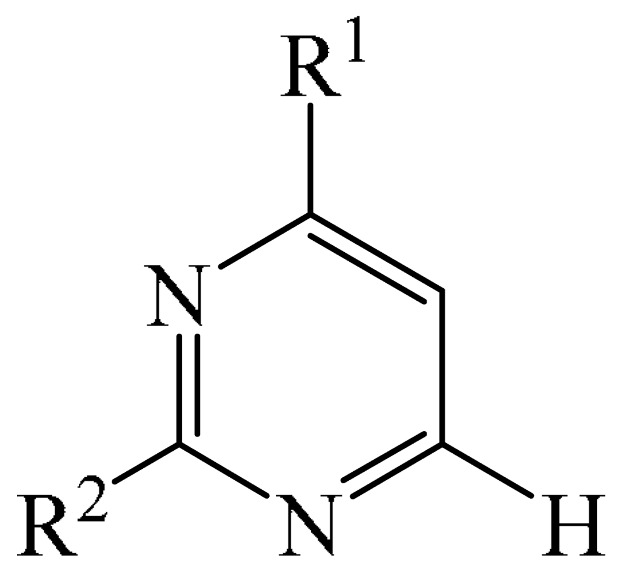 (0.1 mmol)	R^3^–H (30 equiv.), 4CzIPN (10) (2 mol%), TFA (3 equiv.), CHCl_3_ (0.5 mL) + MeCN (2.5 mL), 20 W 460–465 nm LEDs, N_2_, 25 °C.	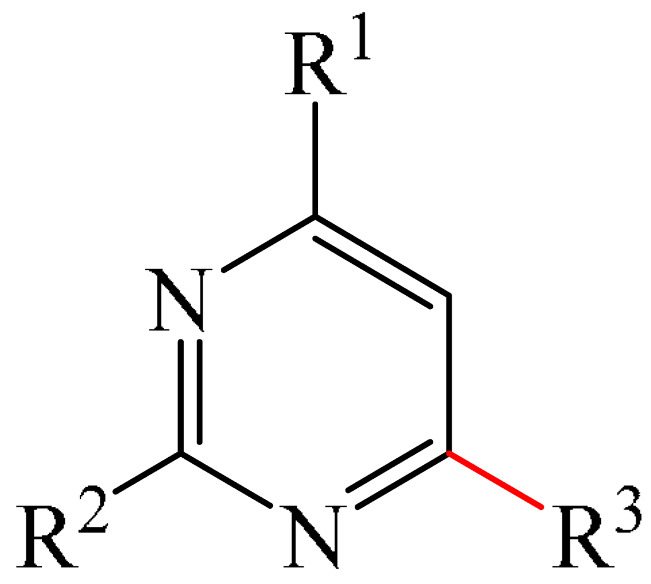 27 examples, up to 97% yields	R^1^ = R^2^ = H, Me, Cy, C_6_H_5_, Cl, and others; R^3^ = C_6_H_5_, C_6_H_5_-CH_2_-, HO-CH_2_-, and others.
5.	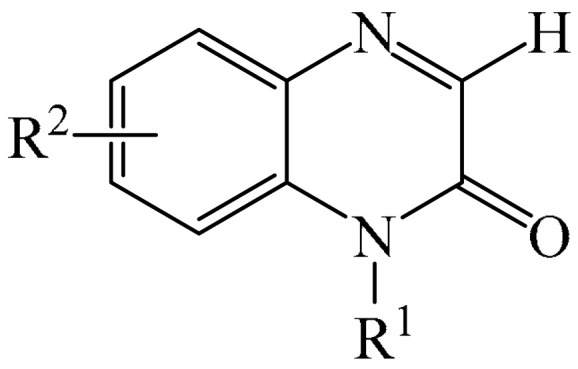 (0.5 mmol)	pyrazolones (0.75 mmol), K_2_S_2_O_8_ (1.0 mmol), CH_3_CN (2 mL), rt, under air, 1–8 h.	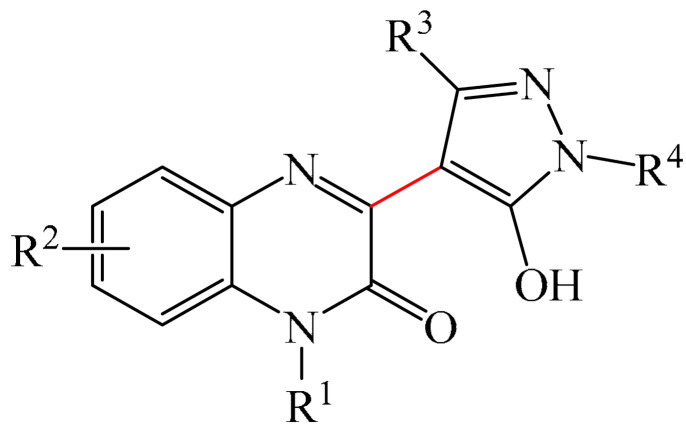 22 examples, up to 98%	R^1^ = H, Me, NO_2_, Cl, Br; R^2^ = H, Et, OEt, *n*-C_5_H_11_, *i*-Pr, and other; R^3^ = Me, *n*-Pr, *i*-Pr, C_6_H_5_; R^4^ = C_6_H_5_, 4-ClC_6_H_4_, 2-ClC_6_H_4_, 4-MeOC_6_H_4_, and others.
6.	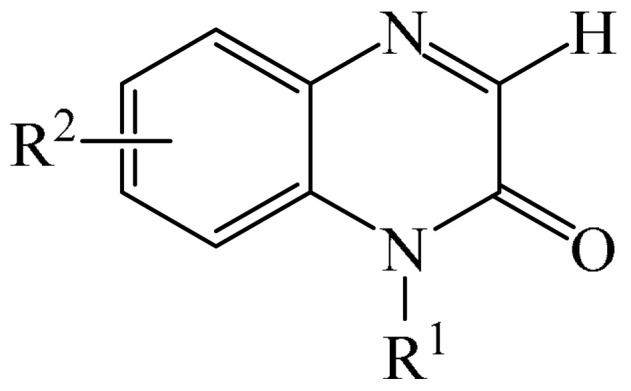 (0.2 mmol)	simple ethers (2.0 mL), Rose Bengal (1 mol%), DABCO (1 equiv.), TBHP (1 equiv.), 3 W blue LEDs, air, rt, 24 h.	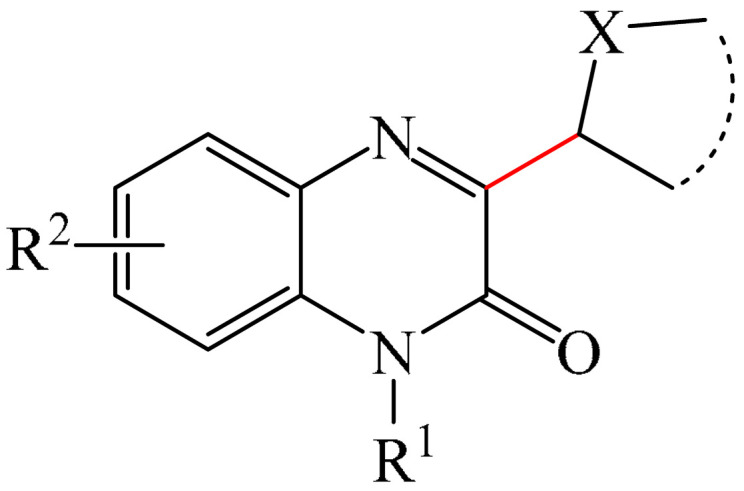 27 examples, up to 90%	R^1^ = Me, Et, *n*-Pr, *n*-Bu, Ph, Ph-CH_2_-, and other; R^2^ = H, Me, F, Cl, Br, CN.
7.	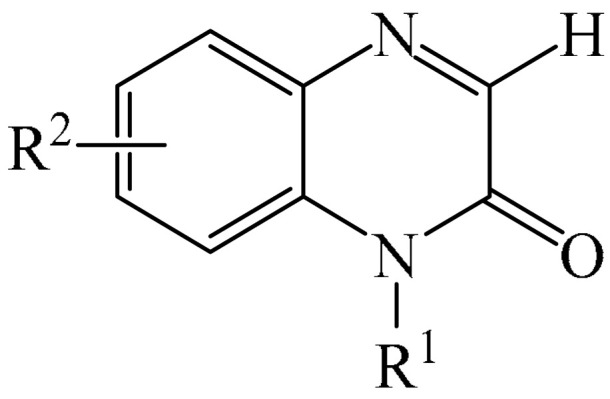 (0.2 mmol)	α,α-difluoroarylacetic acids (0.24 mmol), (NH_4_)_2_S_2_O_8_ (0.6 mmol), DMSO (2.0 mL), N_2_, 60 °C, 5 h.	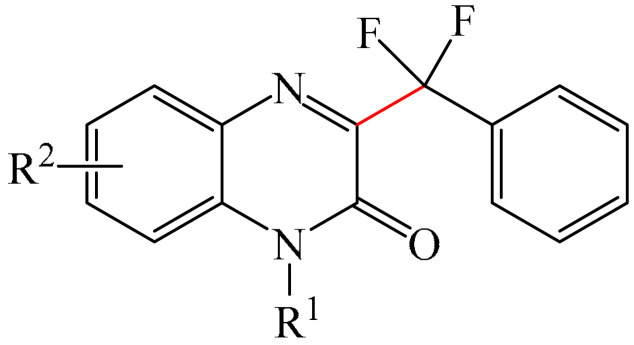 31 examples, up to 85%	R^1^ = H, Me, Et, *n*-Pr, *n*-Bu, C_6_H_4_-CH_2_-, and others; R^2^ = H, Me, F, Cl, Br, NO_2_.
8.	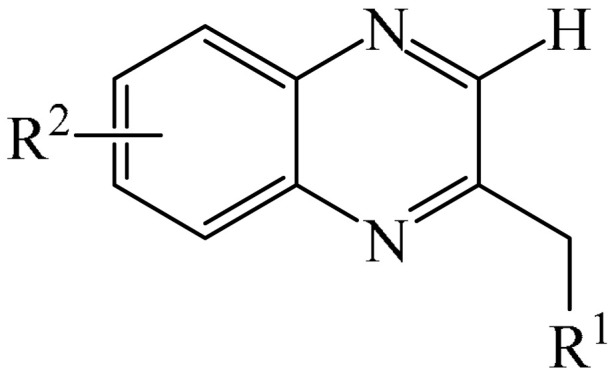 (1.0 mmol)	alcohols (0.5 mmol), TfOH (10 mol%), dioxane (1 mL), 120 °C, 48 h.	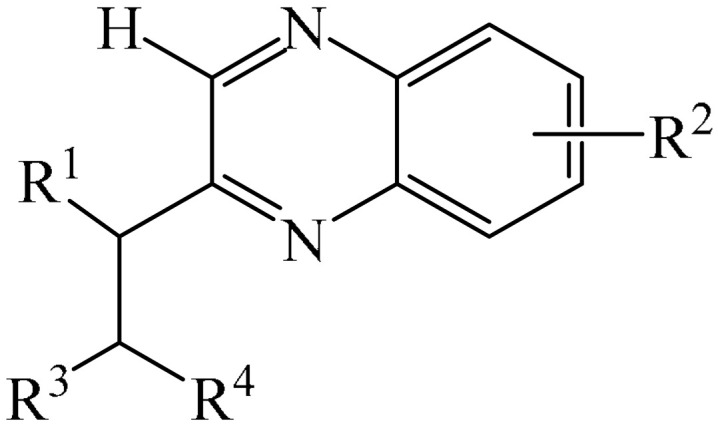 11 examples, up to 94% yields	R^1^ = H or SO_2_ C_6_H_5_; R^2^ = H or NO_2_; R^3^ = C_6_H_5_, 2-FC_6_H_4_, 2-MeC_6_H_4_, 3-MeOC_6_H_4_, 4-MeOC_6_H_4_, and others; R^4^ = ferrocene-functionalized molecules, 4-MeOC_6_H_4_, and others.
9.	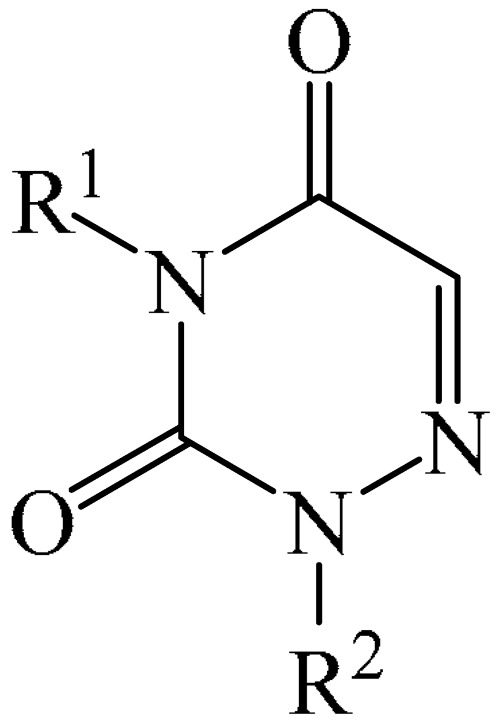 (0.3 mmol)	secondary amines (0.6 mmol), 10 (5 mol%), Pyruvic acid (1.5 equiv.), DCM (3.0 mL), rt, 20 W blue LED, 12 h.	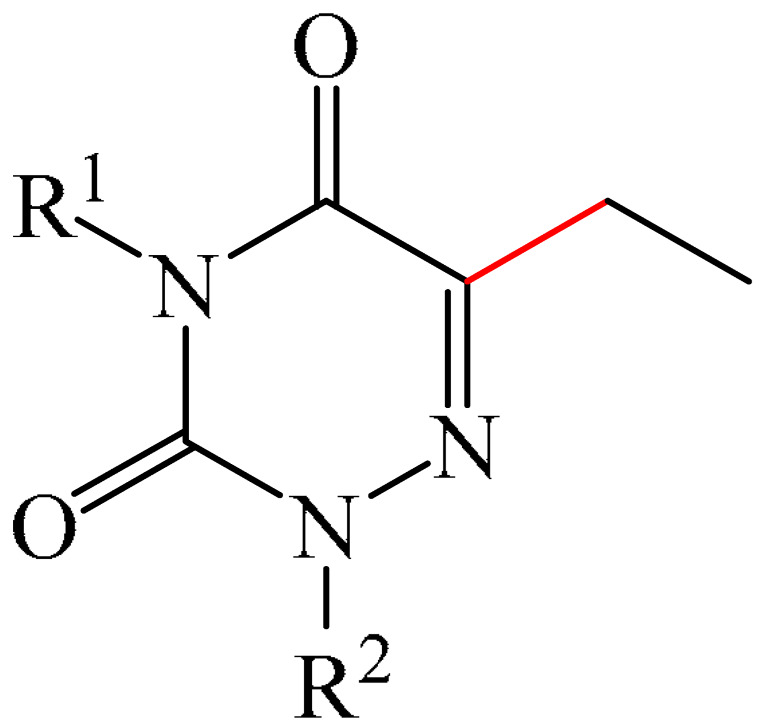 24 examples, up to 91% yields	R^1^ = H, Me, Bn, 4-FC_6_H_4_, 4-MeC_6_H_4_, and others; R^2^ = Me, Bn, C_6_H_5_-CH_2_, C_6_H_5_, CH_2_CH=CH_2_, and others.
10.	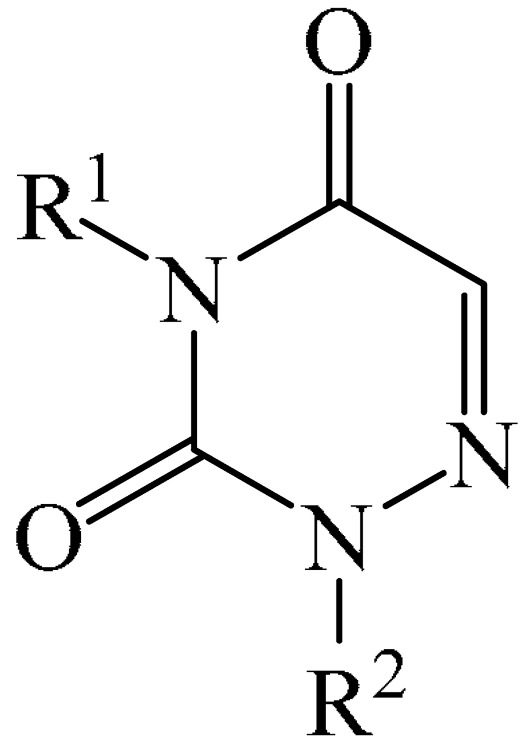 (0.1 mmol)	alkyl halide (2.0 equiv.), (+)GF|Ni(-), nBu_4_NClO_4_ (1.0 equiv.), Et_3_N (3.0 equiv.), I = 6 mA, TFE (4.0 mL), rt, 16–36 h.	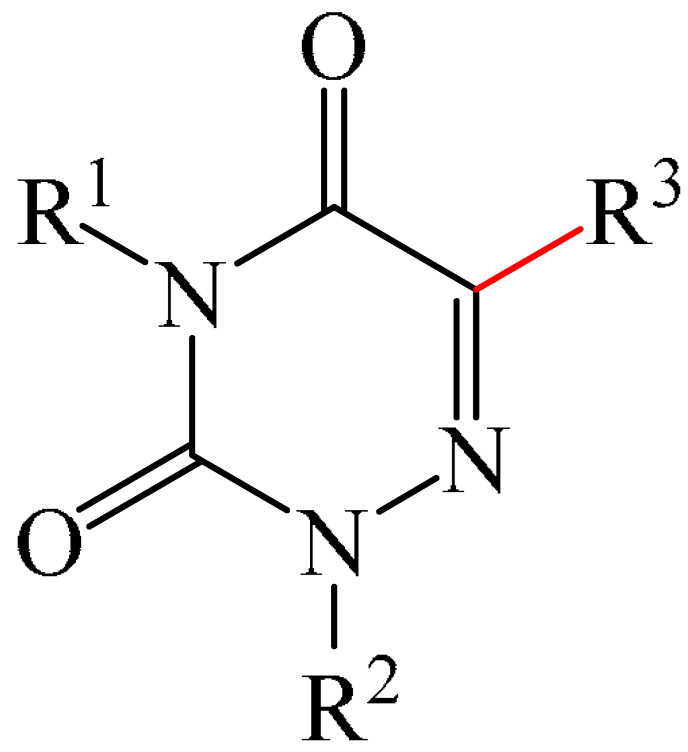 24 examples, up to 94% yields	R^1^ = H, *n*-Bu, and others; R^2^ = Bn, C_6_H_5_, and others; R^3^ = C_6_H_5_, *n*-Bu, *i*-Pr, and others; X = Br, I.
11.	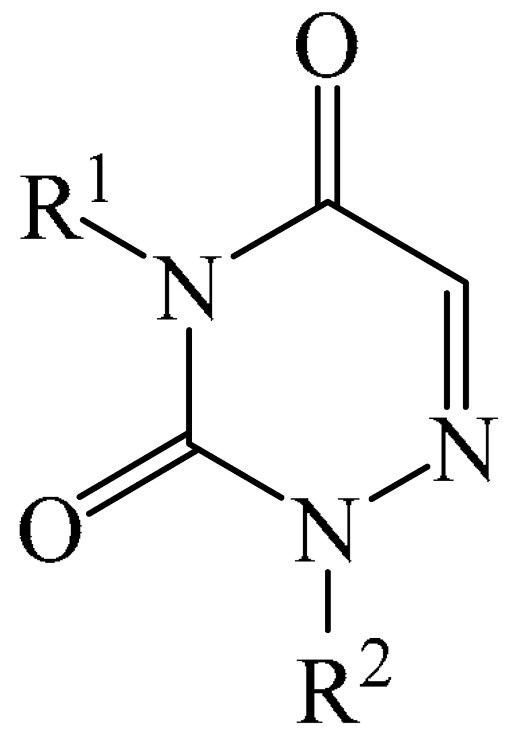 (0.3 mmol)	ethers (2.0 mL), 2-t-Bu-AQN (3.0 mol%), Cs2CO3 (0.5 equiv.), 25 W blue LED, air, rt, 8 h.	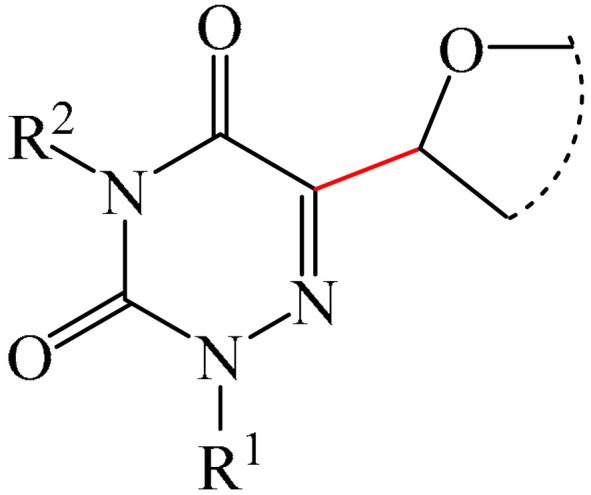 44 examples, up to 92% yields	R^1^ = R^2^ = H, Me, *n*-Pr, CH_2_-CH=CH_2_, and others.
12.	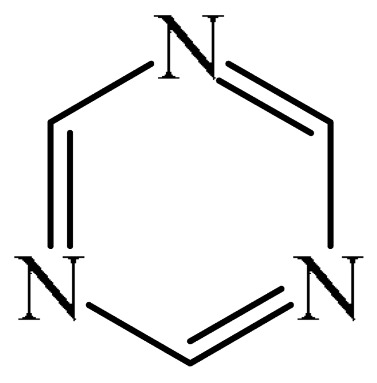 (0.2 mmol)	Cyclohexane (5.0 equiv.), TsNHMe (15 mol %), PIFA (2.3 equiv.), DCM (2 mL), N_2_, 2 × 50 W blue LEDs, rt, 6 h.	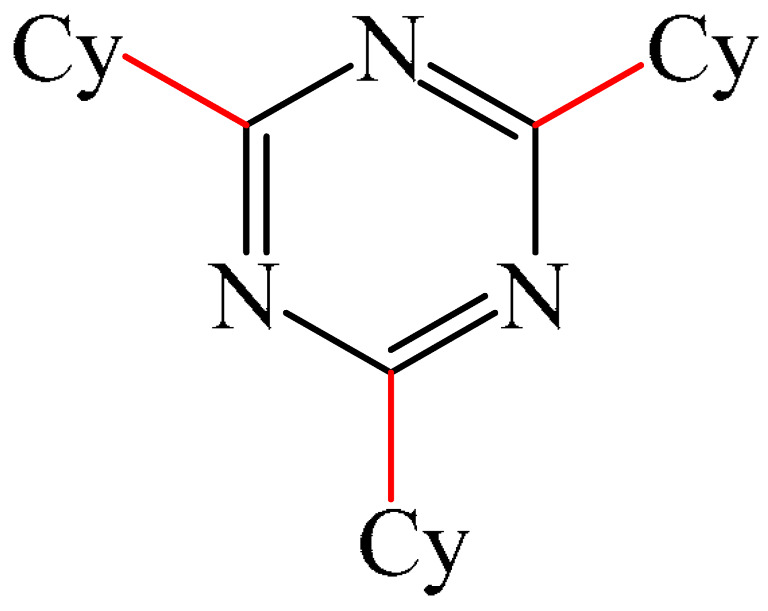 90% yields	
The formation of the C−C bonds (metal-catalyzed)				
13.	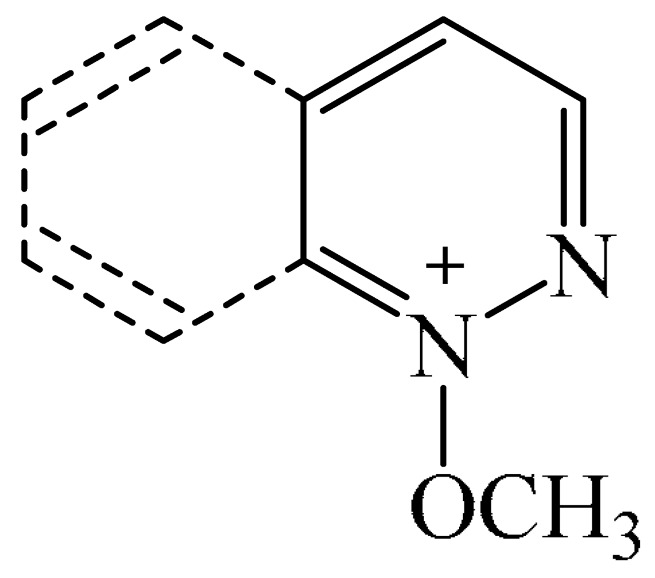 (5.0 equiv.)	alkenes (250 μmol), Co(acac)_2_ (1.0 equiv.), TBHP (1.0 equiv.), Et_3_SiH (5.0 equiv.), CH_2_Cl_2_ (0.2 M), 24 °C, 48 h.	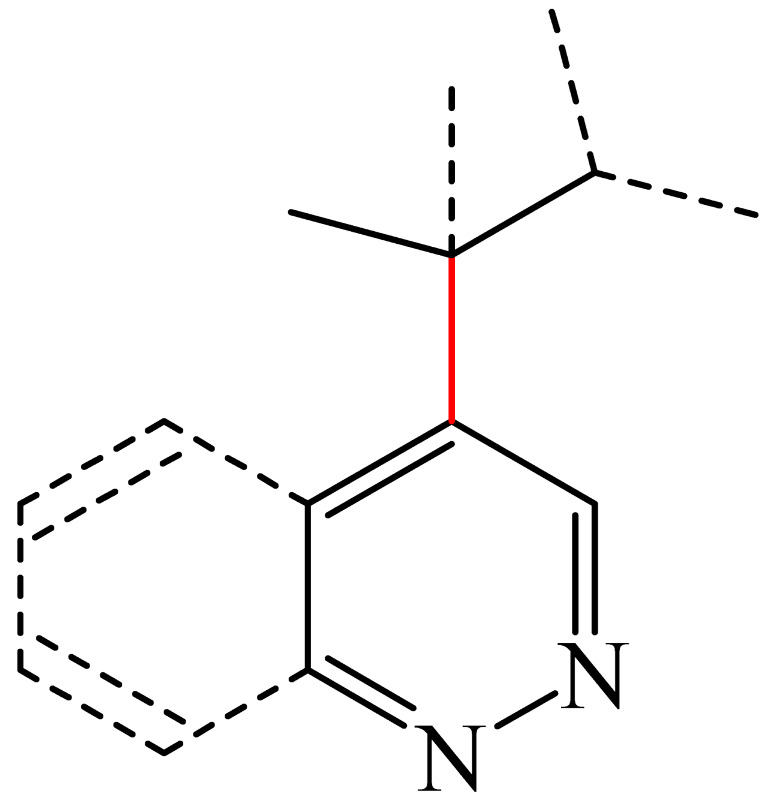 4 examples, up to 51% yield	
14.	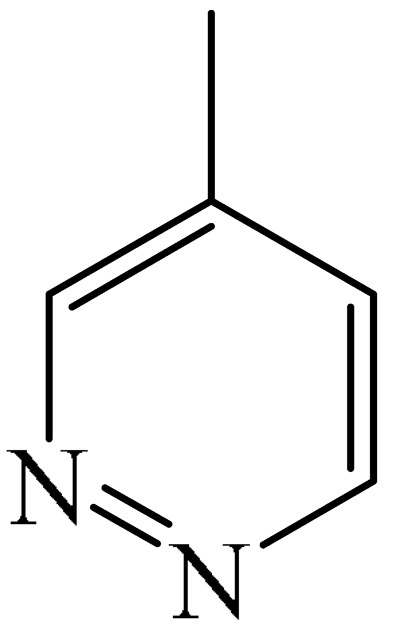 (1.0 equiv.)	(1,2-dicyclohexylethyl)boronic acid (1.5 equiv.), [Rh(cod)Cl]_2_ (5.0 mol%), dppe (12.5 mol%), KOPiv (25.0 mol%), toluene (0.6 M), MW, 190 °C, 48 h, banchtop set-up.	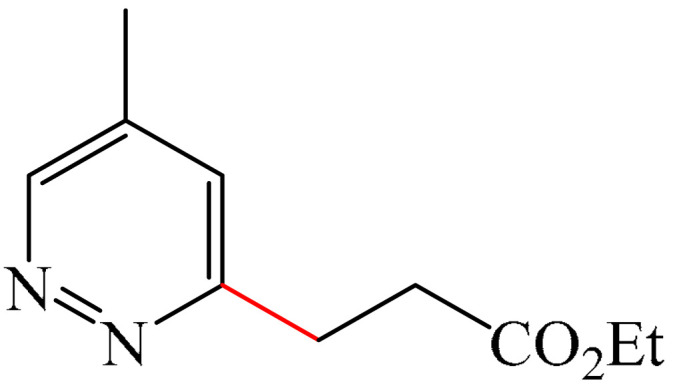 31% yield	
15.	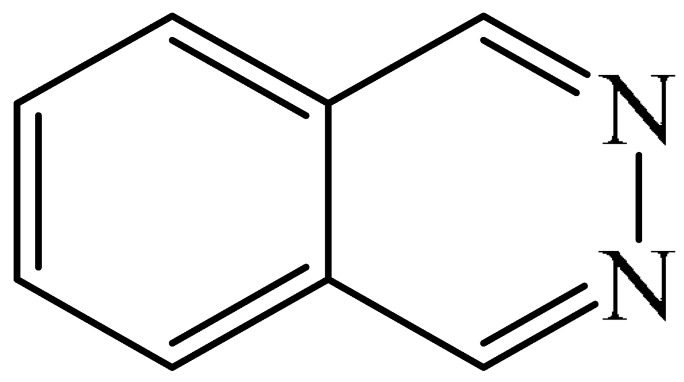 (1.0 equiv.)	bromocyclohexane (2.0 equiv.), Ir[dF(CF_3_)ppy]_2_(dtbbpy)PF_6_ (1 mol%), TTMS (2.0 equiv.), TFA (2.0 equiv.), acetone (0.1 M), 36 W blue LED, O_2_, rt, 24 h.	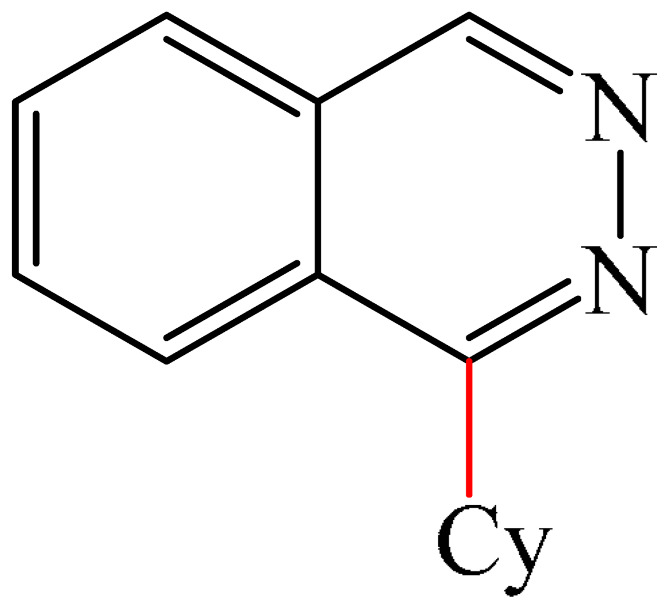 46% yield	
16.	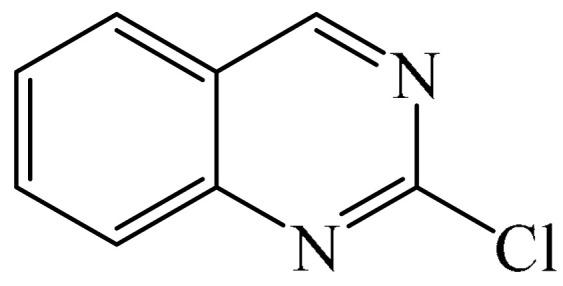 (1.0 equiv.)	bromocyclohexane (2.0 equiv.), Ir[dF(CF_3_)ppy]_2_(dtbbpy)PF_6_ (1 mol%), TTMS (2.0 equiv.), TFA (2.0 equiv.), acetone (0.1 M), 6 W blue LED, O_2_, rt, 24 h.	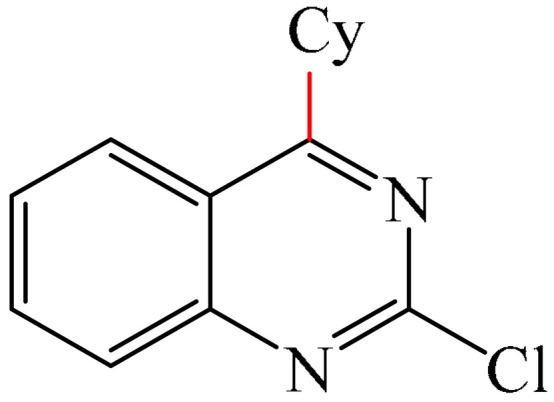 56% yield	
17.	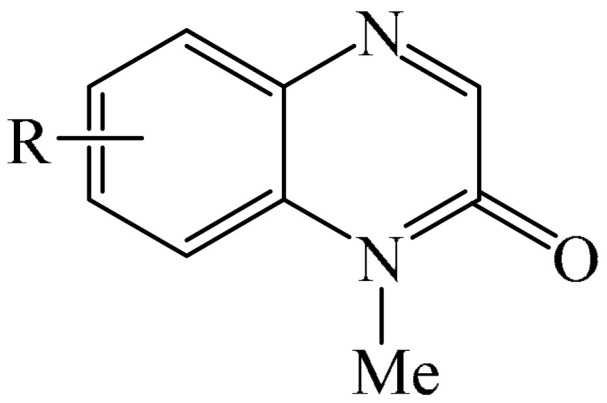 (0.2 mmol)	Fe(OAc)_2_ (5.0 mol%), HOAc (1.0 equiv.), DMSO/H_2_O (9/1, 2.0 mL), N_2_, 100 °C, 12 h	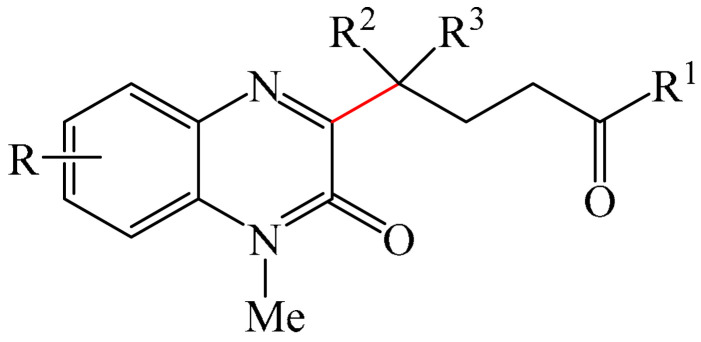 40 examples, up to 98%	R = H, Me, OMe, F, Cl, Br, NO_2_, and others; R^1^ = H, Me, and others; R^2^ = R^3^ = H, Me, C_6_H_5_, and others.
18.	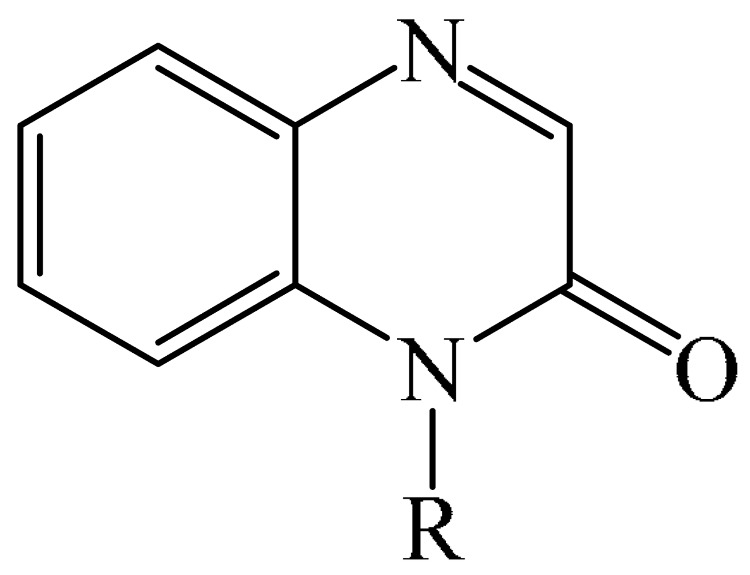 (0.1 mmol)	carboxylic acids (0.2 mmol), CeCl_3_ (10 mol%), KO*t*-Bu (20 mol%), CH_3_CN (0.6 mL), rt, 24 h, blue-violet LEDs (427 nm).	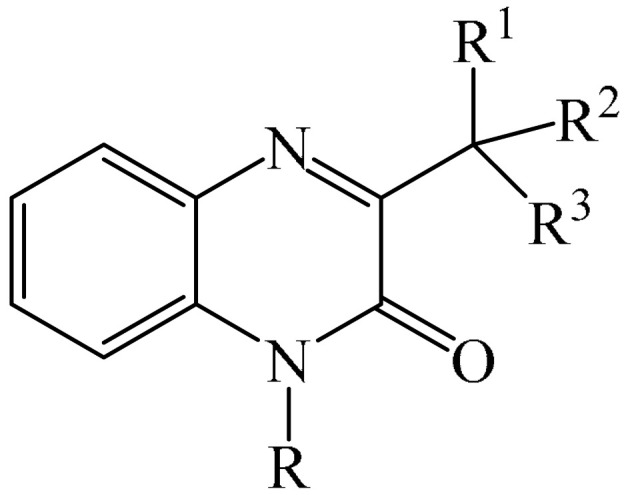 42 examples, up to 98% yields	R = H, Me, 4-butenyl, and others; R^1^ = H, Me, Et, C_6_H_5_, and others; R^2^ = H, Me, Et, and others; R^3^ = H, C_6_H_5_, and others.
19.	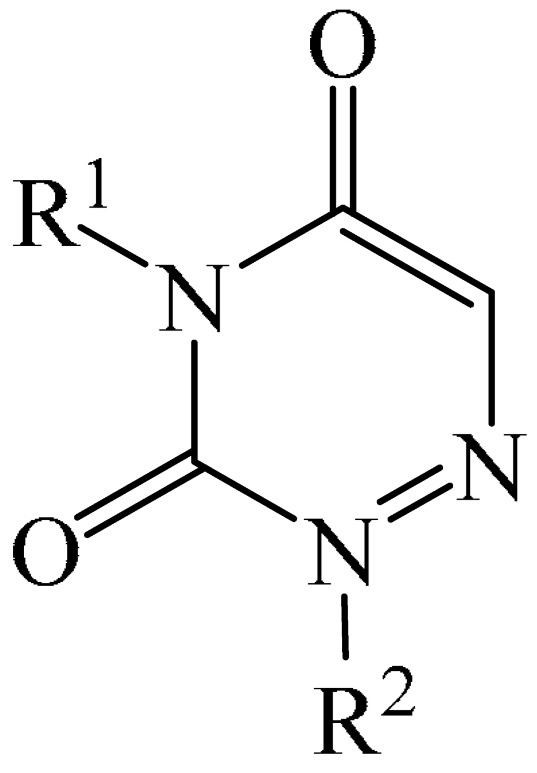 (0.3 mmol)	*N*-methylanilines (0.9 mmol), Ru(bpy)_3_Cl_2_·6H_2_O (2.0 mol %), KO*t*Bu (1.0 equiv.), DMF (1.0 mL), air, rt, 25 W blue LEDs, 12 h.	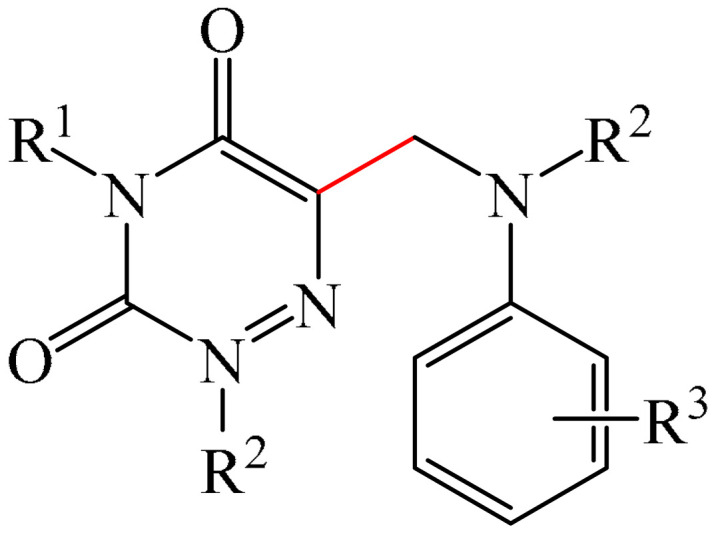 15 examples, up to 80% yields	R^1^ = Me, CH_2_-C_6_H_5_, CH_2_-C_6_H_4_-4F, and others; R^2^ = H, Me, Et, *n*-Bu, and others; R^3^ = H, Me, OMe, Et, and others.
20.	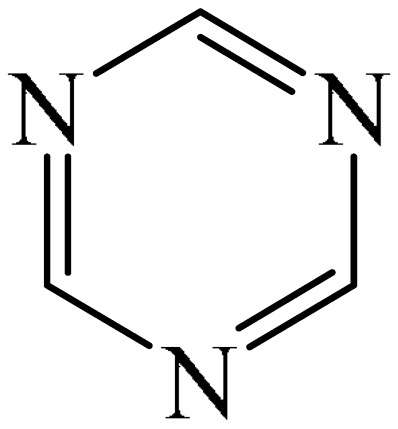 (0.1 mmol)	adamantyl iodide (0.1 mmol), [Pd(PPh_3_)_4_] (10 mol%), dppp (14 mol%), Cs_2_CO_3_ (2 equiv.), PhCF_3_ (0.6 mL), Ar, 110 °C, 24 h.	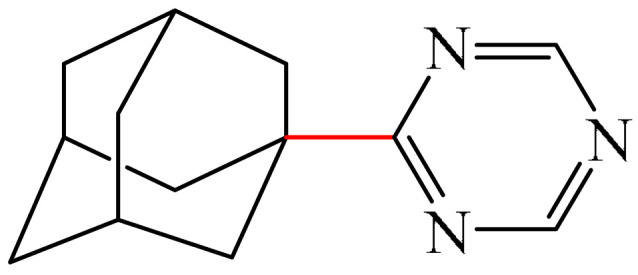 64% yields	

## Data Availability

No new data were created or analyzed in this study. Data sharing is not applicable to this article.

## Abbreviations

The following abbreviations are used in this manuscript:

ACN	Acetonitrile
BI-OAc	Acetoxybenziodoxole
BPO	Benzoyl peroxide
CB	Conduction band
4CzIPN	1,2,3,5-tetrakis(carbazol-9-yl)-4,6-dicyanobenzene
CFL	Compact fluorescent lamp
cod	1,5-cyclooctadiene
DABCO	(1,4-diazabicyclo [2.2.2]octane)
DBU	1,8-diazabicyclo [5.4.0]undeC−7-ene
DCE	1,2-dichloroethane
DCM	Dichloromethane
DDC	Diethyldithiocarbamate
DDQ	2,3-dichloro-5,6-dicyanobenzoquinone
DIPEA	*N,N*-Diisopropylethylamine
DMFA	Dimethylformamide
DMSO	Dimethyl sulfoxide
4DPAIPN	Substituted benzene-1,3-dicarbonitrile or 1,3-dicyano-2,4,5,6-tetrakis(diphenylamino)-benzene
dppe	1,2-bis(diphenylphosphino)ethane
DTBP	Di-tertbutyl peroxide
FDA	Food and Drug Administration
EDA	Electron donor-acceptor
EtOAc	Ethyl acetate
HAT	Hydrogen atom transfer
HFIP	Hexafluoroisopropanol
HRMS	High-resolution mass spectrometry
IBA-N_3_	Iodonium-based Azide-N_3_
LED	Light-emitting diode
MesAcr	9-mesityl-10methyl acridinium
MOF	Metal–organic framework
MVEC	Microvascular endothelial cells
MW	Microwave
NHPI	*N*-(acyloxy)phthalimides
NMP	*N*-Methyl-2-pyrrolidone
OTf	Triflate group
PFBI-OH	Perfluorinated hydroxybenziodoxole
PIFA	bis(trifluoroacetoxy)iodo benzene
PMDETA	*N,N,N′,N′, N″*-pentamethyldiethylenetriamine
TBAB	Tetrabutylammonium bromide
TBHP	*tert*-butylhydroperoxide
temp	Temperature
TFA	Trifluoroacetic acid
TfOH	Trifluoromethanesulfonic acid
TPPT	Triphenyl phosphorothionate
2-*t*-Bu-AQN	2-*tert*-Butylanthraquinone
SCE	Saturated calomel electrode
VB	Valence band
US	Ultrasound
Δ	Boiling or reflux
*	Optical active fragment or center
